# Discovery of papyifuran A as an unusual cembrane diterpenoid from *Boswellia papyrifera* resin reveals EEF2 as a potent new drug target for fibrosis of multiple organs

**DOI:** 10.1016/j.apsb.2025.04.004

**Published:** 2025-04-08

**Authors:** Madhu Babu Sura, Yeting Zhou, Jijun Li, Yongxian Cheng

**Affiliations:** Guangdong Provincial Key Laboratory of Chinese Medicine Ingredients and Gut Microbiomics, Marshall Laboratory of Biomedical Engineering, Institute for Inheritance-Based Innovation of Chinese Medicine, School of Pharmacy, Shenzhen University Medical School, Shenzhen University, Shenzhen 518060, China

**Keywords:** *Boswellia papyrifera*, Resin, Papyifurans A‒C, Fibrosis, EEF2, PROTAC, DARTS, ABPP

## Abstract

Chronic kidney disease (CKD) affects 8%–15% of the population globally and can cause renal failure, partly due to lack of effective treatments and drug targets. Three novel cembrane diterpenoids papyifurans A‒C (**1**–**3**), in particular of **1** with an unprecedented trioxatetracyclo[10.2.1.1^2,5^.1^6,9^]heptadecane polyether scaffold, derived from *Boswellia papyrifera* resin, were found to effectively protect against renal fibrosis *in vitro* and *in vivo*. Their structures were fully characterized using a combination of spectroscopic, computational, modified Mosher’s, and X-ray crystallographic analysis. In particular, we performed chemical proteomic analyses and found that Elongation factor 2 (EEF2) is the key target of compound **1** for anti-renal fibrosis *in vitro*. Moreover, previous studies have linked EEF2 with lung fibrosis, while compound **1** was found to inhibit the hallmarks of organ fibrosis *in vitro.* Such effects were observed to decrease with the knock down of EEF2 *in vitro*, suggesting that EEF2 might be a universal drug target of organ fibrosis. Collectively, the present study demonstrated an example of identifying drug targets by using structurally novel natural products, which will be beneficial for developing therapeutic agents against organ fibrosis.

## Graphical abstract

Three novel cembrane diterpenoidal skeletons, papyifurans A‒C, were isolated from *Boswellia papyrifera* exudates. Chemical proteomics approaches revealed that anti-fibrotic effects of papyifuran A are associated with EEF2, which was also found to be a potential pharmacological target of multiple organ fibrosis by siRNA interference technique.

**Figure:**
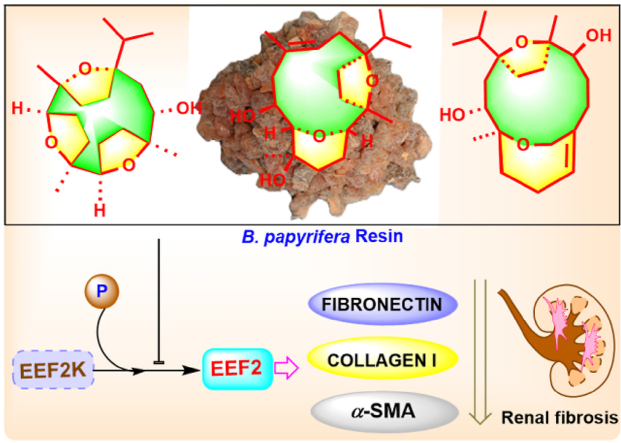

## 1 Introduction

Renal fibrosis is indeed a widespread global health issue and is recognized as a leading contributor to renal failure. Renal fibrosis is manifested by primary and secondary glomerulosclerosis, tubular interstitial fibrosis, and excessive deposition of the extracellular matrix (ECM), which gradually destroys and replaces the functional renal parenchyma, leading to organ failure^1^. Fibrosis represents the progression of a variety of kidney diseases to renal failure and then to the stage of end-stage renal disease (ESRD), and there are few treatments other than renal replacement and dialysis. In recent years, the mortality rate of fibrotic diseases has been rising, accounting for about 45% of the total mortality rate in developed countries. Unfortunately, no approved anti-renal fibrotic therapies exist, emphasizing the pressing need for extensive investigations into anti-fibrotic treatments^2^^,^^3^. In the face of the predicament that no therapeutic target for renal fibrosis has been discovered yet and there are almost no drugs available in the clinic, the necessity and importance of finding molecular targets for renal fibrosis is indisputable to break the bottleneck limiting the development of renal fibrosis drugs.

Traditional medicine offers effective treatment for complex diseases, and certain traditional Chinese medicine (TCM), like *Periplaneta americana*, shows anti-fibrotic properties^4^^,^^5^. Additionally, studies indicate that it may help in reducing organ damage by regulating fibroblast activation, cytokine secretion, and ECM deposition^6^, ^7^, ^8^, ^9^. Still, the therapeutic mechanisms and targets remain unknown. The resin of *Boswellia serrata* has been used in human trials, along with curcumin as a supplement, to evaluate the antioxidant and inflammatory responses, including in CKD patients^10^, ^11^, ^12^, ^13^, ^14^. In recent years, our research team has focused on finding anti-fibrotic potentials from natural products and their derivatives, in our quest to identify small molecule compounds that exhibit long-term resistance to renal fibrosis^15^, ^16^, ^17^, several natural products have demonstrated promising anti-fibrotic properties. However, they often have unclear action mechanisms or targets, which frequently leads to discontinuation of the study. In the past few decades, scientists have verified various means to find the targets of small molecular drugs, the most commonly used method is labeling, where small molecules are transformed into probes. These probes are then used to enrich proteins for the next step of identification through pull-down technique and other experiments^18^. Among them, activity-based protein profiling (ABPP) is a popular method for target labeling and identification. ABPP has been widely used in the screening of bio-active natural products for the validation of natural small-molecules’ target proteins^19^, ^20^, ^21^, ^22^, ^23^.

**Figure 1:**
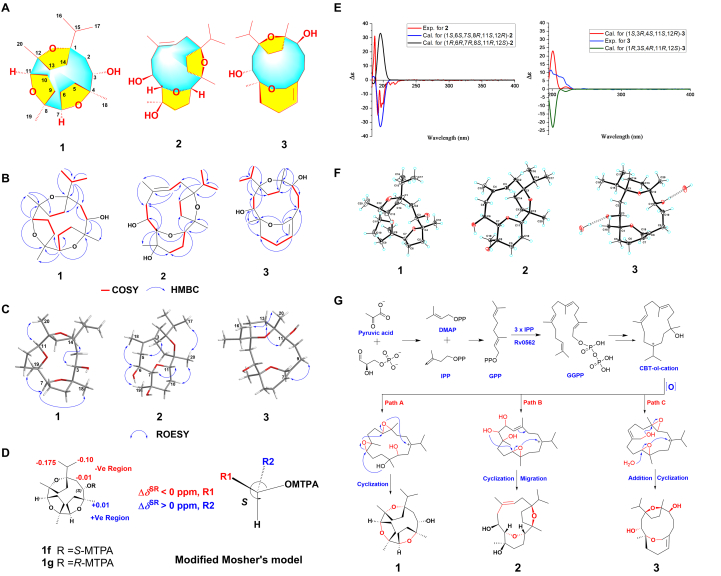
Characterization of compounds **1−3**. (A) Structures of **1**−**3**; (B) Key COSY and HMBC correlations of **1**−**3**; (C) Key ROESY correlations of **1**−**3**; (D) The modified Mosher’s methods analysis of **1 (***Δδ*_*SR*_ [*Δ*(*δ*_*S*_ − *δ*_*R*_)] values of (*S*)- and (*R*)-MTPA esters); (E) The calculated and experimental ECD spectra of **2** and **3** at the B3LYP/6-311g (d,p) level; (F) Perspective ORTEP drawing of the X-ray structures of **1**−**3**; (G) The plausible biosynthetic pathways of **1**−**3**.

The current study is focusing on investigating the anti-fibrotic potentials of *Boswellia papyrifera* (Burseraceae) resin as part of ongoing research on medicinal plant resins^24^, ^25^, ^26^, ^27^. As a result of this work, we have isolated and characterized three novel macrocyclic cembrane type diterpenoids (**1**–**3**) with unique ring systems (Fig. 1A). All the isolates (**1**–**3**) were evaluated for their anti-renal fibrosis activity *in vitro*, while compound **1** was undertaken both *in vitro* and *in vivo* experiments. We performed quantitative chemical proteomic analyses, and found the key target of compound **1** by ABPP. Further, we evaluated the virtual screening of binding sites, and the binding effect of **1**. Present study will lead to the discovery of potential drugs for anti-fibrotic development and provide a foundation for identifying therapeutic targets for organ fibrosis.

## 2 Results and discussion

### 2.1 Discovery and structure elucidation of compounds **1**‒**3**

Compound **1** was isolated as a colorless crystal with optical rotation ${\left[\alpha \right]}_{D}^{25}$ −73.3 (*c* 0.01, MeOH). The molecular formula was assigned as C_20_H_34_O_4_ by its HRESIMS observed protonated molecular ion peak at *m*/*z* 339.2529 with four degrees of unsaturation. Its ^1^H NMR spectrum in CDCl_3_ (Supporting Information Fig. S1) shows three rotational isomers (1.0:0.6:0.3), further NMR analysis of **1** under different deuterated solvents (MeOD, pyridine-*d*_5_ and DMSO-*d*_6_) (Fig. S1) shows 100% one conformer except in CDCl_3_. We have been studied complete 1D and 2D NMR analysis of **1** in MeOD (Table 1 and Supporting Information Table S1). The ^1^H NMR spectrum displays three methyl singlets (*δ*_H_ 1.25, 1.18, 1.15), two methyl doublets (*δ*_H_ 0.93, 0.90), and three oxymethines (*δ*_H_ 4.04, 3.97, 3.80). The ^13^C NMR, DEPT, and HSQC data of **1** reveal twenty carbon resonances including five methyls, seven methylenes, four methines, and four quaternary carbons. The typical down field oxygenated carbons at *δ*_C_ 90.1, 85.1, 84.9, 84.7, 80.7, and 80.1 indicate that compound **1** is built with three oxygen fused ring systems, accounting for three double bond equivalence (DBE). The remaining one unsaturation from its molecular formula indicates the fact of a macrocyclic ring. The structure assembly was carried out using 2D NMR experiments. The ^1^H–^1^H COSY correlations (Fig. 1B) show five spin systems H-2/H-3; H-5/H-6/H-7; H-9/H-10/H-11; H-13/H-14; H-15/H_3_-16/H_3_-17. The HMBC correlations (Fig. 1B) of H-7/C-4, C-6, C-8, C-9, C-19 and H-11/C-8, C-12, C-20 indicate two oxygen bridges between C-4/C-7 and C-8/C-11 respectively. Moreover, the remaining an oxygen fuse formation between C-1 and C-12 was confirmed by the HMBC correlations of H_3_-20/C-12, C-13, C-11; H_3_-16/C-15, C-1; H-15/C-1, C-2, C-14, in comparing with downfield chemical shifts of C-1 (*δ*_C_ 90.1) and C-12 (*δ*_C_ 80.7). Finally, a free secondary hydroxy group at C-3 was deduced from the correlations of H-3/C-1, C-2, C-4, C-18 and it was confirmed by mono *p*-nitrobenzoic ester derivative. Thus the planar structure of **1** was assigned as trioxatetracyclo[10.2.1.1^2,5^.1^6,9^]heptadecane ring system of cembrane-type skeleton. The relative configuration of **1** was elucidated by the comprehensive analysis of ROESY and QM-NMR calculations. The ROESY correlations (Fig. 1C) of H-7/H_3_-18, H_3_-19; H-11/H_3_-20, and H-3/Hb-14 exhibit three facial regions (H-7, H_3_-18, H_3_-19; H-11, H_3_-20; H-3, H-14). However, these correlations are insufficient to find relative configuration by interlinking of all stereo genic centers, and hence indispensable to further QM-NMR studies of five possible configurations (**1a**‒**1e** in Supporting Information Figs. S2‒S4 and Supporting Information Table S2). Careful analysis of the correlation coefficients (*R*^2^) obtained by linear regression and the DP4+ probability results provided that the **1b** have significantly higher DP4+ probability score (Supporting Information Tables S3 and S4) and hence the relative configuration of **1** was assigned as 1*S*∗,3*S*∗,4*R*∗,7*S*∗,8*R*∗,11*S*∗,12*R*∗.

**Table 1:** ^1^H (500 MHz) and ^13^C (125 MHz) NMR data of **1**‒**3** (*δ*_H_ in ppm, *J* in Hz).

Position	1^a^		2^b^		3^b^	
	*δ*_H_	*δ*_C_	*δ*_H_	*δ*_C_	*δ*_H_	*δ*_C_
1		90.1, s		90.0, s		88.6, s
2	Ha: 1.39, dd (15.0, 10.0)	37.8, t	Ha: 2.01, dd (13.0, 11.0)	28.1, t	Ha: 1.44, dd (15.5, 8.2)	36.0, t
	Hb: 2.39, d (15.0)		Hb: 2.17, dd (13.0, 5.5)		Hb: 2.15, overlap	
3	3.97, d (9.1)	70.2, d	5.40, dd (11.0, 4.7)	125.8, d	4.37, d (8.3)	69.9, d
4		85.1, s		130.4, s		80.0, s
5	Ha: 1.49, m	36.7, t	Ha: 2.24, d (12.5)	51.7, t	Ha: 1.86, m	30.2, t
	Hb: 2.23, m		Hb: 2.61, dd (12.5, 10.5)		Hb: 2.33, m	
6	Ha: 1.54, m	27.9, t	3.76, m	69.9, d	Ha: 2.36, m	26.3, t
	Hb: 1.96, overlap				Hb: 2.50, m	
7	3.80, dd (9.5, 6.5)	80.1, d	3.16, d (8.7)	87.3, d	5.52, s	130.0, d
8		84.7, s		70.8, s		139.3, s
9	Ha: 1.62, m	33.1, t	Ha: 1.66, m	39.6, t	Ha: 1.91, overlap	34.6, t
	Hb: 2.26, m		Hb: 1.81, m		Hb: 2.21, m	
10	1.97, overlap	26.6, t	Ha: 1.24, m	26.0, t	Ha: 1.58, m	38.0, t
			Hb: 1.53, m		Hb: 1.90, overlap	
11	4.04, t (8.5)	84.9, d	3.30, dd (11.8, 2.2)	85.7, d	3.89, d (9.5)	76.4, d
12		80.7, s		85.3, s		84.6, s
13	Ha: 1.66, m	33.5, t	Ha: 1.51, m	31.9, t	Ha: 1.76, m	36.1, t
	Hb: 1.89, ddd (15.0, 5.0)		Hb: 1.90, m		Hb: 2.06, m	
14	Ha: 1.75, m	30.1, t	Ha: 1.60, m	32.4, t	Ha: 1.40, overlap	29.8, t
	Hb: 2.35, m		Hb: 1.93, m		Hb: 1.96, m	
15	1.69, m	40.2, d	1.74, m	36.3, d	2.18, overlap	32.1, d
16	0.90, d (6.8)	16.6, q	0.87, d (6.8)	19.1, q	0.90, d (6.9)	18.9, q
17	0.93, d (6.8)	16.8, q	1.00, d (6.8)	16.4, q	0.95, d (6.7)	16.7, q
18	1.18, s	17.8, q	1.65, s	17.0, q	1.06, s	23.0, q
19	1.15, s	25.5, q	1.31, s	21.0, q	Ha: 3.56, dd (13.3, 1.2)	66.5, t
					Hb: 4.94, d (13.3)	
20	1.25, s	25.1, q	1.11, s	27.1, q	1.04, s	19.2, q

a Data recorded in CD_3_OD.

b Data recorded in CDCl_3_.

The absolute configuration of **1** at C-3 was assigned by modified Mosher’s method^28^^,^^29^, carried out with *R*- *& S*-MTPACl in NMR tube under pyridine-*d*_5_ to produce *S*- & *R*-MTPA esters. The ^1^H NMR chemical shift differences [*Δδ*_H_ (*δ*_H_-(*S*)-ester–*δ*_H_-(*R*)-ester)] were taken as shown (Fig. 1D) and applied modified Mosher’s rule to confirm the absolute stereochemistry at C-3 as 3*S*. Finally, the qualified single crystal of **1** was performed for X-ray diffraction analysis under Cu K*α* radiation source (*λ* = 1.54184 Å) to determine the absolute stereochemistry as 1*S*,3*S*,4*R*,7*S*,8*R*,11*S*,12*R* with a Flack parameter −0.07 (6) (Fig. 1F and Supporting Information Fig. S5; Supporting Information Table S5)^30^.

We have overcome the confusion of assuming **1** is a mixture of three compounds by using NMR analysis under different solvent conditions. Initially, we could not confirm the purity of the compound by HPLC due to a lack of UV absorbance (without even one double bond). After preparing the *p*-nitrobenzoic ester derivative, we confirmed that compound **1** is optically pure by analyzing it with chiral HPLC (Supporting Information Fig. S6, *t*_R_ = 13.8 min) and named as papyifuran A.

Compound **2** was procured as a colorless crystalline needle showing optical rotation ${\left[\alpha \right]}_{D}^{25}$ −140.0 (*c* 0.01, MeOH). Its molecular formula was found to be C_20_H_34_O_4_ from its HRESIMS experiment (*m*/*z* 339.2530 [M + H]^+^, Calcd. for 339.2535), implying four degrees of unsaturation. The ^1^H NMR spectrum of **2** exhibits five methyls [*δ*_H_ 0.87 (d), 1.00 (d), 1.11 (s), 1.31 (s), 1.65 (s)], three oxymethines [*δ*_H_ 3.16 (d), 3.30 (dd), 3.76 (m)], and an olefinic proton at *δ*_H_ 5.40 (dd). The ^13^C NMR spectrum shows 20 carbon resonances (Table 1), including highly de-shielded oxygenated carbons at *δ*_C_ 90.0, 87.3, 85.7, and 85.3 indicating two oxygen fused ring systems corresponding to two DBE, and an olefin at *δ*_C_ 130.4, and 125.8 accounting for one DBE. The remaining one DBE belongs to the macrocyclic unit same as compound **1**, confirming as a cembrane type macrocyclic diterpenoid core system constructed with two oxygen fused rings and a trisubstituted olefine. For structure construction of **2**, 2D NMR experiments were utilized. The ^1^H–^1^H COSY correlations provide five resonance systems (H-2/H-3; H-5/H-6/H-7; H-9/H-10/H-11; H-13/H-14; H-15/H_3_-16/H_3_-17) (Fig. 1B). The key HMBC correlations (Fig. 1B) of H-7/C-5, C-6, C-8, C-9, C-19; H-11/C-7, C-9, C-12, C-13; H-3/C-2, C-4, C-5, C-18; H_3_-18/C-3, C-4, C-5; H-6/C-7; H-5/H-6 indicate the formation of C-7:C-11 oxygen bridge, a trisubstituted olefin at C-3, and a free hydroxy group at C-6. Further HMBC correlations of H-15/C-1, C-2, C-14, C-16, C-17; H_3_-20/C-11, C-12, C-13; H_3_-16/C-1, C-15; H_3_-17/C-1, C-15 are the evidences of an isopropyl group and H_3_-20 attaching to C-1 and C-12 epoxy bridged head positions. The epoxy bridge between C-1 and C-12 is justified by down field chemical shifts of C-1 (*δ*_C_ 90.0) and C-12 (*δ*_C_ 85.3). Hence the planar structure of **2** was established as a cembrane motif.

The observed ROESY correlations (Fig. 1C) of H-7/H-11, H-6/H_3_-19 and the absence correlations of H-6/H-7, H-7/H_3_-19 indicate that H-7, H-11 are hanging the same face of the ring and H-6, H_3_-19 are opposite face of the ring. Further correlations of H-6/H_3_-18, H-3/Ha-5, Ha-5/H-7 provide firm evidence of *Δ*^3(4)^ olefin having *E*-orientation. Finally, the ROESY correlation of H_3_-17 with H_3_-20 could be explained by the isopropyl group and H_3_-20 taking the same orientation. For the determination of relative configuration of **2**, further quantum chemical NMR calculations varied with H_3_-17, H_3_-20 face (**2a**‒**2b** in Supporting Information Figs. S7‒S9, and Supporting Information Table S6) were carried out to provide compound **2b** having the highest DP4+ probability score (Supporting Information Table S7). By considering ROESY and NMR calculations the relative configuration of **2** was assigned as 1*S*∗,6*S*∗*,*7*S*∗*,*8*R*∗*,*11*S*∗*,*12*R*∗. The absolute configuration of **2** was confirmed as 1*S*,3*E,*6*S,*7*S,*8*R,*11*S,*12*R* by comparing experimental Cotton effects with the calculated ECD spectrum (Fig. 1E)*,* which was further supported by X-ray crystallographic analysis performed under Cu K*α* (*λ* = 1.54184) radiation source with a Flack parameter 0.01(9) (Fig. 1F and Supporting Information Fig. S10; Supporting Information Table S8)^30^. We have confirmed that compound **2** is optically pure by analyzing chiral HPLC (Supporting Information Fig. S11, *t*_R_ = 7.7 min) and named as papyifuran B.

Compound **3** was isolated as crystalline substances, ${\left[\alpha \right]}_{D}^{25}$ −40.0 (*c* 0.01, MeOH). It has the molecular formula C_20_H_34_O_4_ on the basis of the quasimolecular ion at *m*/*z* 339.2533 [M + H]^+^ Calcd. 339.2535 in its HRESIMS, suggesting four degrees of unsaturation. The ^1^H NMR data (Table 1) show four methyl signals [*δ*_H_ 0.90 (d), 0.95 (d), 1.04 (s), 1.06 (s)], two oxygenated methines [*δ*_H_ 3.89 (d), 4.37 (d)], an oxygenated methylene [*δ*_H_ Ha: 3.56 (dd), Hb: 4.94 (d)] and an olefinic proton [*δ*_H_ 5.52 (s)]. Compared to **2**, the preliminary ^13^C NMR and DEPT data of **3** showing the absence of one methyl and forming highly shielded oxygenated methylene (*δ*_C_ 66.5) imply the formation of an oxygen bridge expected through a methyl. The ^1^H–^1^H COSY correlations of **3** (Fig. 1B) reveal five coupled systems with the same pattern as that of **1** and **2** (H-2/H-3; H-5/H-6/H-7; H-9/H-10/H-11; H-13/H-14; H-15/H_3_-16/H_3_-17). To connect these fragments, the HMBC spectrum was evaluated (Fig. 1B), specifically the correlations of H-3/C-1, C-2, C-4, C-18; H_3_-18/C-3, C-4, C-5; H-7/C-6, C-9; H-11/C-9, C-12, C-13, C-20; H-15/C-1, C-2, C-14 were provide firm evidence of a cembrane-type 14-membered ring system with a C-1:C-12 epoxy bridge. In addition, the correlations of H-19/C-4, C-7, C-8, C-9; H-7/C-5, C-6, C-9 confirm the positions of the bridge headed double bond at C-7 and the formation of oxygen fuse through bridge headed C-19 methylene to C-4. Finally, the correlations of H-3 to C-1, C-2, C-4, C-18 and H-11 to C-9, C-10, C-12, C-13, C-20 indicate the two secondary hydroxy positions at C-3 and C-11.

The ROESY correlation (Fig. 1C) of H_3_-16/H_3_-20 indicates that H_3_-20 and the isopropyl group are in same alignment and the absence of correlation between H-3 and H_3_-18 represents opposite to each other. The correlation from H-7 to Ha-9 represents *Δ*^7(8)^ olefin having *Z-*configuration. Rather than these correlations, we cannot observe proper correlations to solve the remaining relative configurations by ROESY experiment. Thus, it is necessary to conduct quantum chemical calculations for the determination of relative configuration. We performed the QM-NMR calculations for five possible configurations (**3a**−**3f** in Supporting Information Figs. S12‒S14, and Supporting Information Table S9), providing that **3b** has 100% DP4+ probability score (Supporting Information Table S10) and hence the relative configuration of **3** was assigned as 1*S*∗,3*R*∗,4*S*∗,11*S*∗,12*R*∗. The absolute configuration of **3** was solved by comparing experimental CD and calculated ECD curves (Fig. 1E), and it was further supported by single crystal X-ray analysis [Fig. 1F and Supporting Information Fig. S15, Supporting Information Table S11, and Flack parameter 0.00(7)]^30^. Finally, the absolute configuration of **3** was unambiguously assigned as 1*S*,3*R,*4*S,*11*S,*12*R.* Compound **3** possesses a dioxatricyclo[9.3.2.1^4,7^]heptadecane carbon core structure constituted with an unusual naturally occurring bridge headed double bond system, and it obeys anti-Bredt’s rule. Finally, **3** was confirmed as optically pure by analyzing it with chiral HPLC (Supporting Information Fig. S16, *t*_R_ = 6.0 min) and named as papyifuran C.

Of note, papyifuran A was obtained as a non-UV absorption substance, it forms without even one double bond, such characteristic property is common in essential oil components. Another interesting factor about papyifuran A is that it exhibits three rotational isomers in CDCl_3_, as observed by NMR analysis. The reason behind this is that the σ-bond rotation of the carbon–carbon (C-1‒C-15) in the isopropyl group or the flexible ring chain (C-1‒C-4) depends not only on the steric interactions of the atoms but also on the driving force of the solvent molecules. Solvent effects influence the conformational preferences of the isopropyl group or macrocyclic aliphatic ring, causing various mechanisms such as polarity, hydrogen bonding, solvation shell formation, and steric hindrance, which may stabilize one conformer over the others. Interestingly, X-ray crystallographic structure analysis of papyifuran A disclosed that it resembles a tortoise physical appearance. The characteristic feature of papyifuran B is an unusual oxygen bridge formed between two secondary bridgehead positions. Another interesting factor in our finding is the terminus of a double bond difficult to form at bridge head position, but papyifuran C is constructed with an abnormal bridgehead double bond with violation of Bredt’s rule and applicable to Fawcett’s rule^31^. Ancestral charophytes are the major source for modern land plants, approximately 500 million years ago, they colonized onto the land and produced terrestrial plants^32^. Reports reviewing marine^33^ and land^34^^,^^35^ cembranoids show that the majority of such structures are from marine corals, narrow in land but exist in resins like *Boswellia* and *Populus* species. These two statements allowed us to conclude that marine and land plant cembranoids might be from the same ancestors which might be microorganisms despite this hypothesis needing confirmation.

All the isolates are constructed with complicated intramolecular ring formations, here in oxidation followed by cyclization’s are the key factors involved in the construction of new ring systems. Based on these observations we have proposed the plausible biosynthetic pathways for these compounds as shown (Fig. 1G). The biosynthesis of compounds **1**–**3** was proposed from pyruvic acid origin followed by initiating cyclization of (*E*,*E*,*E*)-geranylgeranyl pyrophosphate (GGPP) to form monocyclic cembratriene-ol-cation (CBT-ol-cation)^36^. We have proposed three sub paths (Paths A‒C in Fig. 1G) from the key intermediate CBT-ol-cation. In path A, hydroxylation occurs at C-3 followed by epoxidation on C-7 and C-11 olefins, resulting in the form of an oxidized product. Subsequently, an oxygen bridge forms at the cation intermediate after cyclization *via* epoxidation, generating a tri-pentacyclic ring (C-4/C-7, C-8/C-11, C-12/C-1) in compound **1**. By the same way in path B, hydroxylation takes place at C-6, C-7, and C-8, and an epoxide is formed at C-12. Subsequent cyclication is followed by olefin migration from C-4 to C-3, resulting in the formation of a penta- and a hexa-cyclic ether ring containing in compound **2**. In the last path C, oxidation occurs on a key intermediate (CBT-ol-cation), generating two epoxy rings at C-3 and C-11 and a hydroxy group on the methyl at C-8. Further, addition followed by C-1/C-12 ring formation. Simultaneously, a free hydroxy group attacks the C-4 position of another epoxide, resulting in compound **3** *via* the opening of a three membered ether ring and the generation of a six-membered ether ring.

### 2.2 Anti-renal fibrosis activities of compounds (**1**–**3**)

**Figure 2:**
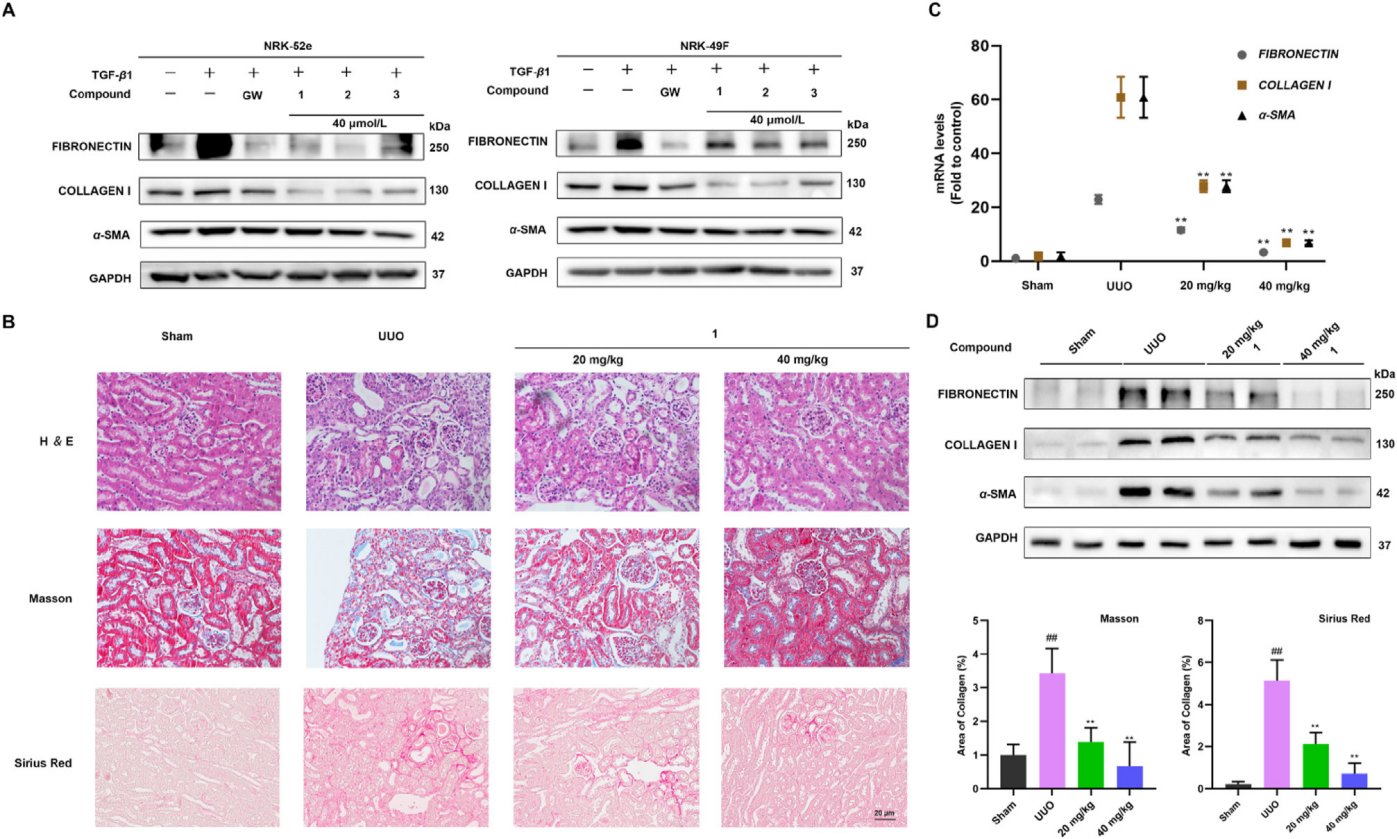
Compound **1** attenuates renal fibrosis *in vitro* and *in vivo*. (A) Representative bands on Western blot showing the expression of fibronectin, collagen I, and *α*-SMA in 10 ng/mL TGF-*β*1-induced NRK-52e and NRK-49F cells (*n* = 3); GW788388 (GW) was used as a positive control which is a TGFBR1 inhibitor; (B) Compound **1** attenuates renal interstitial fibrosis in mice of unilateral ureteral obstruction (UUO) model (scale bar = 20 μm, *n* = 6 mice per group). Mice received daily intraperitoneal injections of vehicle or compound **1** (20 or 40 mg/kg per day) after UUO operation and were euthanized on Day 8. Representative micrographs of hematoxylin and eosin (H*&*E), Masson and Sirius Red’s trichrome staining demonstrating kidney injury in indicated groups; (C) Real-time PCR analyses of mRNA expression of *Fibronectin*, *Collagen I*, and *α-SMA* in the obstructed kidneys; (D) Representative bands (two cases) on Western blot showing the expression of fibronectin, collagen I, and *α*-SMA in the obstructed kidneys. Data are presented as mean ± SEM (*n* = 3). ∗∗*P* < 0.01 compared with UUO alone. ^##^*P* < 0.01 compared with control alone, ns, not significant.

Our group focuses on fibrosis related diseases because almost all chronic diseases are accompanied with fibrosis. With assays against kidney fibrosis in hand, all the isolates in this study were first evaluated using these assays. Initially, the cytotoxicity of these compounds were examined using the CCK-8 assay, and we found no cytotoxicity in rat proximal renal tubular epithelial cells (NRK-52e) and a rat fibroblast cell line (NRK-49F) at a concentration of 40 μmol/L of the three compounds **1**–**3** (Supporting Information Fig. S17). A preliminary screening for nephroprotective activity at a concentration of 40 μmol/L was performed to exclude possible adverse effects. TGF-*β*1 leads to upregulation of tissue repair genes, including DNA repair genes, which further induces the production of fibrosis markers, including *α*-smooth muscle actin (*α*-SMA), collagen I and fibronectin. Thus, we performed a protein blotting-based screen for nephroprotective bioactivity using TGF-*β*1-induced NRK-52e cells and NRK-49F cells, as well as the fibrosis markers fibronectin, collagen I, and *α*-SMA. We found that all three compounds (**1**–**3**) attenuated the expression of fibronectin, collagen I, and *α*-SMA, which are major components of the ECM (Fig. 2A). And the TGF*-β*1-induced expression of fibronectin, collagen I, and *α*-SMA was inhibited in a dose-dependent manner (Supporting Information Fig. S18). In addition, compound **2** showed higher *in vitro* anti-renal fibrosis activity. It possessed higher nephroprotective bioactivity than the other two compounds.

Due to the limited amount of compound **2**, **1** was selected for *in vivo* experiment to assess whether it was worthy of further study. We performed rodent experiments using unilateral ureteral obstruction (UUO) mouse model, which is commonly used in nephrology studies. At the time of examination, UUO mice exhibited severe interstitial inflammation and fibrotic features, and treatment with compound **1** significantly reduced their inflammatory cell infiltration. Compound **1** reduced the inflammatory cell infiltration and improved the tubular morphology which was confirmed by observing in hematoxylin and eosin (H&E), Masson and Sirius Red stained kidney sections (Fig. 2B). Furthermore, the transcriptome analyses and protein blotting showed that the expression of the fibrotic markers fibronectin, collagen I and *α*-SMA was significantly increased in the UUO kidneys as compared to sham-operated controls. Intervention with compound **1** also significantly inhibited the upregulation of *α*-SMA, collagen I and fibronectin in UUO mice at both mRNA and protein levels (Fig. 2C and D). These results suggest that compound **1** could ameliorate renal fibrosis in UUO mice by blocking the upregulation of collagen I, fibronectin and *α*-SMA expression. Taken together, these evidences suggest that compound **1** is a potent anti-renal fibrosis agent both *in vitro* and *in vivo*. The discovery of anti-renal fibrosis activity provides a new structural template for drug development.

### 2.3 EEF2 is a direct target of compound **1**

**Figure 3:**
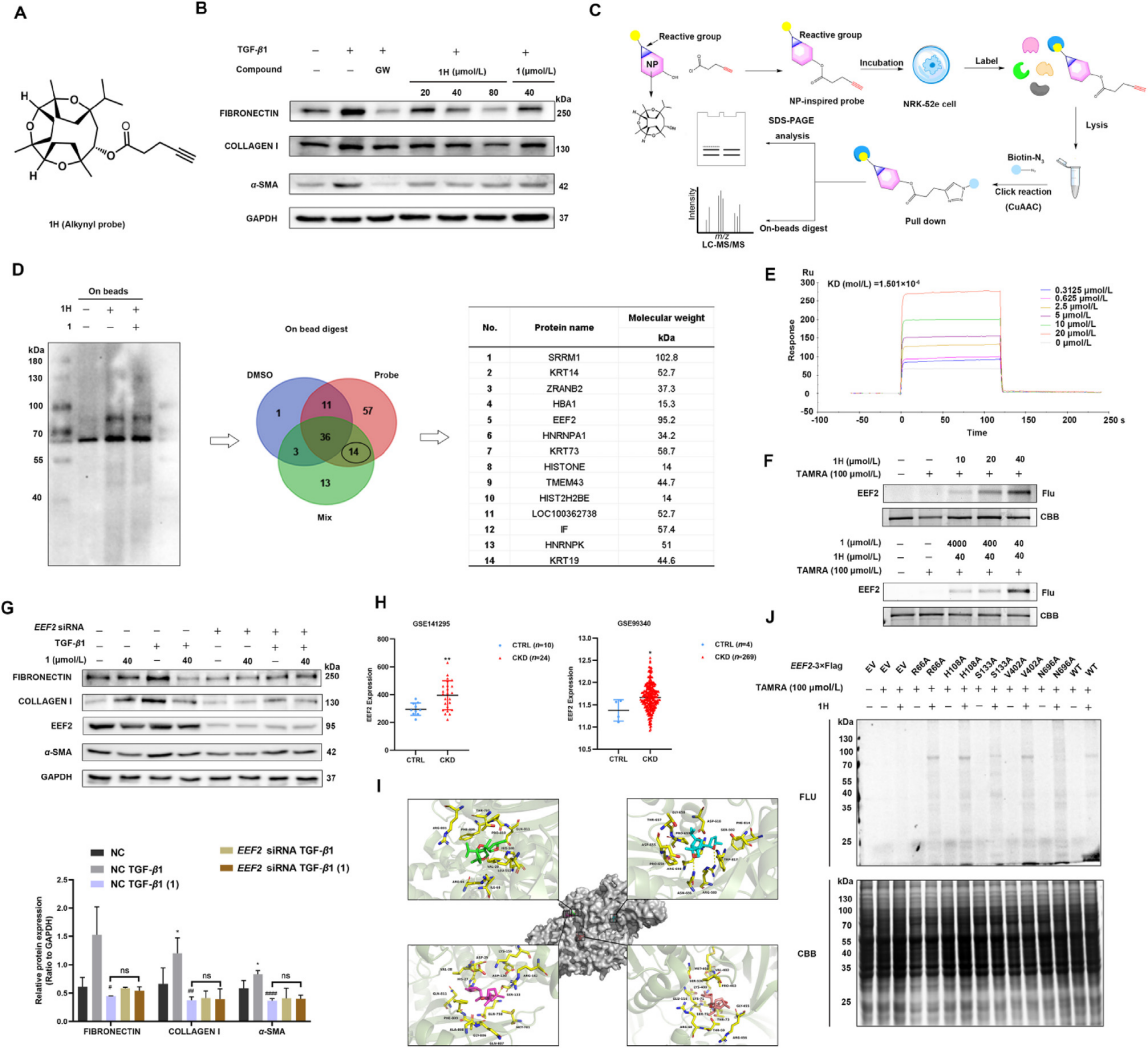
Chemical proteomics approach to identify EEF2 as a target of compound **1.** (A) Chemical structure of **1H**; (B) Determination of the antifibrotic activity of the probe and comparison with that of compound **1***via* Western blot analysis of the expression of fibronectin, collagen I, and *α*-SMA in 10 ng/mL TGF-*β*1-induced NRK-52e cells (*n* = 3); GW788388 (GW) was used as a positive control; (C) Overall scheme for the pull-down experiments to identify compound **1** targets in proteomes; (D) Identification of compound **1** target proteins using pull-down technique coupled with shotgun proteomics. Proteome reactivity profiles of NRK-52e cells treated with **1H** (40 μmol/L) in the presence or absence of compound **1** (400 μmol/L). Whole-cell lysate (WCL) was assayed using pull-down with streptavidin beads. Precipitated proteins were separated using SDS-PAGE; (E) Interaction between compound **1** and recombinant EEF2 protein was analysed by Surface Plasmon Resonance (SPR) assay; (F) **1H** labeling of EEF2 protein in a dose-dependent manner, and **1H** labeling of EEF2 protein in the presence or absence of different concentrations of compound **1**; (G) EEF2 is involved in compound **1**-mediated fibronectin, collagen I, and *α*-SMA suppression inactivation in NRK-52e cells. Cells were transfected with *EEF2* siRNA for 10 h and treated with TGF-*β*1 in the absence or presence of compound **1** for 48 h, followed by determination of the levels of fibronectin, collagen I, and *α*-SMA by Western blot. Data are presented as mean ± SEM (*n* = 3). ∗*P* < 0.05 compared with control alone, ^#^*P* < 0.05, ^##^*P* < 0.01 compared with TGF-*β*1 alone, ns, not significant; (H) Transcription level of *EEF2* in the GEO dataset of CKD clinical samples; (I) Structural model of EEF2–**1** complex based on information from the AlphaFold2 structural library; (J) Fluorescence labeling of EEF2 by **1H**. Flag-tagged EEF2 wild-type (WT) protein and its mutants were expressed in HEK-293T cells then tested for protein binding affinity with Flag beads. Flag–EEF2 protein and its mutants were incubated with **1H** (40 μmol/L) and clickable Cyanine5-N_3_ at 4 °C for 12 h, and the products were resolved by SDS/PAGE and Coomassie blue staining to detect **1H**–labeled EEF2 proteins.

Chemical proteomics-based strategies enabled us to discover functionally unannotated proteins and enable exploratory fishing for protein targets of bioactive small molecules. To gain an insight into the binding protein target of **1**, we first designed and synthesized alkynyl probe (**1H**) to identify the direct targets of compound **1**. In our initial screening of compound **1** analog probes, it was found that the probe exhibited higher anti-fibrotic activity, suggesting that the carboxyl portion would be a suitable binding site for these probes. Therefore, a clickable alkyne label was introduced into the target probe **1H** (Fig. 3A) (Supporting Information Scheme S1). Western blot was performed to detect the anti-fibrotic effect of the probe in TGF-*β*1-induced NRK-52e cells. The results showed that **1H** exhibited no cytotoxicity towards cells (Supporting Information Fig. S19) and significantly down-regulated the expression of *α*-SMA, fibronectin, and collagen I at 40 μmol/L in a dose-dependent manner, suggesting that both were the same in terms of promoting some of the anti-fibrotic activities as compared to the unmodified compound **1** (Fig. 3B). Gel-based ABPP showed that **1H** labeled several protein bands (70–100 kDa) in a dose-dependent manner, which was achieved by a click chemistry reaction coupled to the red fluorescent dye TAMRA-N_3_ (Supporting Information Fig. S20), suggesting the activity of the probe and its ability to bind to target proteins.

Next, we performed pre-partial-based pull-down experiments followed by mass spectrometry-based quantitative proteomics (Fig. 3C). For this purpose, NRK-52e cells were categorized into 3 groups: DMSO group, probe **1H** group (Probe), and mixed group of probe and compound **1** (Mix). After pull-down enrichment, a portion of each sample was subjected to Coomassie Brilliant Blue (CBB) staining and in-gel digestion before LC–MS/MS analysis, while the other portion was subjected to bead trypsin digestion before LC–MS/MS analysis (Fig. 3D, and Supporting Information Fig. S21). The results of CBB staining showed that the main target was the 70–100 kDa protein (Fig. S21), which is consistent with the **1H** in-gel fluorescent labeling results (Fig. S20). The MS data files were analyzed using the MaxQuant program, and protein searches were performed using the Uniprot database. Data analysis of on-bead digest proteomics resulted in the intersection of two replicates, yielding 14 candidate proteins (Fig. 3D), as the specific protein band between 70 and 100 kDa was present in both the probe direct binding group and the competition binding group. However, the prominent band presence at 70 kDa was background protein because the negative control DMSO group also showed this 70 kDa band. Among the 14 candidate target proteins, only the elongation factor 2 (EEF2) protein (95 kDa) have a molecule weight between 70 and 100 kDa. Therefore, EEF2 was prioritized for the selection in the validation experiments. EEF2, a nuclear protein, is mainly expressed in the cytoplasm of eukaryotic cells. It is an elongation factor involved in protein translation that facilitates the movement of ribosomes on mRNA and ensures the insertion of the correct amino acid. EEF2 expression in the cytoplasm is essential for intracellular protein synthesis and quality control, catalyzes the GTP-dependent ribosomal translocation step in the process of translational elongation^37^.

SPR analysis revealed that the target affinity [equilibrium dissociation constant (KD) value] of compound **1** binding to EEF2 was 1.501 μmol/L (Fig. 3E). Next, we conducted *in vitro* direct labeling of recombinant EEF2 protein with **1H** *via* the click reaction with TAMRA-N_3_ and competitive ABPP with unmodified compound **1** *via* in-gel fluorescence scanning (Fig. 3F). Moreover, we found that siRNA-induced suppression of EEF2 expression prevented the downregulation of fibronectin, collagen I and *α*-SMA (Fig. 3G), and reversed the inhibition of **1** on the expression levels of *α*-SMA, fibronectin, and collagen I (Fig. 3G). Moreover, we primarily disclosed the clinical relevance of EEF2 and fibrosis in patients with fibrotic organs by querying the Gene Expression Omnibus of public gene expression profiles^38^^,^^39^, which revealed that the fibrosis patients, including CKD patients, had higher EEF2 transcript levels than healthy donors (Fig. 3H). Therefore, we concluded that EEF2 could be a therapeutic target of compound **1** against renal fibrosis.

### 2.4 Binding site identification for the interaction between compound **1** and EEF2

In order to understand the possible binding modes between **1** and EEF2, we performed AlphaFold2 structure prediction and AutoDock Vina molecular docking studies. AlphaFold2 has become an important solution for predicting the structure of almost all known proteins with a protein prediction rate of 98.5%, and molecular docking calculations are used to search for key interactions between ligands and proteins and find the optimal binding modes using the principles of spatial structure complementarity and energy minimization^40^, ^41^, ^42^. Computational docking methods have been widely used to predict drug binding targets. Possible binding site interactions were evaluated using the predicted EEF2 structure (AF-P05197-F1-model_v4.pdb) from the AlphaFold Protein Structure Database. We first predicted the possible binding pockets of compound **1** towards EEF2 structure and found 17 possible pockets arranged by scores. The top four pockets were selected for molecular docking (Fig. 3I). We ran AutoDock Vina (version 1.1.2 linux) to calculate and finally get the docking results for each pocket based on affinity. Here we show the structure of compound **1** and EEF2 in four different pockets. In Fig. 3I, we showed the residues within a distance of 5 Å from compound **1**, and also showed possible hydrogen bonds predicted by Pymol. These residues that form hydrogen bonds with compound **1** may play important roles in the binding of compound **1** and EEF2: His108 (2.75 Å) in pocket 1, Arg580 (3.40 Å) and Asn696 (2.96 Å) in pocket 2, Ser133(2.91 Å) in pocket 3, Thr59 (3.12 Å) in pocket 4 (Fig. 3I and Supporting Information Table S12), which were predicted to have stronger interactions with EEF2. To validate the results of the molecular docking analysis, we performed a targeted mutagenesis study to confirm that these amino acid sites are associated with the binding of compound **1**. The **1H**-click chemical labeling technique was employed to label five recombinant Flag-tagged mutant EEF2 proteins from HEK-293T cell lysates using TAMRA-N_3_. Interestingly, the H108 mutant showed no difference in probe-labeling intensity (Fig. 3I), although their wild-type (WT) residues were much closer to EEF2 in molecular docking analyses (Table S12). The fluorescence intensities of the probe labeling of the N696 and S133 mutants were much weaker than those of the WT protein (Fig. 3J). In addition, the fluorescence intensity of the probe-labeled N696 mutant was much lower than that of the labeled WT. These results suggested that the interaction of compound **1** with the N696 residue of EEF2 plays an important role in the binding of compound **1** to EEF2. Of course, further confirmation requires protein-molecule co-crystallization, but it is very difficult for EEF2 to obtain its structural information using recombinant proteins. Therefore, co-crystallization experiments have not yet been performed.

### 2.5 Exploring compound-target protein interactions by PROTAC

**Figure 4:**
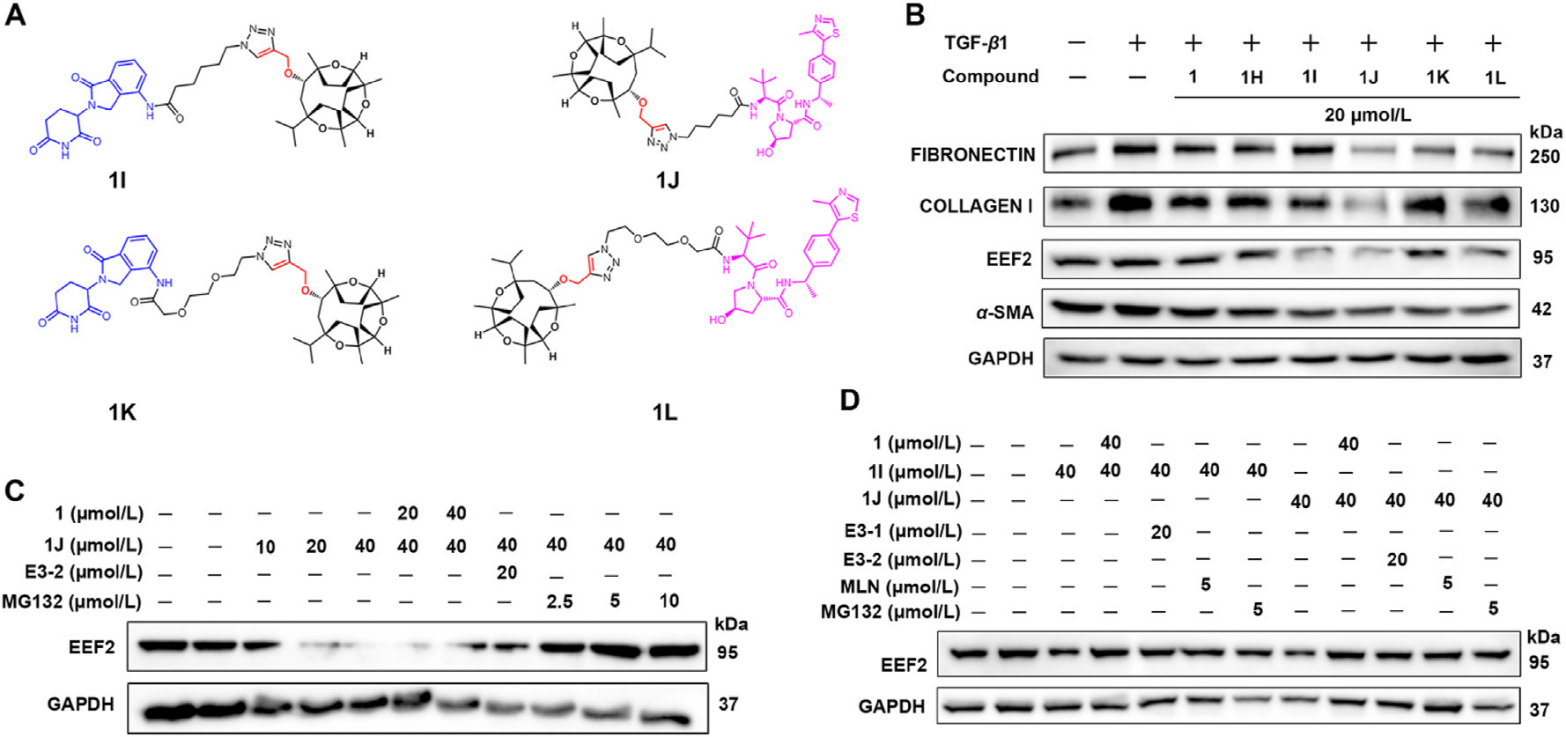
PROTAC validates compounds acting on EEF2 protein. (A) Chemical structures of **1I**, **1J**, **1K**, and **1L**; (B) Determination of the anti-fibrotic activity of the PROTACs and comparison with that of probe and compound **1***via* Western blot analysis of the expression of fibronectin, collagen I, and *α*-SMA in 10 ng/mL TGF-*β*1-induced NRK-52e cells (*n* = 3); (C) NRK-52e cells were treated with E3-2 (20 μmol/L), compound **1** (40 μmol/L)), and MG132 (5 μmol/L)) for 1 h, followed by **1J** (40 μmol/L)) for 12 h. EEF2 protein level was measured by Western blot, and GAPDH protein was used as a loading control; (D) NRK-52e cells were treated with E3-1, E3-2 (20 μmol/L)), compound **1** (40 μmol/L)), MLN4924 (5 μmol/L)), or MG132 (5 μmol/L)) for 1 h and then treated with **1I** and **1J** (40 μmol/L)) for 12 h. The protein level of EEF2 was examined by Western blot. Data are presented as mean ± SEM (*n* = 3).

Despite co-crystals of small molecule and EEF2 was not available, we chose another strategy further to confirm the reliability and utility of targeting EEF2 and to determine its binding mode to small molecules. This strategy is a well-known target-based drug discovery approach, where we did not use a computer-assisted structure optimization method for modification of **1**, in contrast we designed Proteolysis Targeting Chimeras (PROTACs). PROTAC is a method that exploits the naturally occurring intracellular ubiquitin proteasome system (UPS) to validate the binding of the target molecule to the target protein by binding the target protein to E3 ubiquitin ligase and degrading the target protein, thus complete eliminating its function. It consists of three parts: a ligand that binds the target protein, a ligand that binds the E3 ligase, and a connector that connects the two ligands. *In vivo*, the target protein ligand of PROTAC binds to the target protein, and the E3 ubiquitin ligase ligand binds to the substrate-binding region of E3 ubiquitin ligase in the cell, thus “bringing” the target protein closer to E3 ubiquitin ligase through the linker, and thus realizing the degradation of the target protein by the UPS system. Keep this in mind, we applied the above compound **1** to design four PROTACs (**1I**‒**1L**) (Fig. 4A, Synthesis process shown in Supporting Information Scheme S2). Further evaluation of the PROTACs activity on CCK8 assay showed no effect on cell viability (Supporting Information Fig. S22). After that compound **1**, probe and PROTACs were evaluated for their anti-renal fibrosis comparatively, which showed that **1J** has an obvious ubiquitination degradation effect on the target protein (Fig. 4B). Our data in NRK-52e cells demonstrated that **1J** is a very potent EEF2 degradation agent (Fig. 4C), and we further validated the ubiquitinated degradation of PROTAC (**1J**) to further assess the activity and mechanism of action of **1J** in renal cell lines. Further studies found that **1J** also down-regulated fibronectin, collagen I and *α*-SMA expression to a varying degree, with obvious renoprotective effects (Fig. 4B).

Furthermore, we investigated the role of compound **1J** by exploring its mechanism in the degradation of EEF2 protein in the NRK-52e cell line. The results, shown in Fig. 4C, demonstrated that **1J**-induced EEF2 degradation was effective by blocking the degradation of small molecule degradant linked to E3-1 and E3-2 ligases (**1I** and **1J**) by pretreatment with the prototype compound **1**, an antagonist, an activator of the enzyme inhibitor (MLN4924), and a proteasome inhibitor (MG132), in the NRK-52e cell line (Fig. 4D). These mechanistic data clearly indicated that compound **1J** induces EEF2 degradation *via* proteasome and ubiquitination-like-dependent mechanisms and is therefore a bona fide PROTAC EEF2 degradation agent.

### 2.6 Validation of fibrotic pathways treated by EEF2

**Figure 5:**
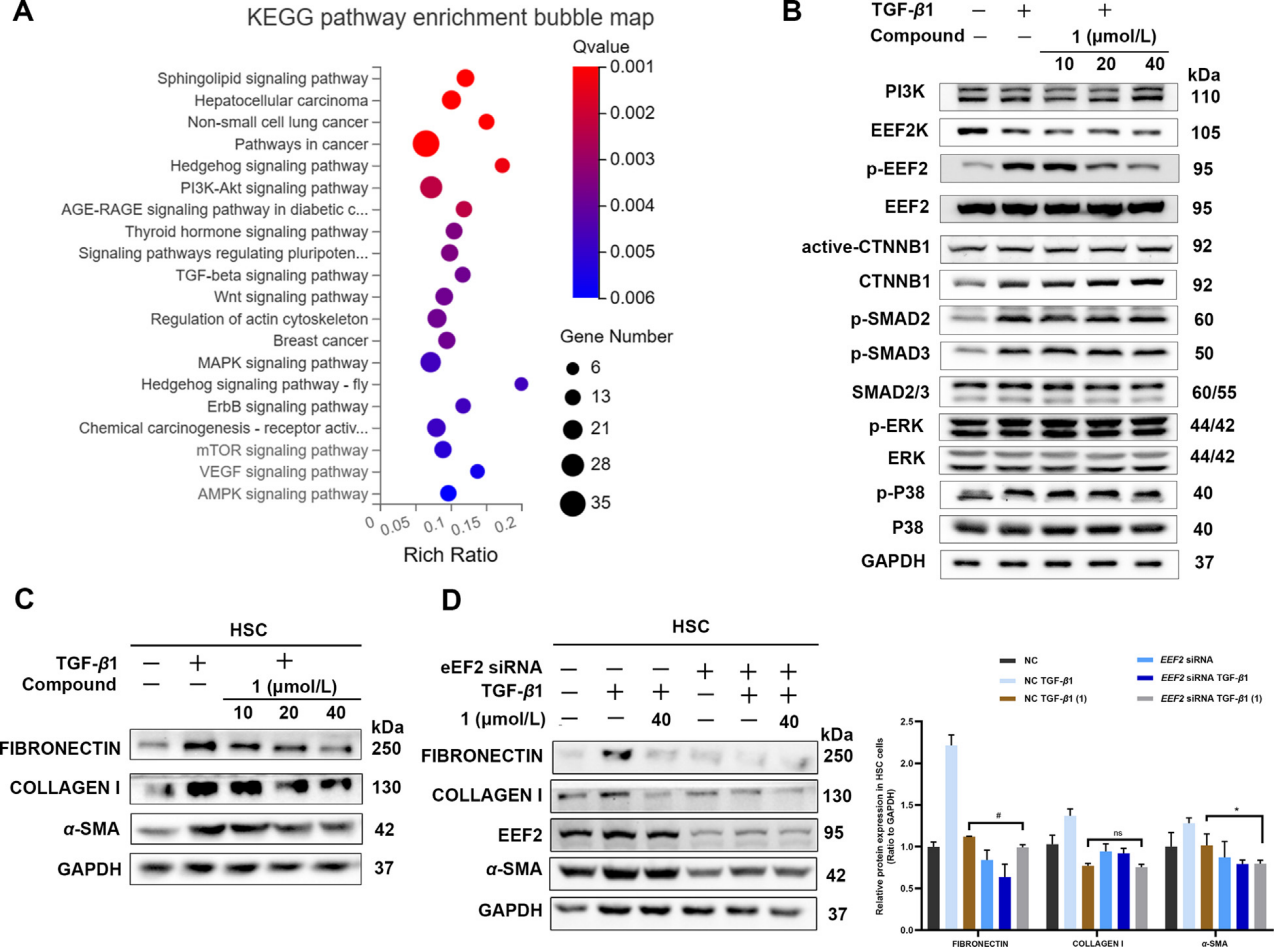
The signaling pathway of EEF2 and roles in organ fibrosis. (A) KEGG enrichment analysis was performed for differentially expressed genes identified by RNA-sequence; (B) Representative bands on Western blot showing the expression of PI3K, WNT/*β*-CATENIN, TGF-*β*/SMAD, P44/42-MAPK (ERK1/2), P38-MAPK signal pathway, EEF2, p-EEF2 and EEF2K with different concentrations of compound **1** in 10 ng/mL TGF-*β*1-induced NRK-52e cells; (C) Representative bands on Western blot showing the expression of fibronectin, collagen I and *α*-SMA with different concentrations of compound **1** in 10 ng/mL TGF-*β*1-induced human hepatic stellate cells (HSC); (D) EEF2 is involved in the inactivation of compound **1**-mediated fibronectin, collagen I and *α*-SMA suppression in 10 ng/mL TGF-*β*1-induced HSC. Cells were transfected with *EEF2* siRNA for 10 h, and then treated with TGF-*β*1 in the absence or presence of compound **1** for 48 h, followed determination of the levels of fibronectin, collagen I, and *α*-SMA by Western blot. Grayscale analysis of protein bands was performed by ImageJ and GraphPad Prism. Data are presented as mean ± SEM (*n* = 3). ∗*P* < 0.05, ^#^*P* < 0.05, ^##^*P* < 0.01. ns, not significant.

To gain an insight into EEF2 mediating renal fibrosis, we conducted a gene expression profiling analysis using RNA sequencing (RNA-seq). Following treatment with si-EEF2, 582 genes exhibited significantly up-regulation, while 669 genes displayed significantly down-regulation compared to the NC cell line (Supporting Information Fig. S23). Among the top 20 pathways identified (Fig. 5A), several signal pathways such as TGF-*β*, WNT, PI3K, and MAPK were down regulated. To examine which signal pathway is mediated by compound **1**, Western blot assays were therefore conducted by examining the key proteins of these signal pathways. The results revealed that the expression of PI3K, CTNNB1, ACTIVE-CTNNB1, p-SMAD2, p-SMAD3, p-ERK (1/2) and p-P38 remained unchanged, suggesting that the antirenal fibrosis effect of compound **1** *via* targeting EEF2 is not likely resulted from these signal pathways or at most EEF2 is the very downstream molecule of the above-mentioned pathways (Fig. 5B). A literature survey disclosed that EEF2 is a crucial elongation factor involved in the peptide chain elongation during translation and EEF2 is a sole known substrate of Eukaryotic elongation factor 2 kinase (EEF2K), which inhibits the elongation step of mRNA translation *via* phosphorylation and inactivation of EEF2^43^, ^44^, ^45^, indicating that targeting EEF2 is equivalent to indirectly regulating EEF2K and EEF2 should be a very downstream molecule in a certain signal pathway. A recent report indicated that EEF2K could inhibit TGF-*β*1-induced NHLF proliferation and differentiation through p38 MAPK signal pathway, which ameliorate lung fibrosis^46^, implying that EEF2 or EEF2K play a pivital role in fibrotic diseases. In general, the role of EEF2 relys on its activation (phosphorylation), we thus examined whether the phosphorylation of EEF2 is influenced when compound **1** binding with EEF2. Fortunately, we found that compound **1** could inhibit EEF2 phosphorylation in a dose-dependant manner (Fig. 5B), indicating that the antifibrotic effect of compound **1** binding with EEF2 may be accompanished by EEF2 phosphorylation. Further efforts on the relation of EEF2 phosphorylation and renal fibrosis need to be explored.

Importantly, we noted that EEF2K is highly overexpressed in various cancers, and EEF2K promotes tumor growth and progression, validating it as a potential molecular target in the development of cancer drugs^47^. It was also reported that EEF2 could regulate cystic fibrosis through promoting ribotoxic stress and subsequent NLRP1 inflammasome activation^48^. In consideration of the role of EEF2K in lung fibrosis^46^ and our current findings, we tentatively concluded that targeting EEF2, a downstream protein of a signal pathway, will be advantageous for antirenal fibrosis drug design and development.

### 2.7 EEF2 is a potent universal drug target for organ fibrosis

To the best of our knowledge, organ fibrosis events in different tissues share some common pathogenic pathway targets. Therefore, we hypothesized that EEF2, identified by compound **1** in renal fibrosis, may have a similar role in fibrosis of other organs. Since EEF2 has been shown to be associated with lung fibrosis^46^ and cystic fibrosis^48^, in previous studies, we further tested the protective effects of the drugs against liver fibrosis *in vitro* using human HSC. The results showed that compound **1** also exhibited a dose-dependent inhibitory effect on the expression of ECM markers in TGF-*β*1-induced fibrotic HSC (Fig. 5C). Importantly, siRNA silencing of the *EEF2* gene also resulted in varying degrees of down-regulation of fibronectin, collagen I and *α*-SMA expression, and reversed the suppression of *α*-SMA, fibronectin and collagen I expression levels by compound **1** (Fig. 5D).

Therefore, we tentatively concluded that EEF2 could be regarded as a broad-spectrum therapeutic target against organ fibrosis. This finding not only expands our understanding of the biological function of EEF2, but also provides an important target for the development of anti-organ fibrosis drugs. Further clinical trials based on EEF2 will require further in-depth studies in order to comprehensively elucidate the mechanism of EEF2 regulation to ameliorate organ fibrosis, including the validation of *in vivo* knockdown of EEF2. However, we only evaluated the anti-organ fibrosis activity of the drug *in vitro*, which is a limitation of this project. Therefore, more comprehensive *in vivo* evaluations are needed in the future to increase the possibility of its clinical application for organ fibrosis treatment. In summary, we identified EEF2 as a potential target for generalized treatment of organ fibrosis, and we found that compound **1** exerts a modulating nephroprotective effect through EEF2. These findings might have important implications for the development of new therapies for the treatment of organ fibrosis either from providing available target or from providing structure template.

## 3 Conclusions

In conclusion, this study revealed three novel cembrane-type macrocyclic oxygen-fused diterpenoids (papyifurans A‒C) from *B*. *papyrifera* exudates. These compounds, specifically compound **1**, exhibit unique scaffolds and have been found to possess a protective effect against renal fibrosis *in vitro* and *in vivo*. The pharmacological target of compound **1** was identified as EEF2 by chemical proteomics technique. Moreover, EEF2 is likely to be a universal fibrotic target of multiple organs evidenced by siRNA interference experiments. Nevertheless, an in-depth insight into the role of EEF2 in multiple organ fibrosis is to be explored.

## 4 Experimental

### 4.1 General experimental procedures

Specific rotations were recorded by Anton Paar MCP-100 digital polarimeter. UV and CD spectra were measured on a Jasco J-815 Circular dichroism spectrometer (JASCO, Japan). NMR spectra were measured on a Bruker AV-600 or an AV-500 spectrometer with TMS as an internal standard. HRESIMS were procured by a Shimadzu LC-20AD AB SCIEX triple TOF X500R MS spectrometer (Shimadzu Corporation, Tokyo, Japan). Column chromatography was undertaken on silica gel (200–300 mesh, Qingdao Marine Chemical Inc., Qingdao, China), MCI gel CHP 20P (75–150 μm, Mitsubishi Chemical Industries, Tokyo, Japan), Sephadex LH-20 (Amersham Pharmacia, Uppsala, Sweden), and YMC gel ODS-A-HG (5–50 μm, 12 nm). The column used for HPLC was YMC-Pack ODS-A 250 mm × 9.4 mm, i.d., 5 μm, Kinetex Biphenyl 100A 250 mm × 9.4 mm, i.d., 5 μm and Daicel Chiralpack IC 250 mm × 4.6 mm, i.d., 5 μm columns.

### 4.2 Plant resins

The resin material of *B. papyifera* (Frankincense) was supplied from Ethiopia in February 2018. The material was identified by Prof. Ermias Dagne at the College of Natural Sciences, Addis Ababa University (Ethiopia) and a voucher specimen (CHYX0613) is deposited at School of Pharmacy, Shenzhen University, China.

### 4.3 Extraction and isolation

The powdered resin of *B. papyifera* (15.0 kg) was soaked with EtOAc (60 L × 4 h × 3). The EtOAc soluble extracts residue (10 kg) was divided into eight fractions (Fr.A−Fr.H) by a silica gel column eluted with gradient petroleum ether-EtOAc (100:1–1:1). Fr.C (2.0 kg) was subjected to an MCI gel CHP 20P column eluted with gradient aqueous MeOH (50%–100%) to afford ten portions (Fr.C.1−Fr.C.10). Fr.C.4 (290.2 g) was subjected to a silica gel column eluted with petroleum ether-EtOAc (9:1–1:1) to produce eight sub-fractions (Fr.C.4.1−Fr.C.4.8). Fr.C.4.4 (64.3 g) was undertaken on a silica gel column by using petroleum ether−Me_2_CO (9.5:0.5–1:1) to generate five fractions (Fr.C.4.4.1−Fr.C.4.4.5). Fr.C.4.4.4 (9.7 g) was further purified by silica gel column chromatography to produce seven fractions (Fr.C.4.4.4.1−Fr.C.4.4.4.7). Fr.C.4.4.4.3 underwent crystallization with MeOH to afford compound **1** (0.546 g).

Fr.C.4.6 (82.0 g) was submitted to a silica gel column eluted with petroleum ether-acetone (5%–50%) to collect several factions pooled as three portions (Fr.C.4.6.1−Fr.C.4.6.3) based on TLC profiling. Fr.C.4.6.1 (10.3 g) was further fractionated by silica gel chromatography washed with gradient solvents of petroleum ether-EtOAc to afford seven fractions (Fr.C.4.6.1.1−Fr.C.4.6.1.7). Fr.C.4.6.1.3 (8.0 g) was purified by silica gel flash column chromatography followed by preparative HPLC carried with aqueous MeOH (70%, 3 mL/min) to obtain compound **3** (18.1 mg, *t*_R_ = 15.4 min, flow rate: 3 mL/min). Further, Fr.C.4.6.3 (18.5 g) was subjected to open ODS column chromatography eluted with gradient aqueous MeOH (30%–100%) to produce eight parts (Fr.C.4.6.3.1−Fr.C.4.6.3.8). Fr.C.4.6.3.6 (1.4 g) underwent silica gel column chromatography eluted with gradient solvents of petroleum ether-acetone to afford six fractions (Fr.C.4.6.3.6.1−Fr.C.4.6.3.6.6). Fr.C.4.6.3.6.6 (127 mg) was purified by preparative HPLC carried with aqueous MeOH (70%, 3 mL/min, biphenyl column) to obtain compound **2** (12.9 mg, *t*_R_ = 38.0 min, flow rate: 3 mL/min).

### 4.4 Compounds characterization data

Papyiferan A (**1**): Colourless crystals (acetone); [*α*]^25^_D_ −73.3 (*c* 0.01, MeOH); HRESIMS (+) *m*/*z* 339.2529 [M + H]^+^ (Calcd. for C_20_H_35_O_4_, 339.2535); ^1^H and ^13^C NMR data, see Table 1; 2D NMR data, see Table S1; HRESIMS and NMR spectra, see Supporting Information Figs. S24−S39.

Papyiferan B (**2**): Colourless crystals (acetone); [*α*]^25^_D_−140.0 (*c* 0.01, MeOH); UV (MeOH) *λ*_max_ (log *ε*) 192 (4.07) nm; CD (MeOH) Δ*ε*_200_ –4.22; HRESIMS (+) *m*/*z* 339.2530 [M + H]^+^ (Calcd. for C_20_H_35_O_4_, 339.2535); ^1^H and ^13^C NMR data, see Table 1; 2D NMR data, see Supporting Information Table S13; HRESIMS and NMR spectra, see Supporting Information Figs. S40−S48.

Papyiferan C (**3**): Colourless crystals (acetone); [*α*]^25^_D_−40.0 (*c* 0.01, MeOH); UV (MeOH) *λ*_max_ (log *ε*) 190 (3.93) nm; CD (MeOH) Δ*ε*_201_ +3.21, and Δ*ε*_201_ +2.53; HRESIMS (+) *m*/*z* 339.2533 [M + H]^+^ (Calcd. for C_20_H_35_O_4_, 339.2535); ^1^H and ^13^C NMR data, see Table 1; 2D NMR data, see Supporting Information Table S14; HRESIMS and NMR spectra, see Supporting Information Figs. S49−S57.

### 4.5 Quantum chemical and electronic circular dichroism calculations

Conformational analysis was conducted using CONFLEX version 7.0, employing the Molecular Merck force field (MMFF) and specific parameters: an energy window of 5 kcal/mol above the ground state, a maximum of 100 conformations per molecule, and an RMSD cutoff of 0.5 Å. Subsequently, selected conformers underwent Gauge-Independent Atomic Orbital (GIAO) calculations to ascertain ^1^H and ^13^C NMR chemical shifts, employing DFT at the mPW1PW91/6-311+G(d,p) level in methanol with the PCM solvent model *via* Gaussian 09 software^49^. Regression analysis of calculated *versus* experimental shifts was performed, and linear correlation coefficients (*R*^2^) were determined for evaluation. DP4^+^ parameters were then calculated after Boltzmann weighing, using an Excel file provided by Ariel M. Sarotti^50^^,^^51^. Percentages for each conformation were presented in tables. Predominant conformers were further optimized at the B3LYP/6-31G(d,p) level using Gaussian 09 software^52^. Finally, optimized conformers of **2** and **3** underwent ECD calculations employing the B3LYP/6–31(d,p) method with PCM (methanol as solvent) in Gaussian 09 software.

### 4.6 X-ray crystallographic data and analysis

Single crystals of compounds **1**–**3** were grown in acetone. A qualified crystal was selected from each compound and evaluated on an XtaLAB AFC12 (RINC): Kappa single diffractometer. The crystal was kept at 100.00(10) K during data collection. Using Olex2^53^, the structure was solved with the SHELXT structure solution program^53^ using Intrinsic Phasing and refined with the SHELXL refinement package^54^ by Least Squares minimization.

### 4.7 Synthesis of probe (**1H**) and PROTAC molecules (**1I‒1L**)

Compound **1** was dissolved in DMF and placed in an ice bath. A catalytic amount of sodium hydride (NaH) was then added to the solution, which was stirred for 30 min. Subsequently, pent-4-ynoyl chloride (1 eq.) was added and the reaction mixture was allowed to react at room temperature overnight (Scheme S1). The probe (**1H**) was purified by liquid–liquid extraction followed by HPLC purification.

By using standard procedure^55^, we connected two E3 ligases (**E31** and **E32**) with two different linkers, 6-azidohexanoic acid (**L1**) and 2-(2-(2-azido ethoxy) ethoxy) acetic acid (**L2**) to generate four molecules (**E3L1**, **E31L2**, **E32L1**, and **E32L2**) (Scheme S2). These four molecules were individually reacted with proporgylated compound **1** under copper iodide (CuI), acetonitrile (overnight) to generate four PROTAC molecules (**1I**‒**1L**; Scheme S2; Supporting Information Figs. S58‒S65).

Compound **1I**. ^1^H NMR (600 MHz, CDCl_3_) *δ*_H_ 9.10 (s), 8.53 (s), 7.75 (d, *J* = 7.9 Hz), 7.64 (d, *J* = 7.4 Hz), 7.44 (t, *J* = 7.7 Hz), 5.07 (dd, *J* = 13.8, 4.9 Hz), 4.34 (m), 4.03 (t, *J* = 7.6 Hz, 1H), 3.83 (dd, *J* = 9.4, 6.1 Hz), 3.78–3.71 (m), 3.48 (s), 2.80–2.68 (m), 2.67–2.60 (m), 2.40 (t, *J* = 7.2 Hz), 2.32–2.23 (m), 2.16–2.08 (m), 2.07–1.93 (m), 1.93–1.79 (m), 1.74 (dt, *J* = 13.3, 7.7 Hz), 1.67–1.56 (m), 1.49 (dd, *J* = 15.3, 7.0 Hz), 1.38–1.34 (m), 1.33 (s), 1.28 (s), 1.24 (s), 1.24–1.21 (m), 1.20 (s), 1.20 (s), 0.88 (d, *J* = 6.6 Hz), 0.85 (d, *J* = 6.8 Hz); ^13^C NMR (150 MHz, CDCl_3_) *δ*_C_ 171.8, 171.7, 170.3, 169.2, 134.1, 133.1, 132.6, 129.1, 126.3, 120.6, 89.0, 84.1, 83.9, 83.8, 83.1, 81.8, 81.7, 65.2, 51.9, 50.8, 46.9, 39.2, 39.1, 36.3, 33.9, 33.9, 31.5, 31.4, 29.9, 29.7, 28.3, 27.6, 27.2, 26.7, 25.9, 24.7, 23.1, 20.0, 18.0, 17.8.

Compound **1J**. ^1^H NMR (600 MHz, CDCl_3_) *δ*_H_ 8.04 (s, 1H), 7.47 (d, *J* = 7.5 Hz), 7.44–7.40 (m), 7.38–7.32 (m), 6.38 (s), 5.12 (s), 4.59 (m), 4.37 (s), 4.08 (dt, *J* = 15.1, 9.4 Hz), 3.86 (dd, *J* = 9.4, 5.9 Hz), 3.84–3.78 (m), 3.65 (d, *J* = 11.1 Hz), 3.51 (s), 2.99 (s), 2.91 (s), 2.70–2.64 (m), 2.55 (m), 2.28–2.20 (m), 2.15 (s), 2.07 (m), 1.91 (m), 1.78 (dq, *J* = 13.4, 7.5 Hz), 1.71–1.59 (m), 1.56–1.48 (m), 1.37–1.30 (m), 1.28 (s), 1.25 (d, *J* = 3.8 Hz), 1.23 (s), 1.07 (s), 0.93 (d, *J* = 6.8 Hz), 0.90 (d, *J* = 6.5 Hz); ^13^C NMR (150 MHz, CDCl_3_) *δ*_C_ 173.3, 172.0, 169.9, 162.6, 143.4, 130.3, 129.8, 126.6, 125.9, 89.1, 84.1, 84.0, 83.7, 83.1, 81.9, 81.8, 70.0, 65.3, 58.6, 57.5, 56.8, 50.8, 48.9, 39.2, 39.1, 37.1, 36.6, 36.0, 35.7, 35.2, 34.0, 33.9, 31.5, 30.3, 29.9, 29.7, 28.3, 27.6, 27.3, 26.7, 26.5, 25.9, 24.7, 22.3, 20.0, 18.1, 17.9.

Compound **1K**. ^1^H NMR (600 MHz, CDCl_3_) *δ*_H_ 8.52 (s), 8.26 (s), 7.76 (d, *J* = 7.5 Hz), 7.75–7.72 (m), 7.50 (t, *J* = 7.7 Hz), 5.27–5.19 (m), 4.75 (s), 4.58–4.48 (m), 4.45 (d, *J* = 4.6 Hz), 4.12 (s), 4.04 (t, *J* = 7.5 Hz), 3.95 (t, *J* = 5.2 Hz), 3.89–3.79 (m), 3.75 (m), 3.67 (dd, *J* = 5.9, 2.8 Hz), 3.49 (s), 2.94–2.87 (m), 2.85 (dd, *J* = 13.3, 5.2 Hz), 2.62 (d, *J* = 15.4 Hz), 2.49–2.40 (m), 2.28–2.14 (m), 2.10 (dt, *J* = 12.3, 8.8 Hz), 2.05–1.95 (m), 1.92–1.80 (m), 1.79–1.70 (m), 1.63 (m), 1.53–1.46 (m), 1.26 (d, *J* = 3.9 Hz), 1.24 (s), 1.22 (s), 1.19 (s), 0.89 (d, *J* = 6.8 Hz), 0.86 (d, *J* = 6.2 Hz); ^13^C NMR (150 MHz, CDCl_3_) *δ*_C_ 171.2, 169.7, 168.9, 167.8, 134.1, 132.8, 131.8, 129.3, 126.0, 121.6, 89.2, 84.1, 84.0, 83.0, 82.0, 82.0, 70.9, 70.5, 70.2, 69.4, 65.0, 52.0, 50.9, 50.2, 46.5, 46.4, 39.1, 38.9, 33.9, 33.8, 31.5, 28.3, 27.5, 27.3, 26.6, 23.3, 19.8, 18.0, 17.7.

Compound **1L**. ^1^H NMR (600 MHz, CDCl_3_) *δ*_H_ 8.97 (s), 7.69 (s), 7.42–7.36 (m), 7.30 (dd, *J* = 8.3, 3.3 Hz), 5.08 (t, *J* = 7.2 Hz), 4.85 (d, *J* = 12.0 Hz), 4.72 (t, *J* = 8.0 Hz), 4.64 (q, *J* = 5.1 Hz), 4.62–4.58 (m), 4.54 (q, *J* = 3.9, 2.7 Hz), 4.52–4.50 (m), 4.08 (dt, *J* = 11.5, 1.8 Hz), 4.05–4.01 (m), 3.95 (s), 3.93–3.88 (m), 3.85 (dd, *J* = 9.4, 6.0 Hz), 3.78 (dd, *J* = 6.7, 2.7 Hz), 3.71–3.68 (m), 3.65 (d, *J* = 3.5 Hz), 3.64 (s), 3.64–3.61 (m), 3.49 (s), 3.05 (s), 2.64 (dd, *J* = 15.6, 2.5 Hz), 2.56 (s), 2.49 (ddd, *J* = 13.0, 7.9, 4.7 Hz), 2.16–2.08 (m), 2.00–1.99 (m), 1.93–1.81 (m), 1.75 (ddd, *J* = 15.0, 8.1, 4.6 Hz), 1.68–1.57 (m), 1.52 (dd, *J* = 15.6, 6.5 Hz), 1.46 (d, *J* = 7.0 Hz), 1.23 (s), 1.21 (s), 1.04 (s), 0.89 (d, *J* = 6.8 Hz), 0.86 (d, *J* = 6.8 Hz); ^13^C NMR (150 MHz, CDCl_3_) *δ*_C_ 171.2, 169.9, 151.6, 143.9, 129.6, 126.7, 92.8, 84.2, 84.1, 83.0, 83.0, 81.9, 71.1, 70.5, 70.4, 70.2, 69.8, 65.0, 58.6, 56.9, 56.9, 50.4, 48.9, 39.1, 38.8, 37.1, 35.5, 33.9, 30.1, 28.3, 27.5, 27.2, 26.6, 26.5, 22.0, 19.9, 18.0, 17.8, 15.0.

### 4.8 Cell culture and cytotoxicity assay

The cell lines of NRK-52e (rat renal proximal tubule cells), NRK-49F (rat renal fibroblastrat cells), HEK-293T (human embryonic kidney cells) and HSC (hepatic stellate cells) were purchased from Wuhan Purcell Life Science and Technology Co., Ltd. China. All cells were cultured with DMEM medium (GIBCO, NY, USA) containing 10% fetal bovine serum (FBS) and 100 U/mL penicillin−streptomycin. Cells were incubated at 37 °C containing 5% CO_2_. Cell viability was analyzed using a cell counting kit (Beyotime, Shanghai, China) and the manufacturer’s protocol. Cells were inoculated into 96-well microtiter plates with 100 μL of medium (Corning, NY, USA) at a density of 1 × 10^4^/well. Cells were treated with different concentrations of compounds and incubated for 48 h. After 48 h of incubation, 10 μL of CCK-8 reagent was added to each well and incubated for 2 h. All experiments were repeated three times. Absorbance at 450 nm was measured using Cytation1 Imaging Reader (BioTek, Vermont, USA), and cell-free wells were used as blank wells. Cell proliferation was expressed by absorbance.

### 4.9 Western blot

Collected cells were lysed using RIPA lysis buffer containing a cocktail of phenylmethane sulfonyl fluoride (PMSF), protease inhibitors and phosphatase inhibitors. Centrifugation was performed at 12,000 rpm (Eppendorf, Centrifuge 5424 R, Hamburg, Germany) for 10 min at 4 °C. Cell supernatants were collected and protein concentrations were determined using a BCA protein assay kit followed by Western blot analysis. Medium amounts of protein extracts (30 μg/well) from each sample were separated by SDS-PAGE and then transferred to polyvinylidene difluoride (PVDF) membranes. Protein-containing membranes were closed with 5% skimmed milk for 1 h and then incubated with the corresponding primary antibodies overnight at 4 °C, followed by incubation with enzyme-conjugated secondary antibodies at room temperature for 1 h. Blots were developed using ECL reagents and then visualized on ChemiDoc™ MP Imaging System (Bio-Rad, CA, USA). Protein bands were analyzed in grayscale using ImageJ and GraphPad Prism. Data are expressed as mean ± standard error of mean (SEM). Primary antibody lines used were anti-Fibronectin (Abcam, Cambridge, UK), anti-Collagen I (Abcam, Cambridge, UK), anti-*α*-SMA (Sigma–Aldrich, St. Louis, USA), anti-GAPDH (Santa Cruz Biotechnology, CA, USA), anti-PI3K (Cell Signaling Technology, CO, USA), anti-EEF2K (Cell Signaling Technology, CO, USA), anti-Phospho-EEF2 (Cell Signaling Technology, CO, USA), anti-EEF2 (Proteintech, Wuhan, China), anti-active-Ctnnb1 (Cell Signaling Technology, CO, USA), anti-Ctnnb1 (Cell Signaling Technology, CO, USA), anti-Phospho-Smad2 (Cell Signaling Technology, CO, USA), anti-Phospho-SMAD3 (Cell Signaling Technology, CO, USA), anti-Smad2/3 (Cell Signaling Technology, CO, USA), anti-Phospho-p44/42 MAPK (Erk1/2) (Cell Signaling Technology, CO, USA), p44/42 MAPK (Cell Signaling Technology, CO, USA), anti-Phospho-P38 (Cell Signaling Technology, CO, USA), anti-P38 (Cell Signaling Technology, CO, USA). Protein bands were analyzed in grayscale using ImageJ (NIH, MD, USA) and GraphPad Prism (GraphPad Software, CA, USA).

### 4.10 Establishment and treatment of unilateral ureteral obstruction (UUO) mouse model

This study was approved by the Animal Ethics and Welfare Committee of Shenzhen University (Batch No.: 202300184). C57BL/6 mice (6−8 weeks old) were purchased and 6 mice in each group were housed in plastic cages for 1 week. The temperature and humidity were maintained at 24−26 °C and 60%, respectively. After WT mice were anesthetized intraperitoneally, the left ureter was exposed and ligated by suture at two points near the renal hilum, and then the ureter was ligated to establish the UUO model. After the incision was closed, the mice were admitted to a standard mouse cage for recovery. Sham-operated control mice underwent open surgery without removing the right ureter.

The UUO model mice were divided into 3 groups (6 mice per group): group 1, treated with intraperitoneal injection of lysate (soybean oil) for 7 days; groups 2 and 3: treated with intraperitoneal injection of different doses of compound **1** (20 and 40 mg/kg) for 7 days. The mice were necropsied on Day 8 after the UUO operation, and the kidney tissue specimens were collected. Some of the tissues were subsequently fixed in 4% formalin and sectioned for H*&*E Masson and Sirius Red staining, and the rest were stored at −80 °C for Western blot and other analyses. Collagen and protein areas were quantified using ImageJ and GraphPad Prism.

### 4.11 Real-time PCR

Total RNA was prepared from kidney tissue using trizol reagent according to the manufacturer’s instructions (Invitrogen, Carlsbad, USA). cDNA was synthesized from 1 mg of RNA using HiScript III RT SuperMixreagent (Vazyme, Nanjing, China) for qPCR according to the manufacturer’s procedure. The sequences of the primer pairs are shown in Supporting Information Table S15. The mRNA levels of each gene after GAPDH correction were calculated by comparing the CT assay.

### 4.12 Morphological analysis

Two-micrometer sections of paraffin-embedded kidney tissue were stained using Masson’s trichrome or hematoxylin and eosin according to the manufacturer’s protocol. Commercial kits from Wuhan Servicebio Technology Co., Ltd., China, were employed for staining.

### 4.13 Pull down

The experimental procedure was similar as described above. After TGF-*β*1 pretreatment of NRK-52e cells for 48 h, the culture medium was removed and the cells were washed with PBS (2 ×). Proteins were extracted using M-PERTM mammalian buffer and protease inhibitor mixture, phenylmethane sulfonyl fluoride (PMSF) and phosphatase inhibitor mixture. Soluble proteins were obtained by centrifugation and their concentration was determined by BCA kit. Equal amounts of lysed proteins (1.5 mg) from different subgroups were incubated with **1H** (20 μmol/L) or DMSO for 1 h at room temperature with gentle shaking, followed by the addition of compound **1** (200 μmol/L) or DMSO, respectively. After incubation for 1 h, the samples were treated with dithiothreitol (DTT) and iodoacetamide-alkylated *tris*-2-carboxyethylphosphine hydrochloride (TECP). The samples were air-dried, dissolved in 0.05% SDS in PBS solution, and centrifuged at room temperature for 10 min. The solution was incubated with 30 μL of streptavidin beads for 4 h at room temperature, and then the streptavidin beads were washed with 5 mL of PBS containing 1% SDS (3 ×) and 0.1% SDS (1 ×), 6 mol/L urea (3 ×), and PBS (2 ×). Tris-(2-carboxyethyl) phosphine hydrochloride and washed with PBST and PBS buffer. Finally, samples were analyzed using LC−MS/MS. For pull-down experiments, binding proteins were detected using a protein blotting assay and the same procedure.

### 4.14 Construction of plasmids and site mutant

The constructed plasmid and site-mutated human EEF2 protein were cloned into a pcDNA3.1 vector containing a 3 × Flag tag sequence (Supporting Information Table S16). The pcDNA3.1-*EEF2* × Flag-Neo was used as a template for site-directed mutagenesis using the Fast Multi-Site Mutagenesis System (Transgen, Beijing, China). These proteins were expressed in HEK-293 T cells.

### 4.15 Transfection of cells

NRK-52e and HSC cells were transfected with either non-targeting control siRNAs or siRNAs targeting *EEF2* using Lipofectamine RNAiMAX (ThermoFisher Scientific, Massachusetts, USA), followed the manufacturer’s specifications (Genepharma, Shanghai, China), for 48 h. HEK-293 T cells were inoculated in 6-well plates and transfected with plasmids at 90% fusion (Supporting Information Table S17). Cells were transfected with Lipofectamin 3000 (ThermoFisher Scientific, MA, USA) according to the manufacturer’s instructions. Complexes were formed with 1 μg of plasmid pcDNA3.1-*EEF2* × Flag-Neo (WZ Biosciences Inc., Jinan, China) WT or mutant plasmid per well in serum-fee DMEM medium (ThermoFisher Scientific, MA, USA). The plasmids were transfected in FBS-added DMEM for 48 h at 37 °C.

### 4.16 Protein expression and analytical labeling in HEK-293 T cells

HEK-293 T cells transformed with pcDNA3.1-*EEF2* × Flag-Neo WT or mutant plasmid were cultured in DMEM medium containing FBS for 48 h, then the medium was removed and the cells were washed (2 ×) with PBS. Proteins were extracted using M-PERTM mammalian buffer and protease inhibitors, PMSF and phosphatase inhibitors. Soluble proteins were generated by centrifugation and their concentration was determined using a BCA kit. The magnetic beads were incubated with 10 μL of anti-flag magnetic beads overnight at 4 °C, and then washed with 1 mL of PBS containing 0.5% Tween 20 (3 ×), followed by PBST and PBS buffer. Finally, the binding of the probe to the protein was detected by *in vitro* fluorescent labeling.

### 4.17 Molecular docking

The structure of EEF2 (AF-P05197-F1-model_v4.pdb) was downloaded from the AlphaFold Protein Structure Database (https://alphafold.ebi.ac.uk/). The structure of compound **1** was drawn using ChemDraw (19.0) (PerkinElmer, MA, USA) and converted to pdb format by Chem3D (19.0) (PerkinElmer). We added all hydrogens to the structures of compound **1** and EEF2 and distributed charges for them. After that, we saved them with pdbqt format. To predict the possible binding pockets, we used P2Rank, a standalone command line program that predicts ligand binding pockets from a protein structure, downloaded from GitHub (https://github.com/rdk/p2rank). The prediction results provided the positions of all residues in the possible binding pockets and also showed the center position. Finally, 17 possible binding pockets were found in EEF2, with the coordinates of the center position for the top four binding pockets being (−5.832, 14.338, −2.4753), (6.3351, 5.5891, −14.4688), (−17.7766, 7.7837, 3.9526), and (6.7169, 2.4883, 14.5841). Grid Box was set based on these coordinates, with a radius of 30 Å. Then we used Autodock Vina (version 1.1.2) to perform molecular docking. The exhaustiveness of the global search was set to 8, the maximum number of binding modes was 9, and the seed of the docking was “2022”. For each pocket, we got 9 results ordered by scores. We used Pymol to visualize the results, showed all residues within a distance of 5 Å from compound **1** and analyzed the possible hydrogen bond between EEF2 and compound **1**.

## Author contributions

Madhu Babu Sura: Writing – review & editing, Writing – original draft, Visualization, Validation, Software, Resources, Methodology, Investigation, Formal analysis, Data curation, Conceptualization. Yeting Zhou: Writing – review & editing, Visualization, Validation, Software, Methodology, Investigation, Formal analysis, Data curation. Jijun Li: Writing – review & editing, Visualization, Software, Data curation. Yongxian Cheng: Writing – review & editing, Writing – original draft, Visualization, Validation, Supervision, Software, Resources, Project administration, Methodology, Investigation, Funding acquisition, Formal analysis, Data curation, Conceptualization.

## Conflicts of interest

The authors have no conflicts of interest to declare.

## Appendix A Supporting information

The following is the Supporting Information to this article:
