# Supplementary material for: Discovery of papyifuran A as an unusual cembrane diterpenoid from Boswellia papyrifera resin reveals EEF2 as a potent new drug target for fibrosis of multiple organs
Source: Acta Pharm Sin B. 2025 Apr 8;15(6):3181–95. doi: 10.1016/j.apsb.2025.04.004 (PMC12254864; doi:10.1016/j.apsb.2025.04.004)
Supplement: Multimedia component 1 [file mmc1.pdf]

Supporting Information

Discovery of papyifuran A as an unusual cembrane diterpenoid from *Boswellia papyrifera* resin reveals EEF2 as a potent new drug target for fibrosis of multiple organs

Madhu Babu Sura[†], Yeting Zhou[†], Jijun Li, Yongxian Cheng*

Guangdong Provincial Key Laboratory of Chinese Medicine Ingredients and Gut Microbiomics, Marshall Laboratory of Biomedical Engineering, Institute for Inheritance-Based Innovation of Chinese Medicine, School of Pharmacy, Shenzhen University Medical School, Shenzhen University, Shenzhen 518060, China

*Corresponding author.

E-mail address: yxcheng@szu.edu.cn (Yongxian Cheng).

[†]These authors made equal contributions to this work

Contents

**Figure S1.**	^{1}H NMR spectra of **1** in a) $CDCl_3$, b) MeOD, c) DMSO-d_6, and d) pyidine-d_5.
**Figure S2.**	B3LYP/6-31G(d,p) optimized lowest energy conformers for **1a**–**1e**.
**Figure S3.**	Regression analysis of experimental versus calculated ^{1}H NMR chemical shifts of **1** at the mPW1PW91-311g(d,p) level.
**Figure S4.**	Regression analysis of experimental versus calculated ^{13}C NMR chemical shifts of **1** at the mPW1PW91-311g(d,p) level.
**Figure S5.**	ORTEP drawing of X-ray structure of **1** (displacement ellipsoids are drawn at the 50% probability level).
**Figure S6.**	The chiral HPLC analysis of *p*-nitrobenzoic ester derivative of **1** by Daicel Chiralpack IC column (250 mm x 4.6 mm; i.d., 5 µm; Condition: *n*-hexane:isopropylalchol 97:3; flow rate: 1 mL/min).
**Figure S7.**	B3LYP/6-31G(d,p) optimized lowest energy conformers for **2a**–**2b**.
**Figure S8.**	Regression analysis of experimental versus calculated ^{1}H NMR chemical shifts of **2** at the mPW1PW91-311g(d,p) level.

**Figure S9.**	Regression analysis of experimental versus calculated ^{13}C NMR chemical shifts of **2** at the mPW1PW91-311g(d,p) level.
**Figure S10.**	ORTEP drawing of X-ray structure of **2** (displacement ellipsoids are drawn at the 50% probability level).
**Figure S11.**	The chiral HPLC analysis of **2** by Daicel Chiralpack IC column (250 mm x 4.6 mm; i.d., 5 µm; Condition: *n*-hexane:ethanol 98:2; flow rate: 1 mL/min).
**Figure S12.**	B3LYP/6-31G(d,p) optimized lowest energy conformers for **3a**–**3f**.
**Figure S13.**	Regression analysis of experimental versus calculated ^{1}H NMR chemical shifts of **3** at the mPW1PW91-311g(d,p) level.
**Figure S14.**	Regression analysis of experimental versus calculated ^{13}C NMR chemical shifts of **3** at the mPW1PW91-311g(d,p) level.
**Figure S15.**	ORTEP drawing of X-ray structure of **3** (displacement ellipsoids are drawn at the 50% probability level).
**Figure S16.**	The chiral HPLC analysis of **3** by Daicel Chiralpack IC column (250 mm x 4.6 mm; i.d., 5 µm; Condition: *n*-hexane:ethanol 98.6:1.4; flow rate: 1 mL/min).
**Figure S17.**	Cytotoxic effects of compounds in NRK-52e and NRK-49F cells.
**Figure S18.**	Compounds dose-dependently inhibit TGF-β1-induced fibrosis hallmarks in NRK-52e and NRK-49F cells.
**Figure S19.**	Cytotoxic effects of compound **1H** in NRK-52e cells.
**Figure S20.**	Strong fluorescence labeling of the probe and CBB results.
**Figure S21.**	Strong biotin labeling of the probes.
**Figure S22.**	Cytotoxic effects of compounds **1I**-**1L** in NRK-52e cells.
**Figure S23.**	The gene after si-*EEF2* treatment indicates up-regulation (left side) and down-regulation (right side).
**Figure S24.**	ESIHRMS of **1**.
**Figure S25.**	^{1}H NMR spectrum of **1** ($CDCl_3$, 500 MHz).
**Figure S26.**	^{1}H NMR spectrum of **1** (pyridine-d_5, 500 MHz).
**Figure S27.**	^{1}H NMR spectrum of **1** (DMSO-d_6, 500 MHz).
**Figure S28.**	^{1}H NMR spectrum of **1** (CD_3OD, 500 MHz).
**Figure S29.**	^{1}H NMR expansion spectrum of **1** (CD_3OD, 500 MHz).

**Figure S30.**	^{13}C and DEPT-135 NMR spectra of **1** (CD_3OD, 125 MHz).
**Figure S31.**	HSQC spectrum of **1** (CD_3OD, 500 MHz).
**Figure S32.**	HMBC spectrum of **1** (CD_3OD, 500 MHz).
**Figure S33.**	HMBC expansion spectrum of **1** (CD_3OD, 500 MHz).
**Figure S34.**	COSY spectrum of **1** (CD_3OD, 500 MHz) .
**Figure S35.**	ROESY spectrum of **1** (CD_3OD, 500 MHz).
**Figure S36.**	^1H NMR spectrum of *p*-nitrobenzoic ester derivative of **1** ($CDCl_3$, 500 MHz).
**Figure S37.**	^{13}C and DEPT-135 NMR spectra of *p*-nitrobenzoic ester derivative of **1** ($CDCl_3$, 125 MHz).
**Figure S38.**	^1H NMR spectrum of *S*-MTPA ester derivative of **1** (pyridine-d_5, 600 MHz).
**Figure S39.**	^1H NMR spectrum of *R*-MTPA ester derivative of **1** (pyridine-d_5, 600 MHz).
**Figure S40.**	ESIHRMS of **2**.
**Figure S41.**	^1H NMR spectrum of **2** ($CDCl_3$, 500 MHz).
**Figure S42.**	^1H NMR expansion spectrum of **2** ($CDCl_3$, 500 MHz).
**Figure S43.**	^{13}C and DEPT-135 NMR spectra of **2** ($CDCl_3$, 125 MHz).
**Figure S44.**	HSQC spectrum of **2** ($CDCl_3$, 500 MHz).
**Figure S45.**	HMBC spectrum of **2** ($CDCl_3$, 500 MHz).
**Figure S46.**	HMBC expansion spectrum of **2** ($CDCl_3$, 500 MHz).
**Figure S47.**	COSY spectrum of **2** ($CDCl_3$, 500 MHz).
**Figure S48.**	ROESY spectrum of **2** ($CDCl_3$, 500 MHz).
**Figure S49.**	ESIHRMS of **3**.
**Figure S50.**	^1H NMR spectrum of **3** ($CDCl_3$, 500 MHz).
**Figure S51.**	^1H NMR expansion spectrum of **3** ($CDCl_3$, 500 MHz).
**Figure S52.**	^{13}C and DEPT-135 NMR spectra of **3** ($CDCl_3$, 125 MHz).
**Figure S53.**	HSQC spectrum of **3** ($CDCl_3$, 500 MHz).
**Figure S54.**	HMBC spectrum of **3** ($CDCl_3$, 500 MHz).
**Figure S55.**	HMBC expansion spectrum of **3** ($CDCl_3$, 500 MHz).
**Figure S56.**	COSY spectrum of **3** ($CDCl_3$, 500 MHz).
**Figure S57.**	ROESY spectrum of **3** ($CDCl_3$, 500 MHz).
**Figure S58.**	^1H NMR spectrum of **1l** ($CDCl_3$, 600 MHz).
**Figure S59.**	^{13}C and DEPT-135 NMR spectra of **1l** ($CDCl_3$, 150 MHz).

**Figure S60.**	^{1}H NMR spectrum of **1J** ($CDCl_3$, 600 MHz).
**Figure S61.**	^{13}C and DEPT-135 NMR spectra of **1J** ($CDCl_3$, 150 MHz).
**Figure S62.**	^{1}H NMR spectrum of **1K** ($CDCl_3$, 600 MHz).
**Figure S63.**	^{13}C and DEPT-135 NMR spectra of **1K** ($CDCl_3$, 150 MHz).
**Figure S64.**	^{1}H NMR spectrum of **1L** ($CDCl_3$, 600 MHz).
**Figure S65.**	^{13}C and DEPT-135 NMR spectra of **1L** ($CDCl_3$, 150 MHz).
**Scheme S1.**	Synthetic route of probe (**1H**).
**Scheme S2.**	Synthetic route of PROTAC molecules (**1I**–**1L**).
**Table S1.**	HMBC and ROESY data of **1** in CD_3OD (δ in ppm, J in Hz)
**Table S2.**	HMBC and ROESY data of **2** in $CDCl_3$ (δ in ppm, J in Hz)
**Table S3.**	HMBC and ROESY data of **3** in $CDCl_3$ (δ in ppm, J in Hz)
**Table S4.**	Primers for real-time PCR
**Table S5.**	siRNA sequence for *EEF2*
**Table S6.**	Primers for *EEF2* mutation constructs
**Table S7.**	Extracted heats and weighting factors of the optimized conformers of **1** at B3LYP/6-31G(d,p) level
**Table S8.**	DP4+ results obtained for compounds **1**, **2** and **3**
**Table S9.**	DP4+ analysis of experimental NMR data of **1** and shielding tensors of **1a**-**1e**
**Table S10.**	Crystal data and structure refinement of **1**
**Table S11.**	Extracted heats and weighting factors of the optimized conformers of **2** at B3LYP/6-31G(d,p) level
**Table S12.**	DP4+ analysis of experimental NMR data of **2** and shielding tensors of **2a**-**2b**
**Table S13.**	Crystal data and structure refinement of **2**
**Table S14.**	Extracted heats and weighting factors of the optimized conformers of **3** at B3LYP/6-31G(d,p) level
**Table S15.**	DP4+ analysis of experimental NMR data of **3** and shielding tensors of **3a**-**3f**
**Table S16.**	Crystal data and structure refinement of **3**
**Table S17.**	Predicted binding sites and mutated amino acids of compounds to EEF2 protein

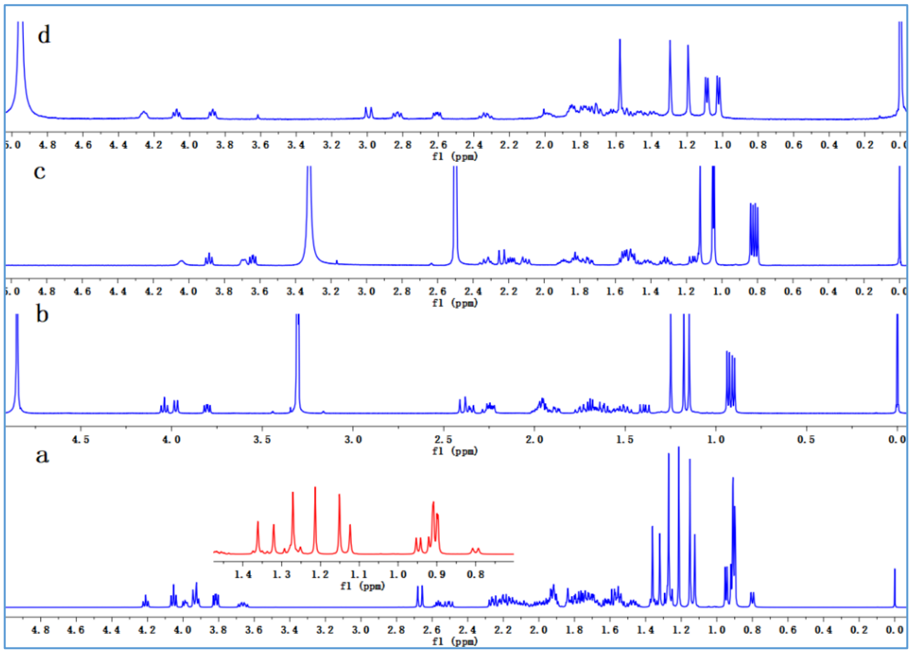

Figure S1. ^{1}H NMR spectra of **1** in a) $CDCl_3$, b) MeOD, c) DMSO-d_6, and d) pyidine-d_5.

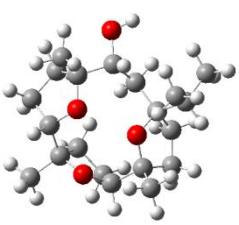 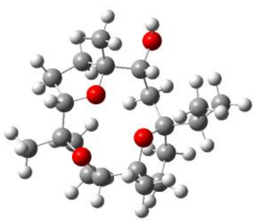 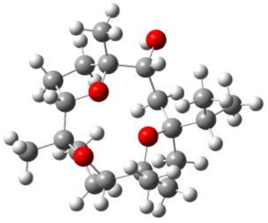 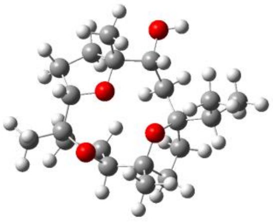

1a-1 **1a-2** **1a-3** **1a-4**

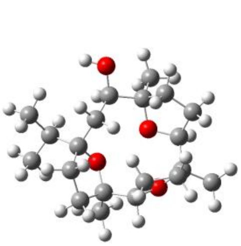 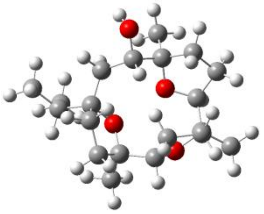 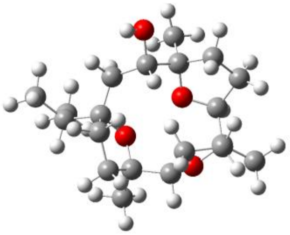 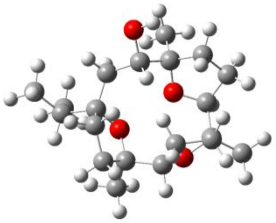

1a-5 **1b-1** **1b-2** **1b-3**

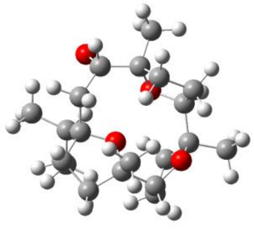 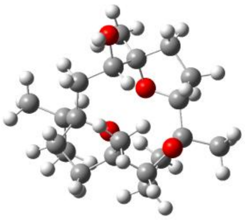 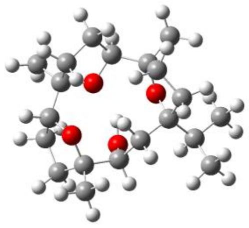 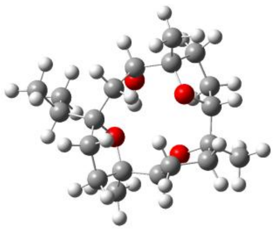

1c-1 **1c-2** **1d-1** **1d-2**

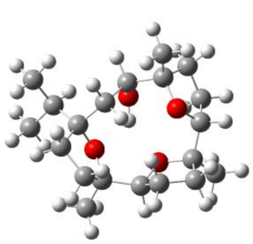
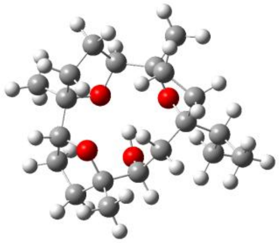
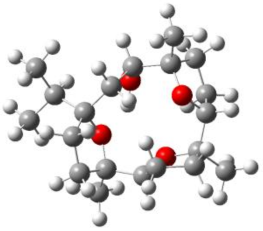
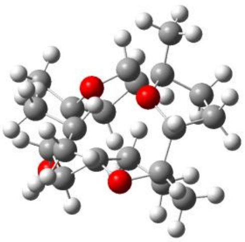

1d-3 **1d-4** **1d-5** **1e-1**

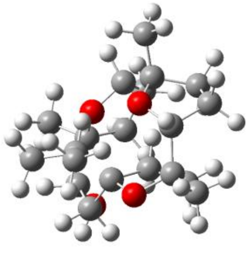
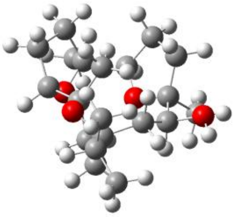
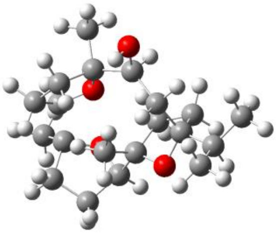
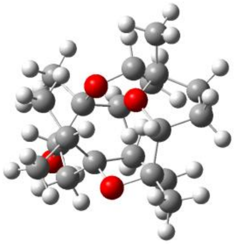

1e-2 **1e-3** **1e-4** **1e-5**

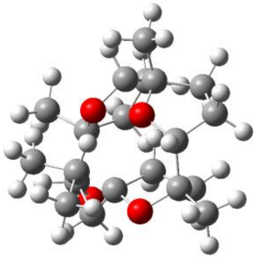
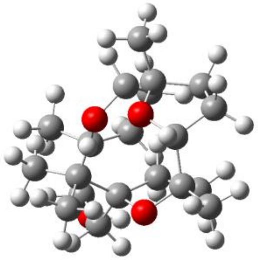
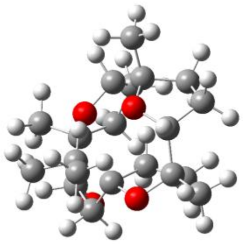
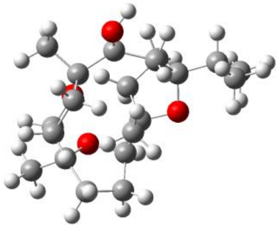

1e-6 **1e-7** **1e-8** **1e-9**

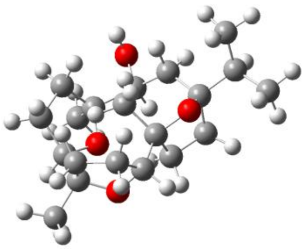

1e-10

Figure S2. B3LYP/6-31G(d,p) optimized lowest energy conformers for **1a–1e**.

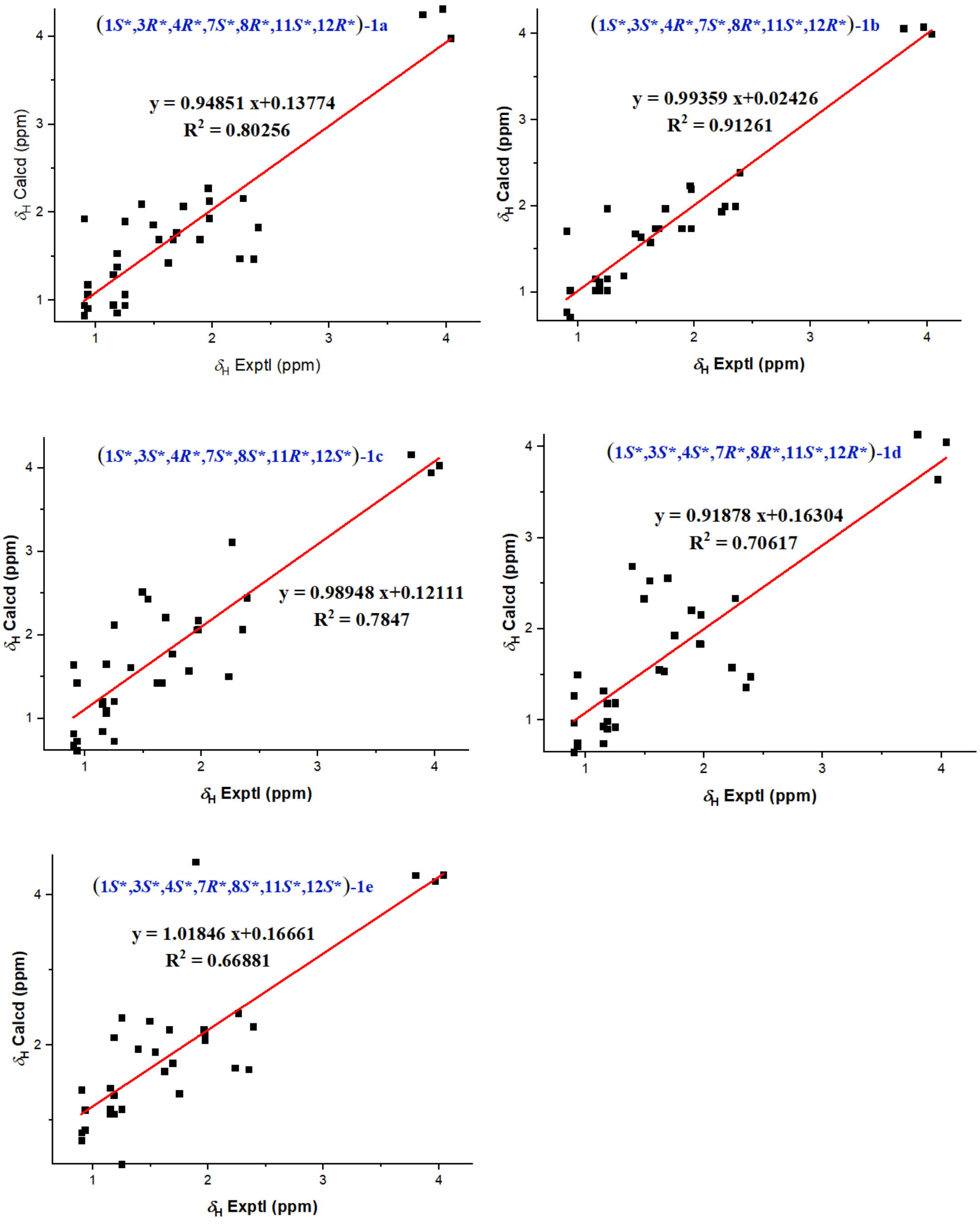

Figure S3. Regression analysis of experimental versus calculated ^{1}H NMR chemical shifts of **1** at the mPW1PW91-311g(d,p) level.

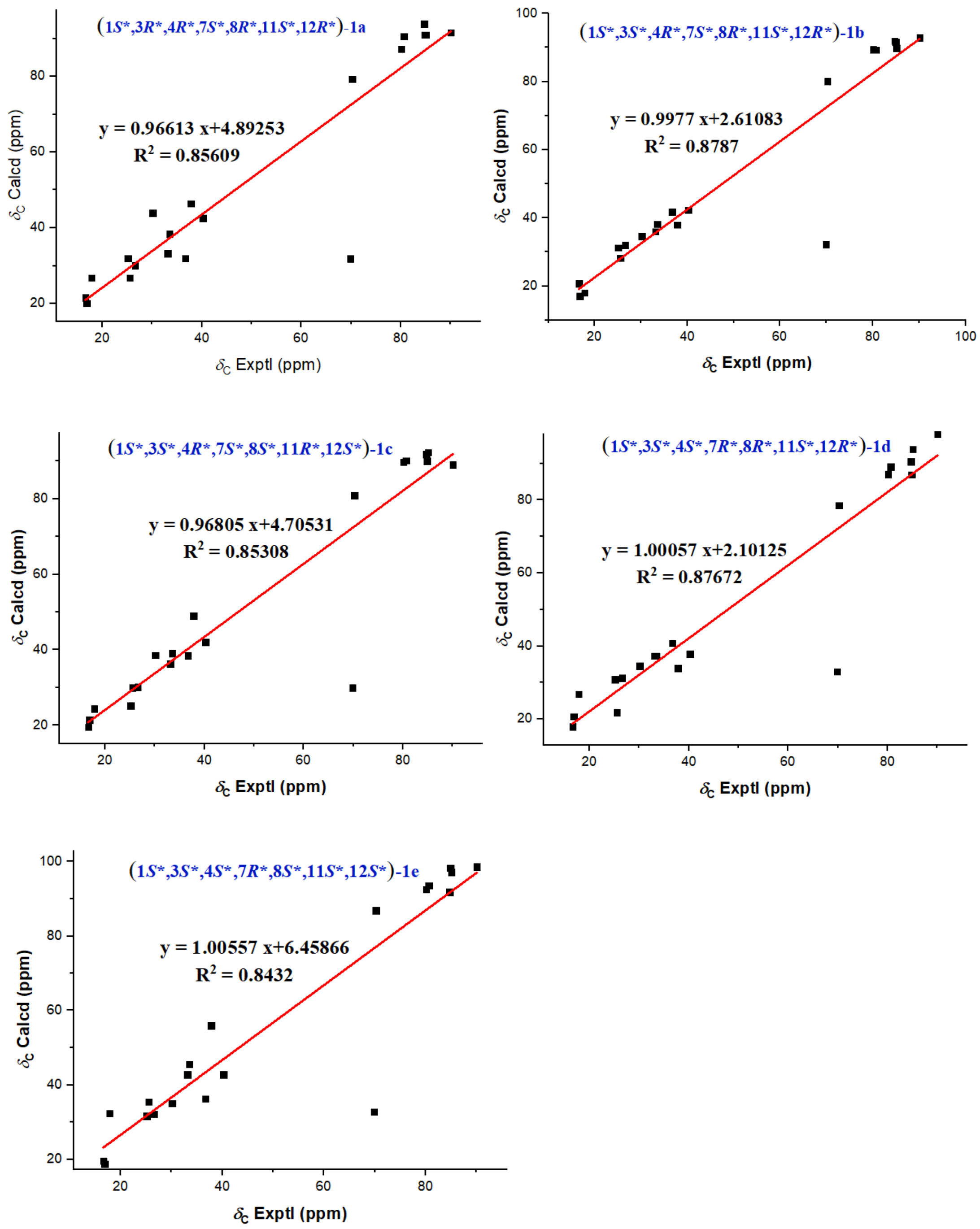

Figure S4. Regression analysis of experimental versus calculated ^{13}C NMR chemical shifts of **1** at the mPW1PW91-311g(d,p) level.

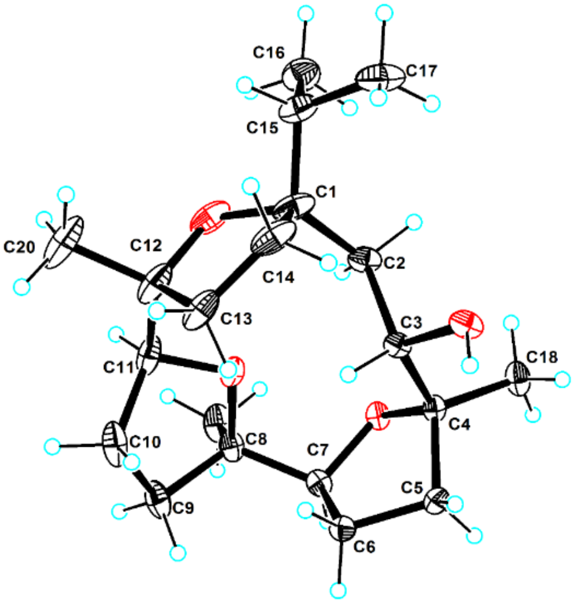

Figure S5. ORTEP drawing of X-ray structure of **1** (displacement ellipsoids are drawn at the 50% probability level).

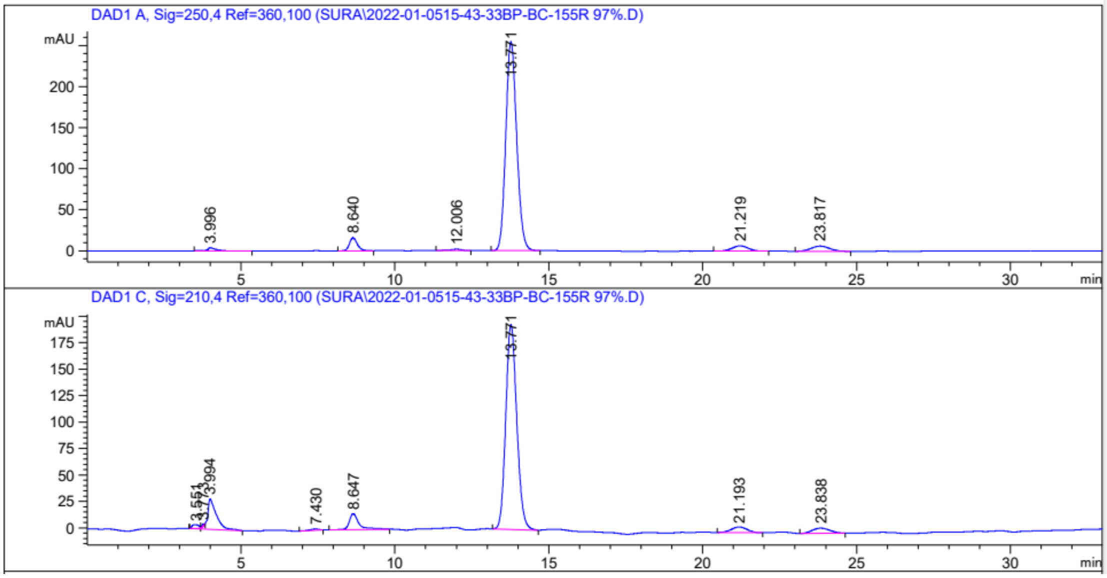

Figure S6. The chiral HPLC analysis of *p*-nitrobenzoic ester derivative of **1** by Daicel Chiralpack IC column (250 mm x 4.6 mm; i.d., 5 μm; Condition: *n*-hexane:isopropylalchol 97:3; flow rate: 1 mL/min).

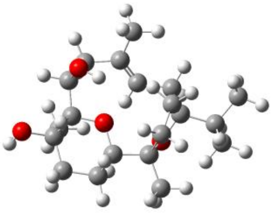
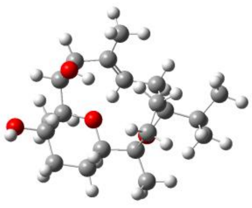
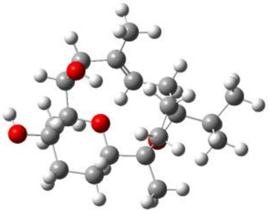
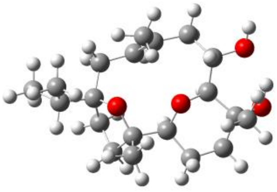

2a-1 **2a-2** **2a-3** **2a-4**

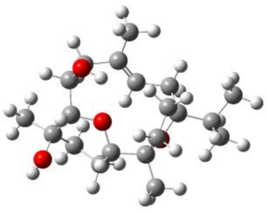
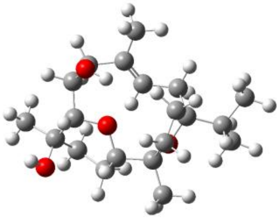
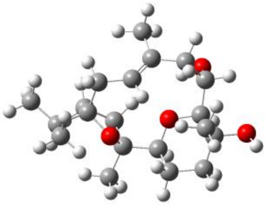
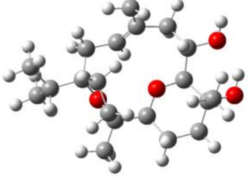

2a-5 **2a-6** **2a-7** **2b-1**

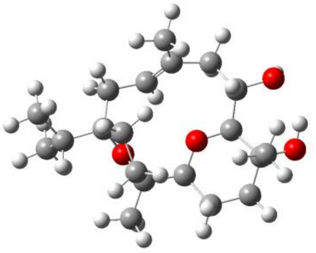
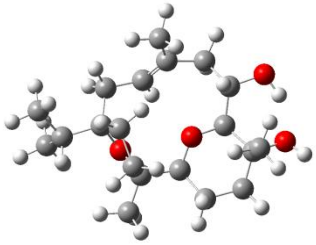
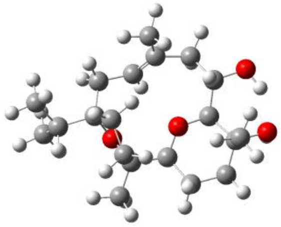

2b-2 **2b-3** **2b-4**

Figure S7. B3LYP/6-31G(d,p) optimized lowest energy conformers for **2a**–**2b**.

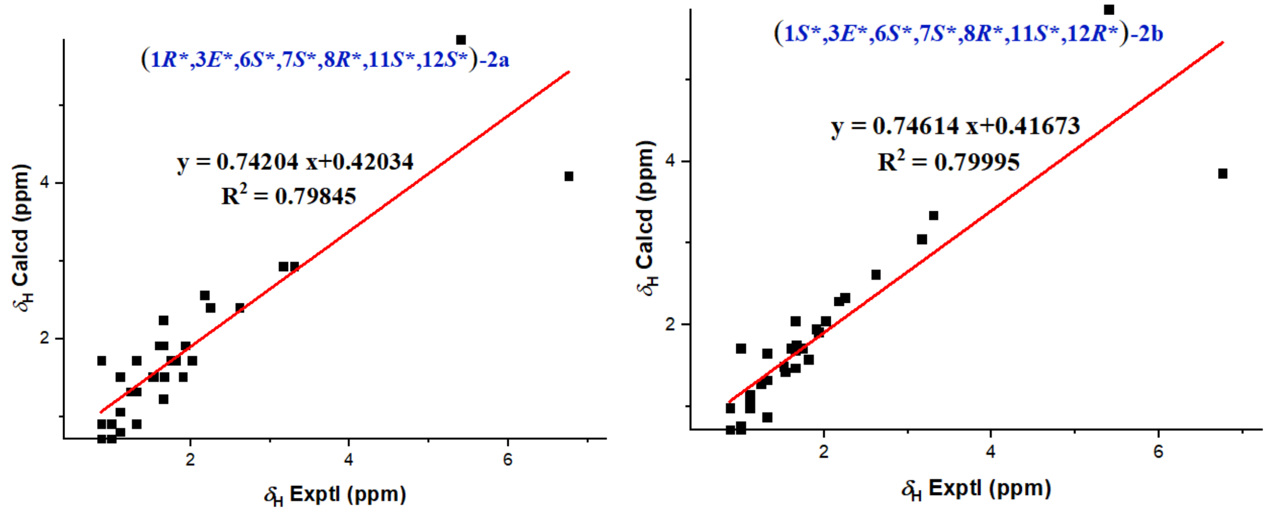

Figure S8. Regression analysis of experimental versus calculated ^{1}H NMR chemical shifts of **2** at the mPW1PW91-311g(d,p) level.

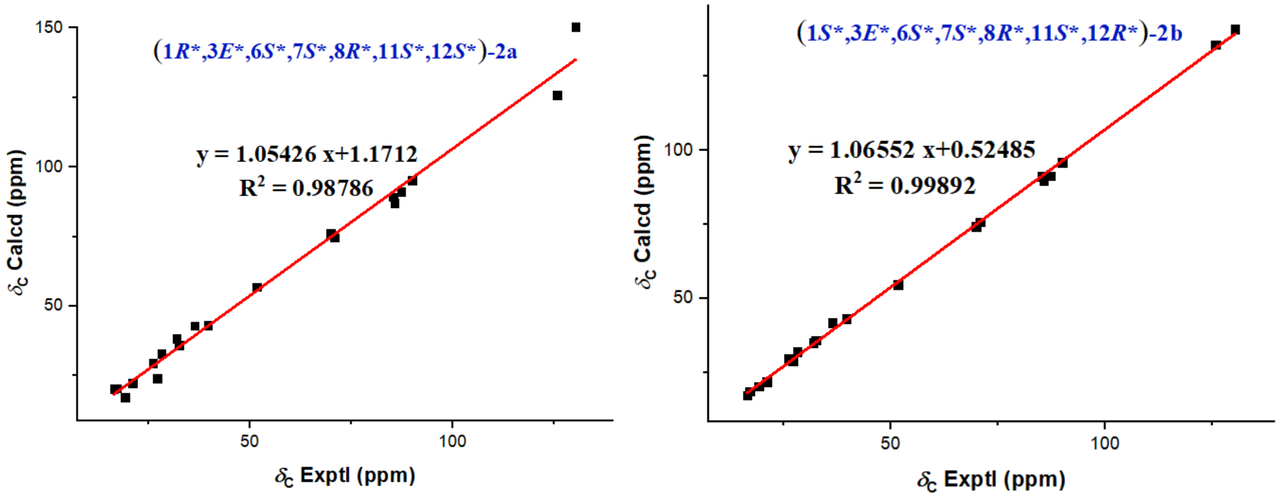

Figure S9. Regression analysis of experimental versus calculated ^{13}C NMR chemical shifts of **2** at the mPW1PW91-311g(d,p) level.

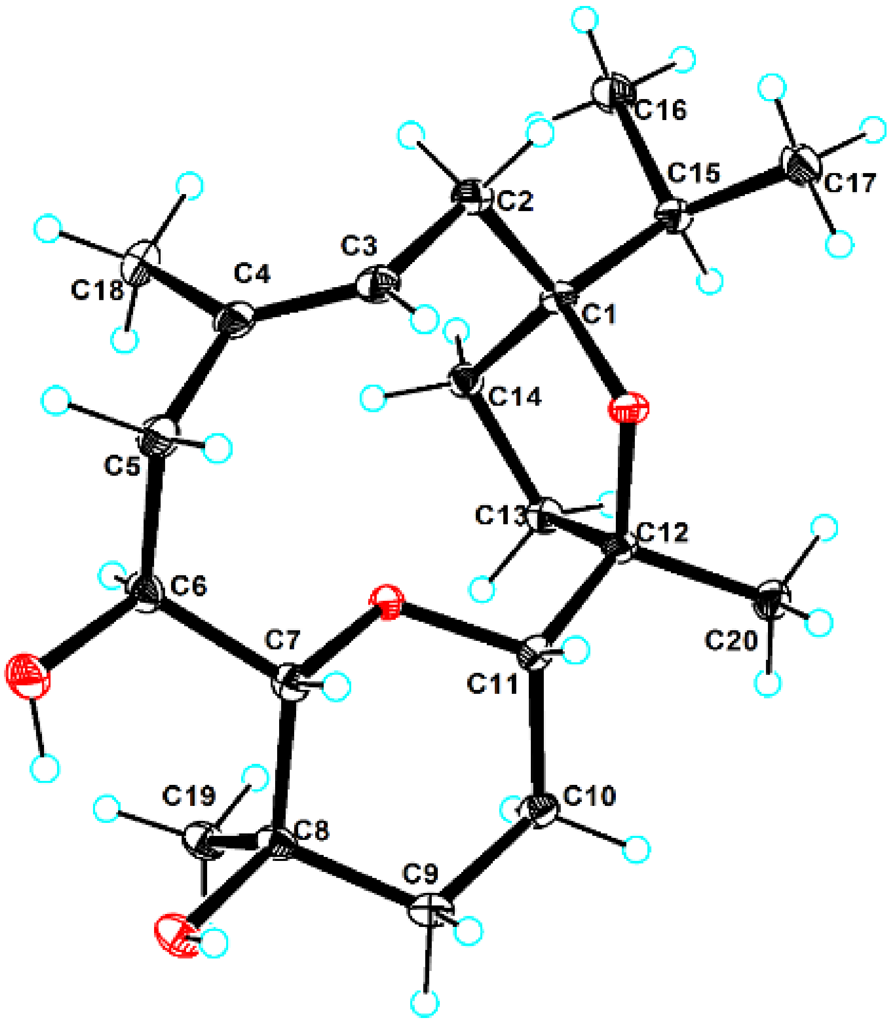

Figure S10. ORTEP drawing of X-ray structure of **2** (displacement ellipsoids are drawn at the 50% probability level).

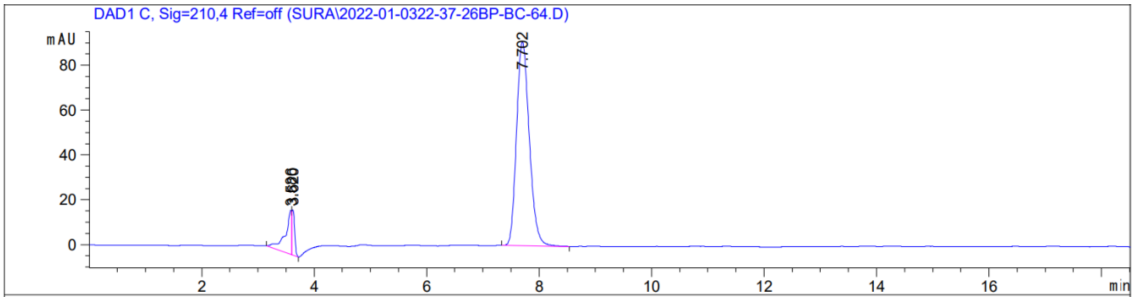

Figure S11. The chiral HPLC analysis of **2** by Daicel Chiralpack IC column (250 mm x 4.6 mm; i.d., 5 μm; Condition: *n*-hexane:ethanol 98:2; flow rate: 1 mL/min).

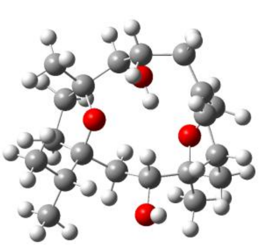
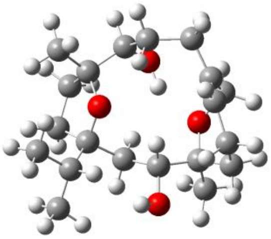
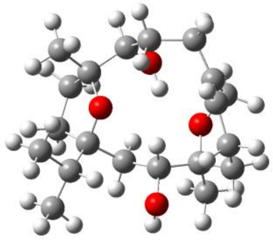
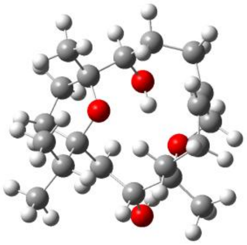

3a-1 **3a-2** **3a-3** **3a-4**

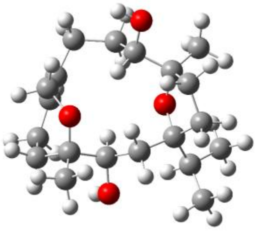
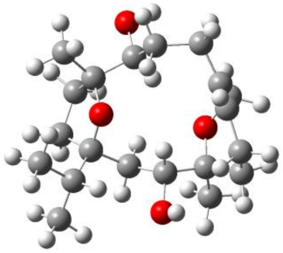
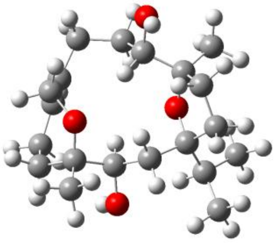
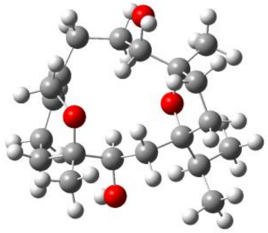

3b-1 **3b-2** **3b-3** **3b-4**

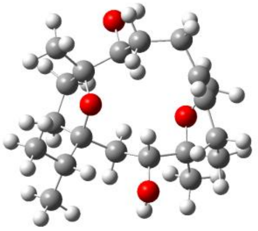
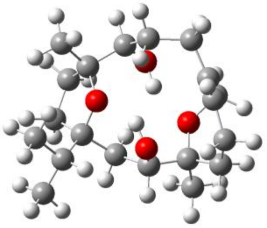
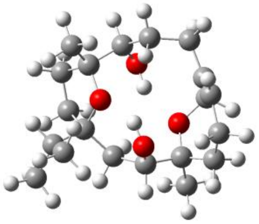
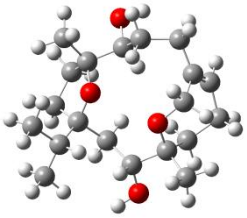

3b-5 **3c-1** **3c-2** **3d-1**

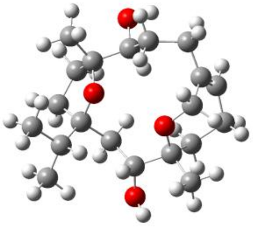
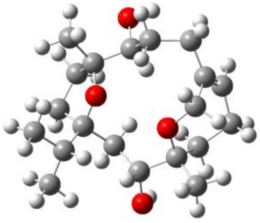
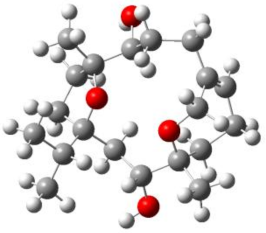
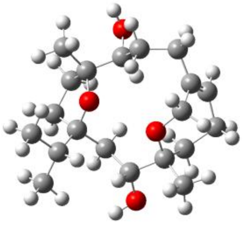

3d-2 **3d-3** **3d-4** **3d-5**

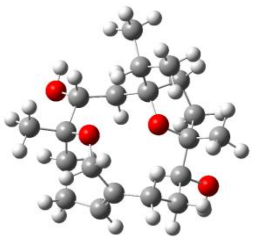
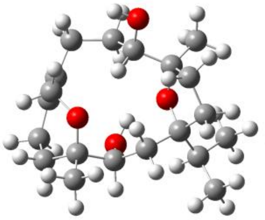
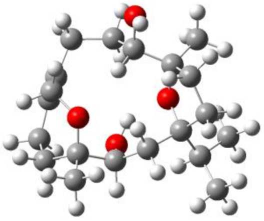
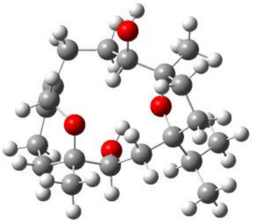

3d-6 **3e-1** **3e-2** **3e-3**

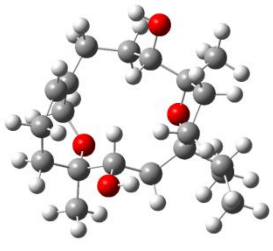

3f-1

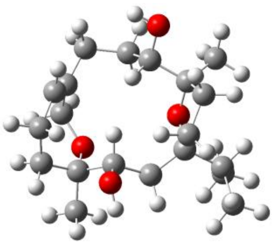

3f-2

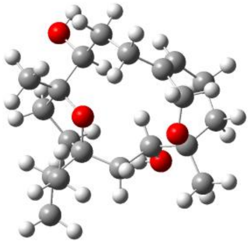

3f-3

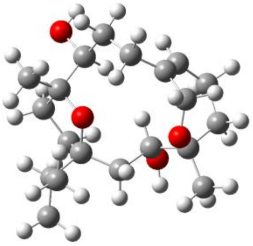

3f-4

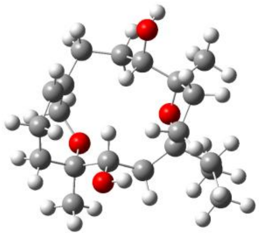

3f-5

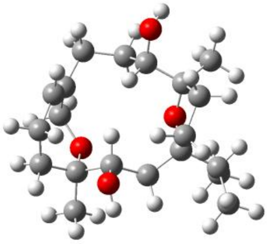

3f-6

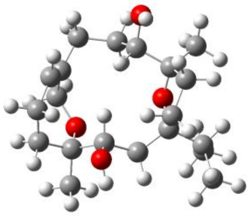

3f-7

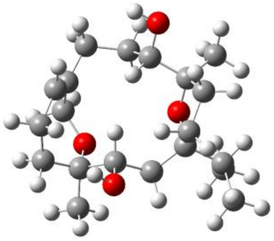

3f-8

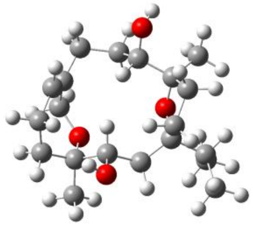

3f-9

Figure S12. B3LYP/6-31G(d,p) optimized lowest energy conformers for **3a–3f**.

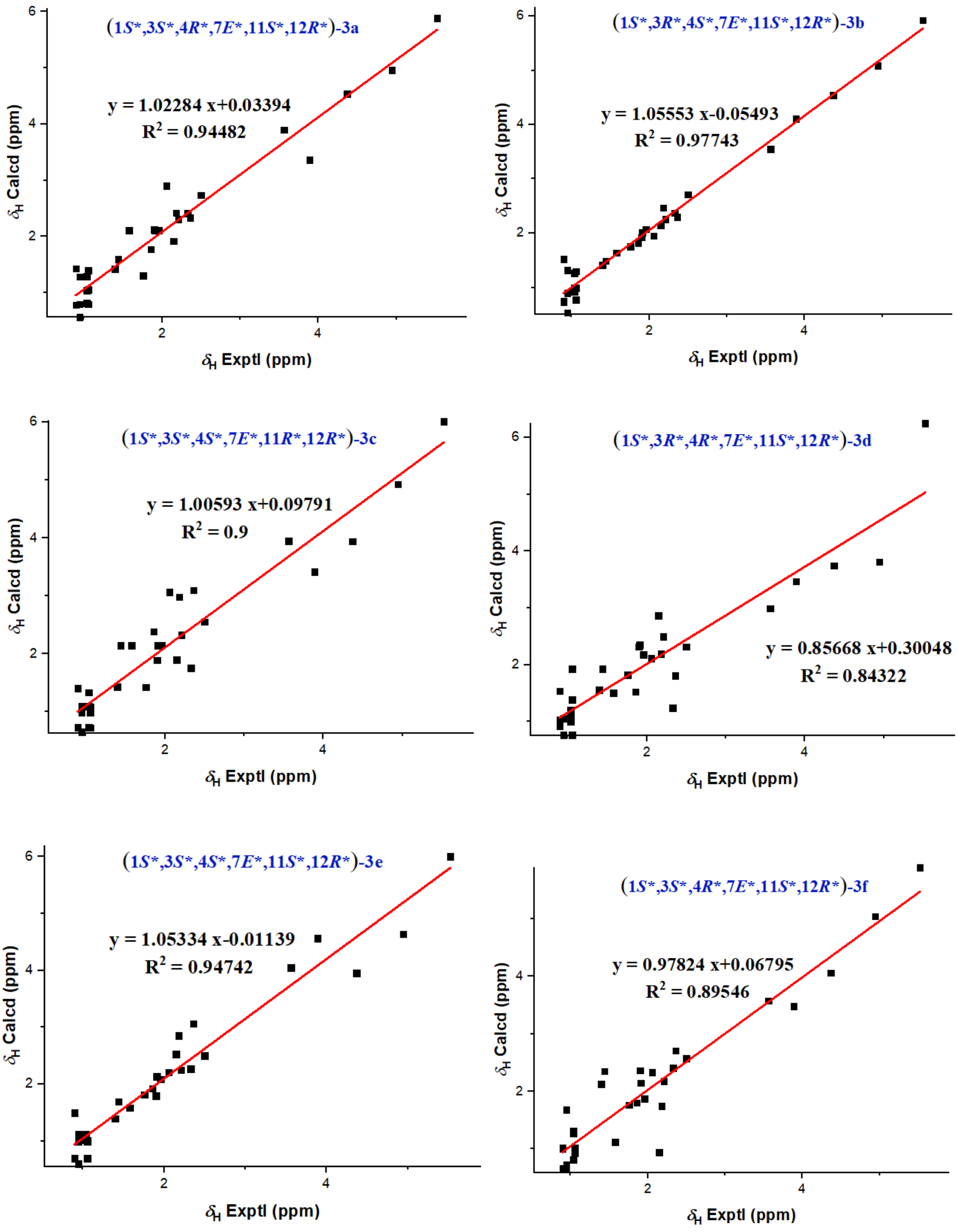

Figure S13. Regression analysis of experimental versus calculated ^{1}H NMR chemical shifts of **3** at the mPW1PW91-311g(d,p) level.

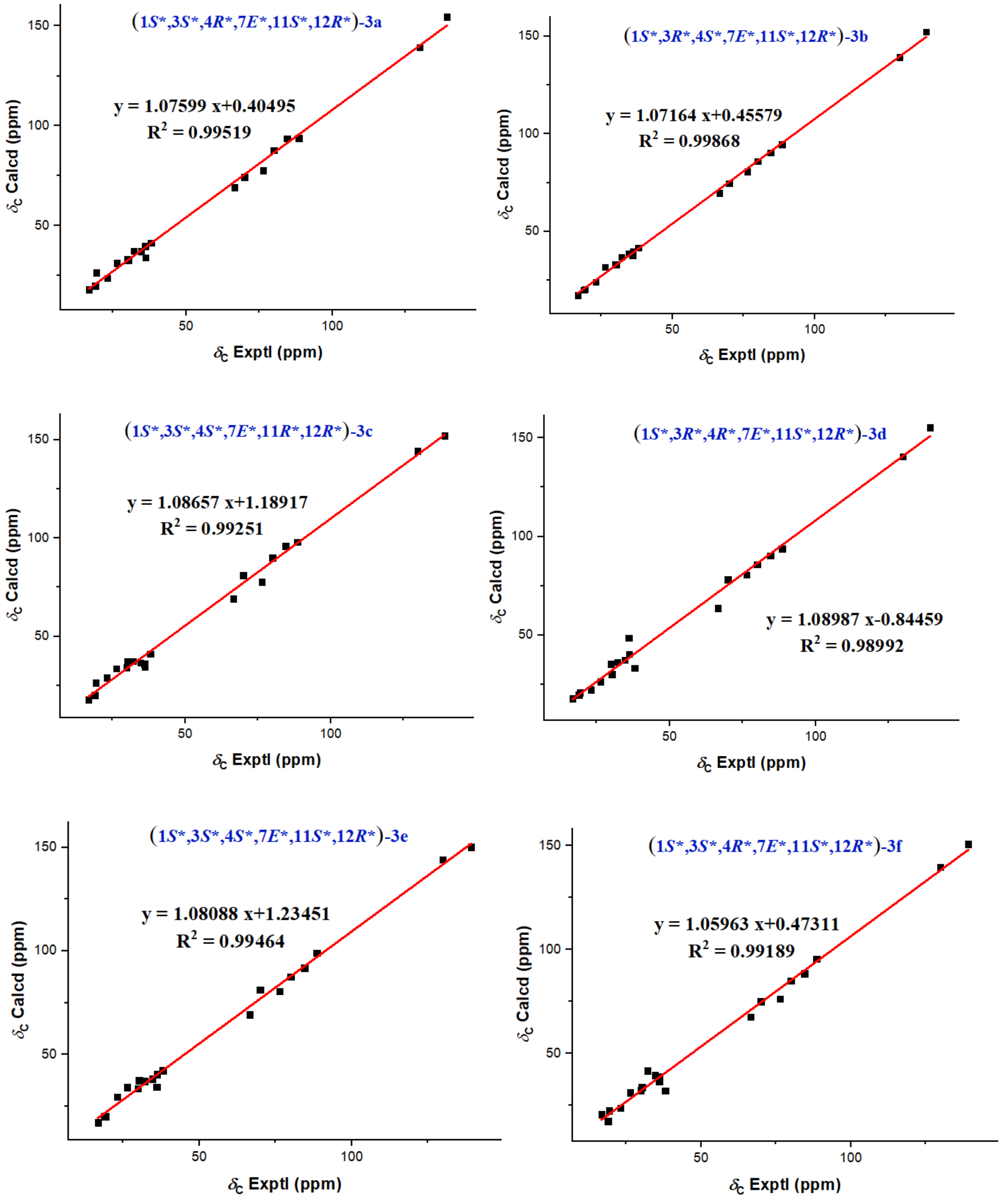

Figure S14. Regression analysis of experimental versus calculated ^{13}C NMR chemical shifts of **3** at the mPW1PW91-311g(d,p) level.

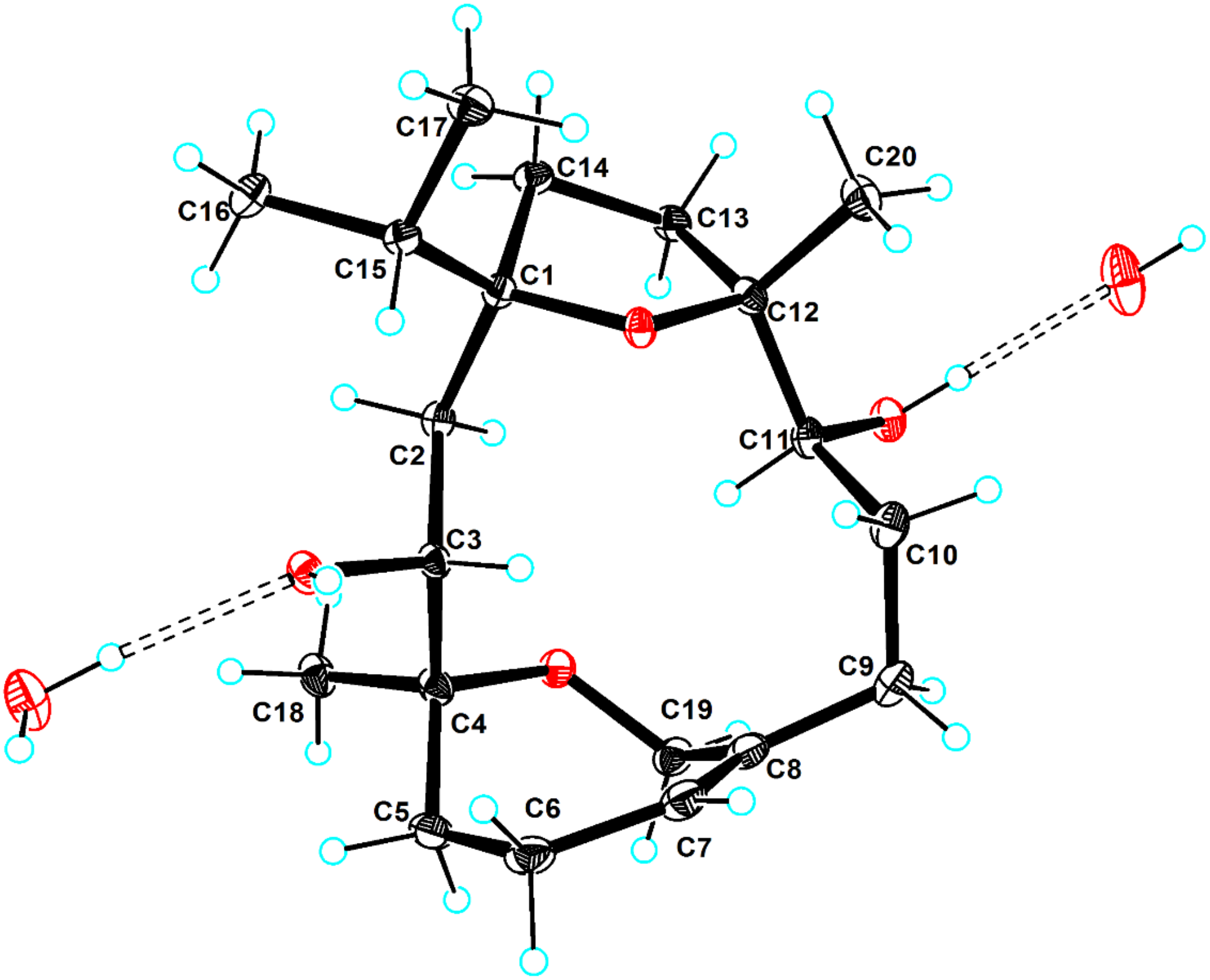

Figure S15. ORTEP drawing of X-ray structure of **3** (displacement ellipsoids are drawn at the 50% probability level).

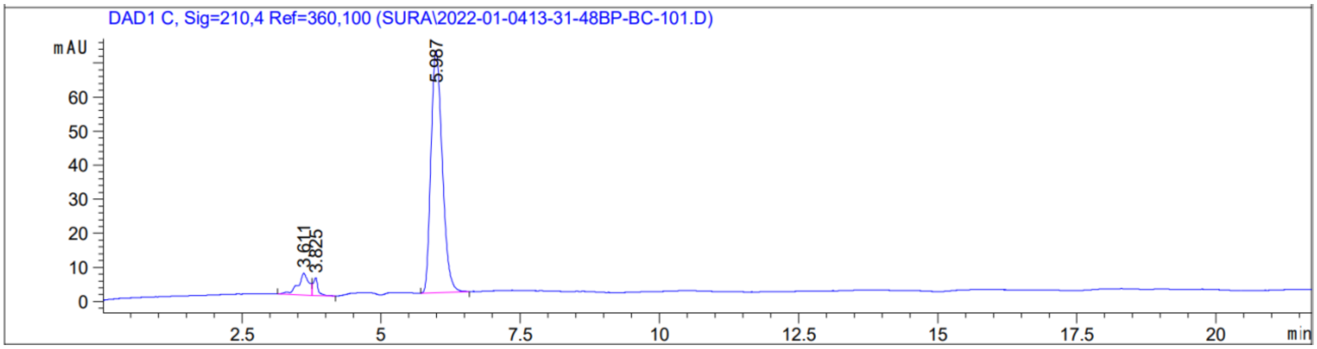

Figure S16. The chiral HPLC analysis of **3** by Daicel Chiralpack IC column (250 mm x 4.6 mm; i.d., 5 µm; Condition: *n*-hexane:ethanol 98.6:1.4; flow rate: 1 mL/min).

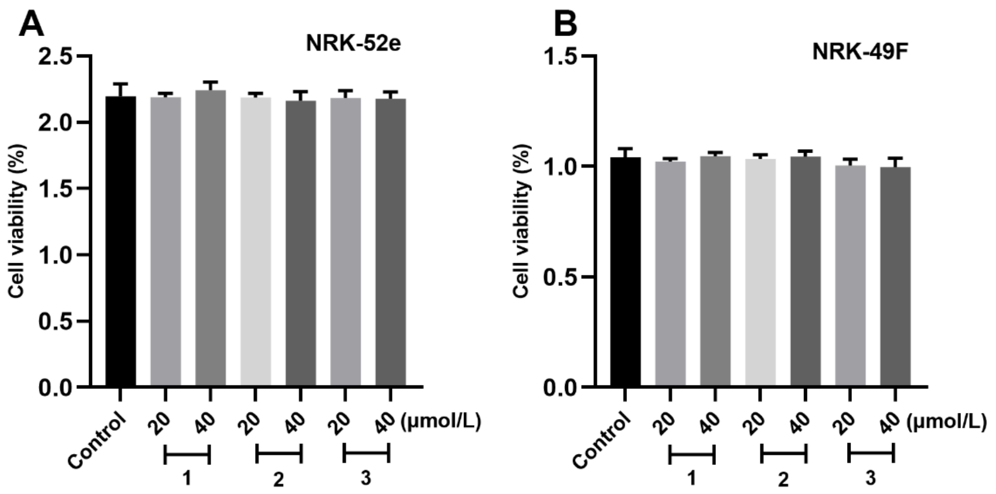

Figure S17. Cytotoxic effects of compounds in NRK-52e and NRK-49F cells. (A) NRK-52e cells were incubated with indicated amount of compounds **1**, **2** and **3** for 48 h. Cell viability was determined by CCK-8 assay. (B) NRK-49F cells were incubated with indicated amount of compounds **1**, **2** and **3** for 48 h. Cell viability was determined by CCK-8 assay. Data are presented as mean ± SEM (n=3).

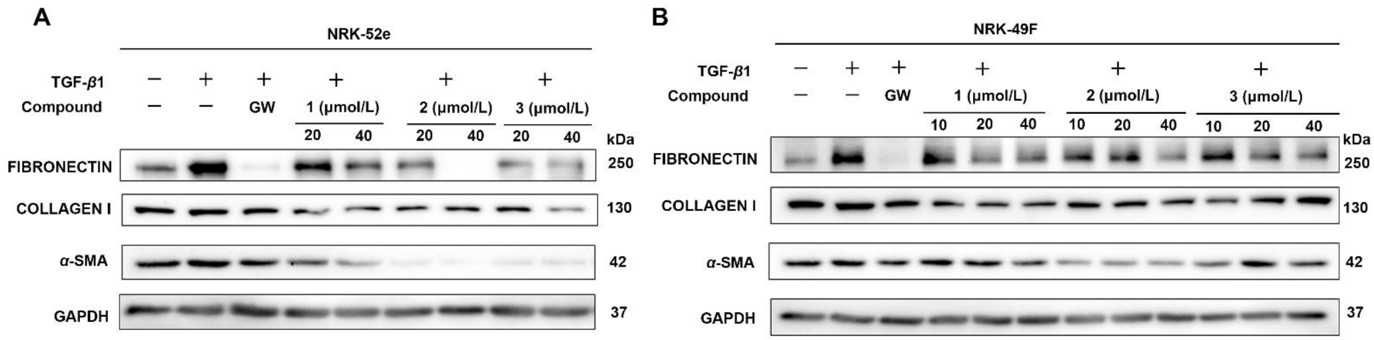

Figure S18. Compounds dose-dependently inhibit TGF-β1-induced fibrosis hallmarks in NRK-52e and NRK-49F cells. (A) NRK-52e cells were preincubated with indicated doses of compounds **1**, **2** and **3** treatments with TGF-β1 (10 ng/mL) for 48 h. Western blot was performed to examine the protein expression of α-SMA, collagen I and fibronection. GW: GW788388, GAPDH was used as an internal control. (B) NRK-49F cells were preincubated with indicated doses of compounds **1**, **2** and **3** treatments with TGF-β1 (10 ng/mL) for 48 h. Western blot was performed to examine the protein expression of α-SMA, COLLAGEN I and FIBRONECTIN. GW: GW788388, GAPDH was used as an internal control. Data are presented as mean ± SEM (n=3).

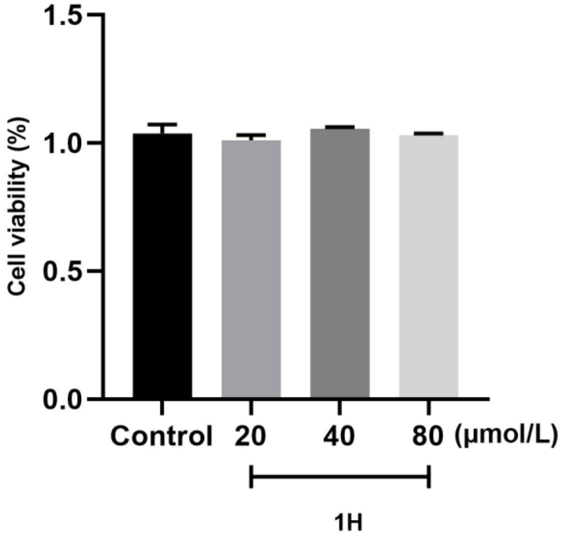

Figure S19. Cytotoxic effects of compound 1H in NRK-52e cells. NRK-52e cells were incubated with indicated amount of compound **1H** for 48 h. Cell viability was determined by CCK-8 assay. Data are presented as mean ± SEM (*n*=3).

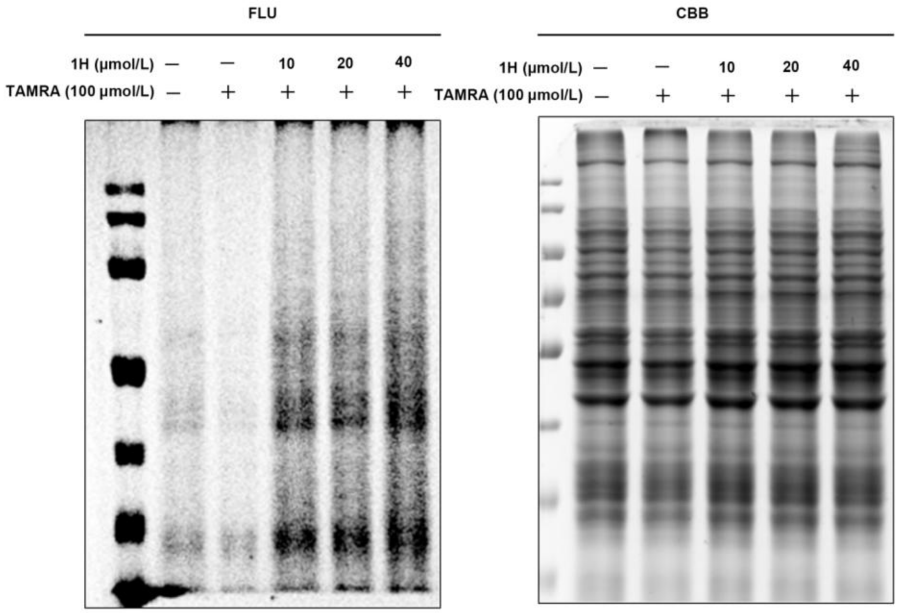

Figure S20. Strong fluorescence labeling of the probe and CBB results. Compounds fluorescently label proteins in a dose-dependent manner, and compounds label proteins in the presence or absence of different concentrations.

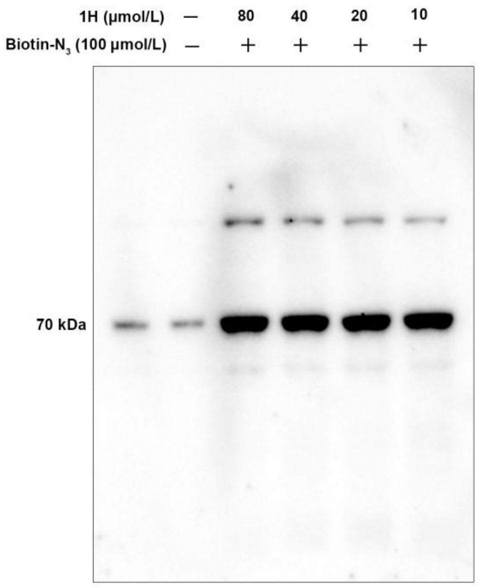

Figure S21. Strong biotin labeling of the probes. Compounds biotin label proteins in a dose-dependent manner, with labeling observed in the presence or absence of different concentrations.

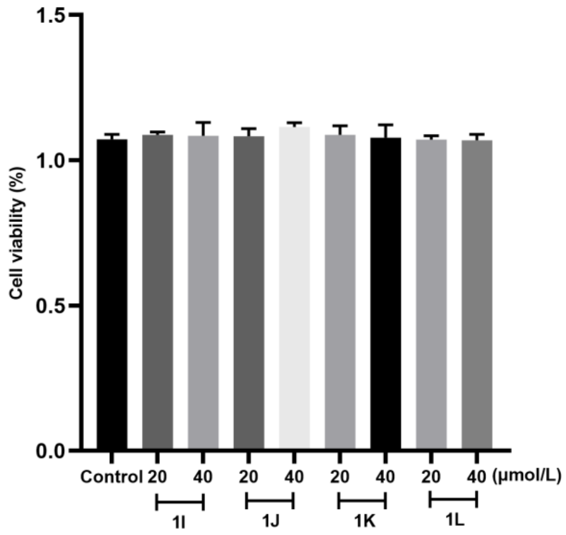

Figure S22. Cytotoxic effects of compounds 1I-1L in NRK-52e cells. Cells were incubated with indicated amount of compounds **1I-1L** for 48 h. Cell viability was determined by CCK-8 assay. Data are presented as mean ± SEM (*n*=3).

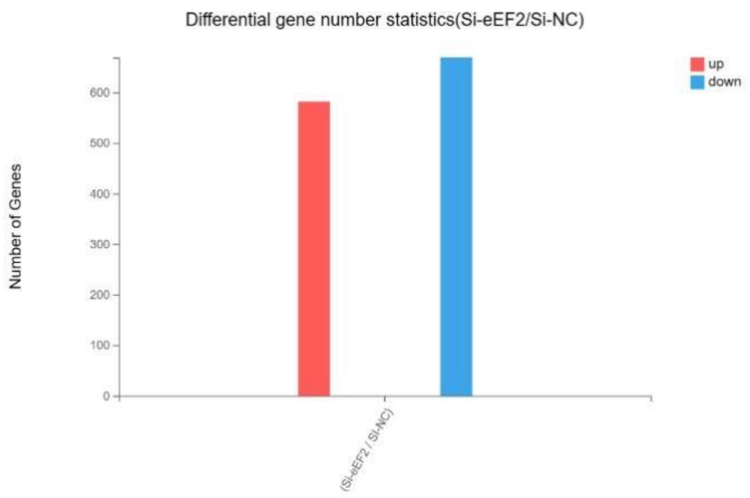

Figure S23. The gene after si-*EEF2* treatment indicates up-regulation (left side) and down-regulation (right side).

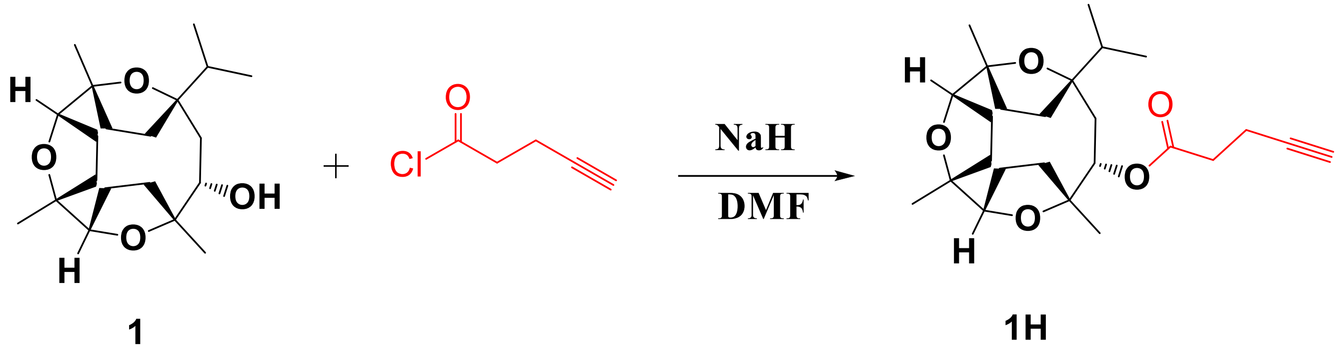

Scheme S1. Synthetic route of probe (**1H**).

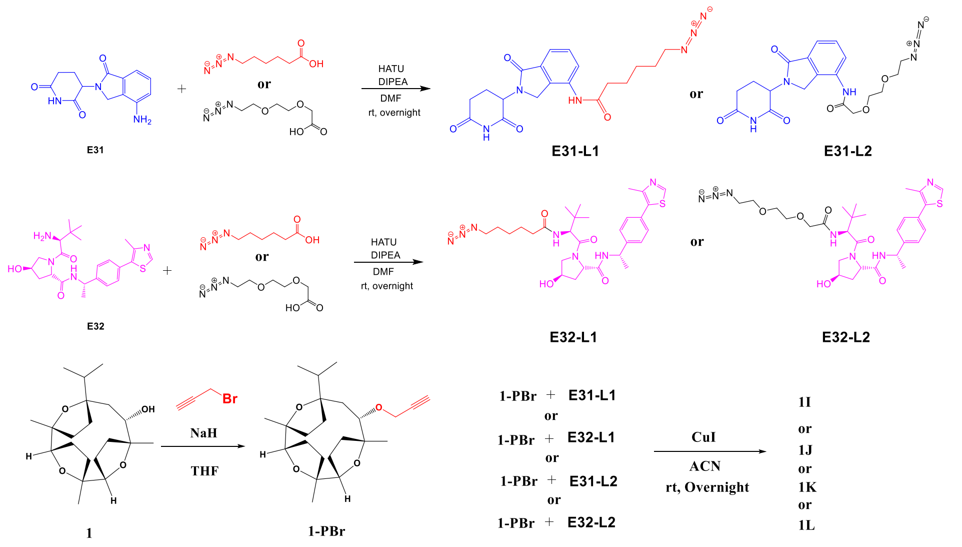

Scheme S2. Synthetic route of PROTAC molecules (**1I–1L**).

Table S1. HMBC and ROESY data of **1** in CD_3OD (δ in ppm, *J* in Hz)

**1** No.	δ_H	HMBC (H→C)	ROESY
2	Ha: 1.39, dd (15.0, 10.0)	C-1, C-3, C-15	
	Hb: 2.39, d (15.0)	C-1, C-3, C-14	
3	3.97, d (9.1)	C-1, C-2, C-4, C-18	H_b-14
5	Ha: 1.49, m	C-3, C-4, C-6, C-18	
	Hb: 2.23, m	C-4, C-7	
6	Ha: 1.54, m	C-7, C-8	
	Hb: 1.96, overlap		
7	3.80, dd (9.5, 6.5)	C-4, C-6, C-8, C-9, C-19	H_3-18, H_3-19
9	Ha: 1.62, m	C-19	
	Hb: 2.26, m	C-7, C-8, C-19	
10	1.97 overlap		
11	4.04, t (8.5)	C-8, C-12, C-20	H_3-20
13	Ha: 1.66, m	C-11, C-12	
	Hb: 1.89, ddd (15.0, 5.0)	C-11, C-12, C-20	
14	Ha: 1.75, m	C-1, C-2, C-13	
	Hb: 2.35, m	C-13	H-3
15	1.69, m	C-1, C-2, C-14, C-16, C-17	
16	0.90, d (6.8)	C-1, C-15, C-17	
17	0.93, d (6.8)	C-1, C-15, C-16	
18	1.18, s	C-3, C-4, C-5	H-7
19	1.15, s	C-7, C-8, C-9	H-7
20	1.25, s	C-11, C-12, C-13	H-11

Table S2. HMBC and ROESY data of **2** in $CDCl_3$ (δ in ppm, *J* in Hz)

	**2**		
No	δ_H	HMBC (H→C)	ROESY
2	Ha: 2.01, dd (13.0, 11.0)	C-1, C-3, C-4, C-14	
	Hb: 2.17, dd (13.5, 5.5)	C-1, C-3, C-4, C-14, C-15	
3	5.40, dd (11.0, 4.7)	C-2, C-4, C-5, C-18	H-5
5	Ha: 2.24, d (12.5)	C-3, C-4, C-6, C-7, C-18	
	Hb: 2.61, dd (12.5, 10.5)	C-3, C-4, C-6, C-7, C-18	H-3
6	3.76, m	C-7	H-7, H_3-18
7	3.16, d (8.7)	C-5, C-6, C-8, C-9, C-19	H-6, H-11
9	Ha: 1.66. m	C-10. C-11	
	Hb: 1.81, m	C-10, C-11	
10	Ha: 1.24. m	C-9. C-11	
	Hb: 1.53, m	C-9, C-11	H_3-20
11	3.30, dd (11.8, 2.2)	C-7, C-9, C-12, C-13	H-7
13	Ha: 1.51, m	C-1, C-12, C-14, C-20	
	Hb: 1.90, m	C-1, C-14, C-15, C-20	

14	Ha: 1.60, m	C-1, C-2, C-13, C-15	
	Hb: 1.93, m	C-1, C-12, C-13, C-15	
15	1.74, m	C-1, C-2, C-14, C-16, C-17	
16	0.87, d (6.8)	C-1, C-15, C-17	
17	1.00, d (6.8)	C-1, C-15, C-16	H_3-20
18	1.65, s	C-3, C-4, C-5	H-6
19	1.31, s	C-7, C-8, C-9	
20	1.11, s	C-11, C-12, C-13	H_3-17, Hb-10

Table S3. HMBC and ROESY data of **3** in $CDCl_3$ (δ in ppm, J in Hz)

	**3**		
**No.**	$\boldsymbol{\delta_H}$	**HMBC (H→C)**	**ROESY**
2	Ha: 1.44, dd (15.5, 8.2)	C-3	
	Hb: 2.15, overlap	C-1, C-3, C-4, C-14, C-15	
3	4.37, d (8.3)	C-1, C-2, C-4, C-18	
5	Ha: 1.86, m	C-6, C-7	
	Hb: 2.33, m	C-3, C-4, C-6	
6	Ha: 2.36, m	C-4, C-7, C-8	
	Hb: 2.50, m	C-4, C-5, C-7, C-8	
7	5.52, s	C-5, C-6, C-9	Ha-9
9	Ha: 1.91, overlap		H-7
	Hb: 2.21, m	C-8, C-11	
10	Ha: 1.58, m	C-8, C-9, C-11	
	Hb: 1.90, overlap		
11	3.89, d (9.5)	C-9, C-10, C-12, C-13, C-20	Ha-13
13	Ha: 1.76, m	C-1, C-12, C-14, C-20	H-11
	Hb: 2.06, m	C-1, C-11, C-12, C-14, C-20	
14	Ha: 1.40, overlap	C-12, C-13	
	Hb: 1.96, m	C-1, C-13, C-15	
15	2.18, overlap	C-1, C-14, C-16, C-17	
16	0.90, d (6.9)	C-1, C-15, C-17	H_3-20
17	0.95, d (6.7)	C-1, C-15, C-16	
18	1.06, s	C-3, C-4, C-5	
19	3.56, dd (13.3, 1.2)	C-4, C-7, C-8, C-9	
	4.94, d (13.3)	C-7, C-8, C-9	
20	1.04, s	C-11, C-12, C-13	H_3-16

Table S4. Primers for real-time PCR

**Group**	**Sense (5'—3')**	**Anti-sense (5'—3')**
GAPDH-Mus	TCCGCCCCTTCCGCTGATG	CACGGAAGGCCATGCCAGTGA
FIBRONECTIN-Mus	CCGTGGACCAGGTTGATGATACTTC	GCTCTGTGCTACTGCCTTCTACTG

COLLAGEN I-Mus	GACAGGCGAACAAGGTGACAGAG	CAGGAGAACCAGGAGAACCAGGAG
α-SMA-Mus	ACTGGGACGACATGGAAAAG	CATCTCCAGAGTCCAGCACA

Table S5. Primers for EEF2 mutation constructs

Group	Sense (5'—3')	Anti-sense (5'—3')
H108A	CATTGACTCTCCAGGTGCTGTGGACTTCTCCTC	GAGGAGAAGTCCACAGCACCTGGAGAGTCAATG
R66A	CTCGAAAGGATGAGCAGGAGGCCTGCATCACC	GGACTTGATGGTGATGCAGGCCTCCTGCTCATC
W817A	CCCAGTGTGTGTTTGACCACGCGCAGATCCTGC	GGGTCCCCAGGCAGGATCTGCGCGTGGTCAAAC
N696A	GCTCTTTGTGAGGAGGCCATGCGTGGTGTGC	GCACACCACGCATGGCCTCCTCACAAAGAG
S133A	GGTGGTGGACTGTGTGGCTGGTGTCTGTGTGC	GCACACAGACACCAGCCACACAGTCCACCACC
Q750A	GCCTATCTACCTGGTGGAGATTGCGTGTCCTGAGC	CGCCCACCACTTGCTCAGGACACGCAATCTCCACC
V402A	CTAATGATGTACATCTCCAAGATGGCGCCAACATCTGAC	CGGCCTTTGTCAGATGTTGGCGCCATCTTGGAGATGTAC
T59A	GCCGGGGAGACACGCTTCACTGACGCTCGAAAGGATGAG	GCAGCGCTCCTGCTCATCCTTTCGAGCGTCAGTGAAGCG

Table S6. siRNA sequence for *EEF2*

Group	Sense (5'—3')	Anti-sense (5'—3')
Negative control siRNA	UUCUCCGAACGUGUCACGUTT	ACGUGACACGUUCGGAGAATT
EEF2-Rat-227- siRNA	GCUUCACUGACACUCGAAATT	UUUCGAGUGUCAGUGAAGCTT
EEF2-Rat-521- siRNA	CCGUGCUGAUGAUGAACAATT	UUGUUCAUCAUCAGCACGGTT
EEF2-Rat-900- siRNA	CCCAGACGGGAAGAAACUUTT	AAGUUUCUUCCCGUCUGGGTT

Table S7. Extracted heats and weighting factors of the optimized conformers of **1** at B3LYP/6-31G(d,p) level

	Conformer	B3LYP/6-31G(d,p)	
		Extracted heats	Boltzmann-calculated contribution (%)
**1a**	1	-1083.0601	30.86 %0
	2	-1083.06009	30.24%
	3	-1083.06031	38.50%
	4	-1083.05499	0.14%
	5	-1083.05562	0.27%
**1b**	1	-1083.0601	30.98%
	2	-1083.06009	30.36%
	3	-1083.06031	38.66%
**1c**	1	-1083.04544	53.31%
	2	-1083.04532	46.70%
**1d**	1	-1083.07335	76.54%
	2	-1083.06971	01.63%
	3	-1083.07027	02.93%
	4	-1083.0715	10.85%
	5	-1083.07122	08.08%
**1e**	1	-1082.91405	0.00002%
	2	-1082.9135	0.00001%
	3	-1082.92793	45.07%
	4	-1082.92843	76.54%
	5	-1082.91331	0.000008%
	6	-1082.91361	0.00001%
	7	-1082.91388	0.00001%
	8	-1082.91322	0.000008%
	9	-1082.92717	19.96%
	10	-1082.92767	33.97%

Table S8. DP4+ results obtained for compounds **1**, **2** and **3**

Exp NMR from	Cal NMR from	sDP4+ (H data)	sDP4+ (C data)	sDP4+ (all data)	uDP4+ (H data)	uDP4+ (C data)	uDP4+ (all data)	DP4+ (H data)	DP4+ (C data)	DP4+ (all data)
1	**1a-1e**	100.00%	100.00%	100.00%	100.00%	100.00%	100.00%	100.00%	100.00%	100.00%
2	**2a-2b**	100.00%	100.00%	100.00%	100.00%	100.00%	100.00%	100.00%	100.00%	100.00%
3	**3a-3f**	99.54%	100.00%	100.00%	100.00%	100.00%	100.00%	100.00%	100.00%	100.00%

Table S9. DP4+ analysis of experimental NMR data of **1** and shielding tensors of **1a-1e**

Functional		Solvent?		Basis Set		Type of Data			
mPW1PW91		PCM		6-311+G(d,p)		Unscaled Shifts			
		DP4+	0.00%	100.00%	0.00%	0.00%	0.00%	-	-
Nuclei	sp2?	Experimental	Isomer 1	Isomer 2	Isomer 3	Isomer 4	Isomer 5	Isomer 6	Isomer 7
C		80.7	90.58231	89.24609	90.1	89.1	93.59253		
C		84.9	91.00655	91.52191	90.1	86.9	98.32943		
C		26.6	30.08999	32.00192	30.1	31.3	32.14443		
C		33.1	33.17143	36.04478	36.3	37.3	42.74808		
C		30.1	43.96899	34.66311	38.6	34.6	35.0456		
C		33.5	38.33214	38.19123	39.0	37.3	45.5592		
C		84.7	93.83268	91.88174	91.9	90.6	91.82577		
C		80.1	87.19861	89.37158	89.8	87.1	92.59961		
C		37.8	46.43805	37.96937	49.0	33.9	55.91385		
C		90.1	91.56196	92.87464	89.1	98.1	98.69308		
C		69.9	31.85194	32.24285	29.9	33.0	32.72371		
C		36.7	31.94917	41.71502	38.50	40.76	36.28331		
C		85.1	90.974	89.77391	92.39	93.94	97.17055		
C		70.2	79.29981	80.06545	80.96	78.53	86.90568		
C		40.2	42.56576	42.35127	42.00	37.79	42.82547		
C		16.6	21.58052	20.84314	19.52	17.93	19.55388		
C		16.8	20.03348	17.16308	21.40	20.64	18.79477		
C		25.1	31.92214	31.23273	25.20	30.80	31.59837		
C		17.8	26.83728	18.04828	24.41	26.84	32.38209		
C		25.5	26.77264	28.24926	29.96	21.81	35.47099		
H		4.04	3.98096	3.99656	4.03	4.05	4.26818		
H		3.8	4.24931	4.05992	4.16	4.14	4.26211		
H		1.97	2.12829	1.74129	2.07	2.16	2.0625		
H		1.97	1.93282	2.19757	2.17	1.83	2.13647		
H		2.26	2.15943	1.99686	3.11445	2.33971	2.42573		
H		1.62	1.42537	1.5802	1.42967	1.55269	1.65321		
H		2.35	1.47243	1.99686	2.06682	1.36344	1.67726		
H		1.75	2.06787	1.9725	1.77435	1.93253	1.3578		
H		1.66	1.69195	1.74129	1.42967	1.53689	2.20671		
H		1.89	1.69195	1.74129	1.57289	2.20719	4.44145		
H		1.39	2.09747	1.19241	1.61394	2.69079	1.94766		
H		2.39	1.82887	2.39087	2.44786	1.47744	2.24802		
H		1.96	2.27804	2.23908	2.06553	1.84017	2.20671		
H		1.54	1.69195	1.64166	2.43392	2.5296	1.90913		
H		1.49	1.85974	1.67983	2.52164	2.33558	2.32184		
H		2.23	1.47304	1.93859	1.50482	1.57966	1.69841		
H		3.97	4.3142	4.07937	3.94612	3.64353	4.1855		
H		1.69	1.77314	1.74129	2.21381	2.55951	1.76419		
H		0.93	1.17769	1.02259	0.72994	0.74937	0.86773		
H		0.93	1.06647	1.02259	1.42967	1.50074	1.13786		
H		0.93	0.91094	0.71486	0.61944	0.71725	0.87447		
H		0.9	0.94543	0.77124	0.81884	0.9712	0.73356		
H		0.9	1.9288	1.71147	1.64335	1.26741	1.40349		
H		0.9	0.83003	0.77124	0.68145	0.65074	0.83318		
H		1.25	1.90139	1.9725	2.12271	1.18666	1.1462		
H		1.25	1.06581	1.15719	1.2072	0.92514	2.36845		
H		1.25	0.94606	1.02259	0.72994	1.19722	0.41625		
H		1.18	1.5331	1.12298	1.09642	0.98738	2.10478		
H		1.18	1.38166	1.06428	1.65722	1.18669	1.08411		
H		1.18	0.86006	1.02259	1.06318	0.90296	1.33193		
H		1.15	1.29472	1.15719	0.84757	0.7474	1.1462		
H		1.15	0.94649	1.02259	1.17771	1.32405	1.43026		
H		1.15	0.94636	1.15719	1.2072	0.93222	1.08411		

	A–B	C	D	E	F	G	H
1	Functional	Solvent?		Basis Set		Type of Data	
2	mPW1PW91	PCM		6-311+G(d,p)		Unscaled Shifts	
3							
4		Isomer 1	Isomer 2	Isomer 3	Isomer 4	Isomer 5	Isomer 6
5	sDP4+ (H data)	0.00%	100.00%	0.00%	0.00%	0.00%	-
6	sDP4+ (C data)	0.00%	100.00%	0.00%	0.00%	0.00%	-
7	sDP4+ (all data)	0.00%	100.00%	0.00%	0.00%	0.00%	-
8	uDP4+ (H data)	0.00%	100.00%	0.00%	0.00%	0.00%	-
9	uDP4+ (C data)	0.00%	100.00%	0.00%	0.00%	0.00%	-
10	uDP4+ (all data)	0.00%	100.00%	0.00%	0.00%	0.00%	-
11	DP4+ (H data)	0.00%	100.00%	0.00%	0.00%	0.00%	-
12	DP4+ (C data)	0.00%	100.00%	0.00%	0.00%	0.00%	-
13	DP4+ (all data)	0.00%	100.00%	0.00%	0.00%	0.00%	-

Table S10. Crystal data and structure refinement of **1**

Identification code	1
Empirical formula	$C_{20}H_{34}O_4$
Formula weight	338.47
Temperature/K	100.00(10)
Crystal system	orthorhombic
Space group	$P2_12_12_1$
a/Å	10.25600(10)
b/Å	10.69330(10)
c/Å	17.1118(2)
α/°	90
β/°	90
γ/°	90
Volume/Å^3	1876.66(3)
Z	4
ρ_{calc}g/cm^3	1.198
μ/mm^{-1}	0.647
F(000)	744.0
Crystal size/mm^3	0.1 × 0.07 × 0.05
Radiation	Cu Kα (λ = 1.54184)
2Θ range for data collection/°	9.754 to 148.542
Index ranges	$-12 \leqslant h \leqslant 12$, $-9 \leqslant k \leqslant 13$, $-17 \leqslant l \leqslant 20$
Reflections collected	9959
Independent reflections	3727 [R_{int} = 0.0212, R_{sigma} = 0.0242]
Data/restraints/parameters	3727/27/253
Goodness-of-fit on F^2	1.037
Final R indexes [I>=2σ (I)]	R_1 = 0.0311, wR_2 = 0.0812
Final R indexes [all data]	R_1 = 0.0323, wR_2 = 0.0820
Largest diff. peak/hole / e Å^{-3}	0.15/-0.14
Flack parameter	-0.07(6)

Table S11. Extracted heats and weighting factors of the optimized conformers of **2** at B3LYP/6-31G(d,p) level

		B3LYP/6-31G(d,p)	
	Conformer	Extracted heats	Boltzmann-calculated contribution (%)
**2a**	1	-1083.05212	0.01%
	2	-1083.05281	0.02%
	3	-1083.05177	0.006%
	4	-1083.06006	38.50%
	5	-1083.04925	0.0004%
	6	-1083.04925	0.0004%
	7	-1083.05064	0.002%
**2b**	1	-1083.06585	23.78%
	2	-1083.0663	38.50%
	3	-1083.06541	14.92%
	4	-1083.06612	31.82%

Table S12. DP4+ analysis of experimental NMR data of **2** and shielding tensors of **2a–2b**

Functional		Solvent?		Basis Set		Type of Data	
mPW1PW91		PCM		6-311+G(d,p)		Unscaled Shifts	
		DP4+	0.00%	100.00%	-	-	-
Nuclei	sp2?	Experimental	Isomer 1	Isomer 2	Isomer 3	Isomer 4	Isomer 5
C	x	130.4	150.41202	141.07776			
C		87.3	91.25756	91.36825			
C		28.1	32.90044	31.94223			
C	x	125.8	125.91893	135.77412			
C		70.8	74.66435	75.78263			
C		39.6	43.18147	43.17045			
C		32.4	35.91531	35.89093			
C		90	95.25354	95.97546			
C		26	29.566	29.63159			
C		85.7	87.00736	89.96213			
C		85.3	89.27888	91.25383			
C		31.9	38.33398	34.98222			
C		36.3	42.95608	41.729			
C		19.1	17.27415	20.24657			
C		16.4	20.258	17.27193			
C		27.1	24.0713	28.72975			
C		17	20.58689	18.49311			
C		51.7	56.97282	54.5918			
C		21	22.42062	21.68052			
C		69.9	76.23103	74.28016			
H		3.3	2.93685	3.34579			
H		3.16	2.93731	3.05636			
H		2.17	2.56525	2.29037			
H		2.01	1.72515	2.05031			
H	x	5.4	5.85201	5.86872			
H		1.81	1.72459	1.58357			
H		1.66	1.51658	1.76076			
H		1.6	1.91738	1.71811			
H		1.93	1.91753	1.91674			
H		1.53	1.51652	1.42699			
H		1.24	1.3262	1.27768			
H		1.51	1.51663	1.49602			
H		1.9	1.51673	1.95306			
H		1.74	1.72489	1.71811			
H		0.87	0.72281	0.98158			
H		0.87	1.72486	0.98158			
H		0.87	0.91358	0.71752			
H		1	0.91371	0.71752			
H		1	0.91371	1.71811			
H		1	0.7228	0.7694			
H		1.11	1.51617	1.14673			
H		1.11	0.80826	1.03029			
H		1.11	1.06567	0.98158			
H		1.65	2.24371	2.05031			
H		1.65	1.91718	1.69434			
H		1.65	1.23083	1.47429			
H		2.24	2.4056	2.33552			
H		2.61	2.40621	2.61797			
H		1.31	0.9137	0.87212			
H		1.31	1.32602	1.32693			
H		1.31	1.725	1.65545			
H		6.76	4.09938	3.86114			

	A	B	C	D	E	F	G	H
1	Functional		Solvent?		Basis Set		Type of Data	
2	mPW1PW91		PCM		6-311+G(d,p)		Unscaled Shifts	
3								
4			Isomer 1	Isomer 2	Isomer 3	Isomer 4	Isomer 5	Isomer 6
5	sDP4+ (H data)		0.00%	100.00%	0.00%	0.00%	0.00%	0.00%
6	sDP4+ (C data)		0.00%	100.00%	0.00%	0.00%	0.00%	0.00%
7	sDP4+ (all data)		0.00%	100.00%	0.00%	0.00%	0.00%	0.00%
8	uDP4+ (H data)		0.00%	100.00%	0.00%	0.00%	0.00%	0.00%
9	uDP4+ (C data)		0.00%	100.00%	0.00%	0.00%	0.00%	0.00%
10	uDP4+ (all data)		0.00%	100.00%	0.00%	0.00%	0.00%	0.00%
11	DP4+ (H data)		0.00%	100.00%	0.00%	0.00%	0.00%	0.00%
12	DP4+ (C data)		0.00%	100.00%	0.00%	0.00%	0.00%	0.00%
13	DP4+ (all data)		0.00%	100.00%	0.00%	0.00%	0.00%	0.00%

Table S13. Crystal data and structure refinement of **2**

Identification code	2
Empirical formula	$C_{20}H_{34}O_4$
Formula weight	338.47
Temperature/K	100.00(10)
Crystal system	orthorhombic
Space group	$P2_12_12_1$
a/Å	6.48310(10)
b/Å	7.98820(10)
c/Å	36.2996(5)
α/°	90
β/°	90
γ/°	90
Volume/Å^3	1879.89(5)
Z	4
ρ_{calc}g/cm^3	1.196
μ/mm^{-1}	0.646
F(000)	744.0
Crystal size/mm^3	0.16 × 0.1 × 0.09
Radiation	Cu Kα (λ = 1.54184)
2Θ range for data collection/°	4.868 to 148.762
Index ranges	$-7 \leqslant h \leqslant 7$, $-8 \leqslant k \leqslant 9$, $-44 \leqslant l \leqslant 36$
Reflections collected	8974
Independent reflections	3696 [R_{int} = 0.0251, R_{sigma} = 0.0318]
Data/restraints/parameters	3696/0/224
Goodness-of-fit on F^2	1.068
Final R indexes [I>=2σ (I)]	R_1 = 0.0295, wR_2 = 0.0726
Final R indexes [all data]	R_1 = 0.0311, wR_2 = 0.0734
Largest diff. peak/hole / e Å^{-3}	0.17/-0.15
Flack parameter	0.01(9)

Table S14. Extracted heats and weighting factors of the optimized conformers of **3** at B3LYP/6-31G(d,p) level

		B3LYP/6-31G(d,p)	
	Conformer	Extracted heats	Boltzmann-calculated contribution (%)
	1	-1083.04425	38.50%
**3a**	2	-1083.04101	1.25%
	3	-1083.0417	2.58%
	4	-1083.03417	0.001%
	1	-1083.04322	9.55%
	2	-1083.04322	9.57%
**3b**	3	-1083.04452	38.14%
	4	-1083.04453	38.50%
	5	-1083.04341	11.70%
**3c**	1	-1083.0355	38.50%
	2	-1083.03221	1.18%
	1	-1083.03501	22.1%
	2	-1083.03543	34.56%
	3	-1083.0354	33.59%
**3d**	4	-1083.03553	38.50%
	5	-1083.03537	32.53%
	6	-1083.02786	0.011%
	1	-1083.03843	29.86%
**3e**	2	-1083.03867	8.50%
	3	-1083.03866	38.14%
	1	-1083.04182	3.77%
	2	-1083.0428	10.59%
	3	-1083.03499	0.003%
	4	-1083.03641	0.012%
**3f**	5	-1083.04258	8.43%
	6	-1083.04402	38.50%
	7	-1083.04294	12.37%

8	-1083.04207	4.90%
9	-1083.04278	10.40%

Table S15. DP4+ analysis of experimental NMR data of **2** and shielding tensors of **3a-3f**

Functional		Solvent?	Basis Set		Type of Data			
mPW1PW91		PCM	6-311+G(d,p)		Unscaled Shifts			
		DP4+	0.00%	100.00%	0.00%	0.00%	0.00%	0.00%	-
Nuclei	sp2?	Experimental	Isomer 1	Isomer 2	Isomer 3	Isomer 4	Isomer 5	Isomer 6	Isomer 7
C		80	87.82345	86.04267	90.1	85.7	87.59523	85.0404	
C	x	130	139.35464	139.29659	144.2	140.5	144.09588	139.75889	
C		36	39.72826	37.90006	36.1	48.6	34.34566	36.60781	
C		69.9	74.44694	74.5855	81.1	78.2	81.31469	75.04586	
C	x	139.3	154.51305	152.2886	152.1	155.3	150.16975	150.75667	
C		34.6	37.23734	38.81087	36.7	37.4	38.10533	39.52318	
C		29.8	33.19519	33.29277	34.3	35.2	33.58237	32.33919	
C		88.6	93.87398	94.73941	98.0	93.9	98.99889	95.54197	
C		38	41.3561	41.65955	41.1	33.2	42.24593	32.19388	
C		76.4	77.76221	80.64615	77.7	80.6	80.42366	76.32383	
C		84.6	93.62451	90.40263	96.0	90.2	91.81319	88.42148	
C		36.1	34.10251	39.60338	34.53	40.38	40.50453	38.9542	
C		32.1	37.39421	36.94822	37.43	36.24	37.06807	41.86841	
C		16.7	18.091	17.35683	17.81	17.84	17.0531	20.81345	
C		18.9	19.99186	20.0053	20.15	19.63	20.16352	17.44854	
C		19.2	26.44018	20.39345	26.35	20.79	20.00149	22.65249	
C		23	23.78117	24.06726	29.13	22.32	29.44138	23.76989	
C		30.2	32.86046	32.83985	37.46	30.14	37.53536	33.83872	
C		66.5	69.11725	69.76438	69.11	63.65	69.31559	67.6713	
C		26.3	31.38878	31.76763	33.69	26.32	34.16289	31.26493	
H	x	5.52	5.88512	5.92362	6.01	6.25	5.99854	5.88681	
H		1.44	1.59352	1.48766	2.15	1.93	1.69176	2.34932	
H		2.15	1.92012	2.13996	1.90	2.87	2.52544	0.94475	
H		4.37	4.5402	4.54474	3.94	3.75	3.95441	4.0552	
H		2.21	2.30963	2.25674	2.32416	2.49817	2.25147	2.17815	
H		1.91	2.11254	2.01133	2.14627	2.34933	2.13311	2.14728	
H		1.96	2.11254	2.07556	2.15142	2.17746	2.08504	1.87525	
H		1.4	1.42252	1.41096	1.43643	1.56013	1.39375	2.12779	
H		1.9	2.11911	1.92471	1.89097	2.31744	1.79505	2.36263	
H		1.58	2.11254	1.63827	2.14627	1.50611	1.58047	1.12277	
H		3.89	3.36043	4.10851	3.41892	3.46564	4.56564	3.4793	
H		1.76	1.30369	1.75516	1.4279	1.82059	1.81591	1.76571	
H		2.06	2.90309	1.95173	3.06612	2.11064	2.21045	2.32629	
H		2.18	2.42169	2.46508	2.98141	2.19042	2.85265	1.74545	
H		0.9	0.78074	0.72884	0.73364	1.0381	0.69736	1.01465	
H		0.9	1.43151	1.52155	1.40731	1.54045	1.49713	1.00662	
H		0.9	0.78318	0.75145	0.7265	0.92069	0.69736	0.66114	
H		0.95	0.79727	0.8954	0.98295	1.05387	0.99038	0.73217	
H		0.95	1.28493	1.31654	1.1019	1.047	1.11887	1.68155	
H		0.95	0.56646	0.53734	0.6547	0.76655	0.59608	0.66114	
H		1.04	0.81655	1.26794	0.73364	1.0741	1.01688	1.26591	
H		1.04	1.29052	0.91736	1.33512	1.19821	1.03977	1.31116	
H		1.04	1.03981	0.98939	1.09921	1.0034	1.11342	0.81677	
H		1.06	1.39271	1.29975	0.72438	0.76375	0.69736	1.01465	
H		1.06	1.0539	0.98939	1.08818	1.38502	1.01229	0.96432	
H		1.06	0.79729	0.77087	0.99009	1.92661	0.99038	0.92309	
H		1.86	1.77478	1.81988	2.38605	1.52682	1.92002	1.80356	
H		2.33	2.41591	2.3705	1.7586	1.24344	2.27141	2.40515	
H		4.94	4.95947	5.08368	4.92821	3.81051	4.63996	5.03617	
H		3.56	3.89693	3.5491	3.94525	2.99194	4.04968	3.57482	
H		2.36	2.33701	2.296	3.10155	1.81047	3.06336	2.70686	
H		2.5	2.73594	2.71212	2.55648	2.31838	2.49967	2.57178	

	A–B	C	D	E	F	G	H
1	Functional	Solvent?		Basis Set		Type of Data	
2	mPW1PW91	PCM		6-311+G(d,p)		Unscaled Shifts	
3							
4		Isomer 1	Isomer 2	Isomer 3	Isomer 4	Isomer 5	Isomer 6
5	sDP4+ (H data)	0.46%	99.54%	-	-	-	-
6	sDP4+ (C data)	0.00%	100.00%	-	-	-	-
7	sDP4+ (all data)	0.00%	100.00%	-	-	-	-
8	uDP4+ (H data)	0.00%	100.00%	-	-	-	-
9	uDP4+ (C data)	0.00%	100.00%	-	-	-	-
10	uDP4+ (all data)	0.00%	100.00%	-	-	-	-
11	DP4+ (H data)	0.00%	100.00%	-	-	-	-
12	DP4+ (C data)	0.00%	100.00%	-	-	-	-
13	DP4+ (all data)	0.00%	100.00%	-	-	-	-

Table S16. Crystal data and structure refinement of **3**

Identification code	**3**
Empirical formula	$C_{20}H_{38}O_6$
Formula weight	374.50
Temperature/K	100.00(10)
Crystal system	orthorhombic
Space group	$P2_12_12_1$
a/Å	7.2811(2)
b/Å	16.2632(4)
c/Å	17.5475(4)
α/°	90
β/°	90
γ/°	90
Volume/Å^3	2077.87(9)
Z	4
ρ_{calc}g/cm^3	1.197
μ/mm^{-1}	0.702
F(000)	824.0
Crystal size/mm^3	0.2 × 0.06 × 0.05
Radiation	Cu Kα (λ = 1.54184)
2Θ range for data collection/°	7.412 to 148.65
Index ranges	$-9 \leqslant h \leqslant 8, -20 \leqslant k \leqslant 19, -15 \leqslant l \leqslant 21$

Reflections collected	10073
Independent reflections	4098 [R_{int} = 0.0262, R_{sigma} = 0.0304]
Data/restraints/parameters	4098/0/247
Goodness-of-fit on F^2	1.046
Final R indexes [I>=2σ (I)]	R_1 = 0.0333, wR_2 = 0.0867
Final R indexes [all data]	R_1 = 0.0347, wR_2 = 0.0876
Largest diff. peak/hole / e Å^{-3}	0.39/-0.17
Flack parameter	0.00(7)

Table S17. Predicted binding sites and mutated amino acids of compounds to EEF2 protein

Binding site	Amino acids site	Distance (Å)
108	HIS (H)	2.75
66	ARG (R)	3.11
817	TRP (W)	2.94
696	ASN (N)	2.96
133	SER (S)	2.91
750	GLN (Q)	3.1
402	VAL (V)	3.09
59	THR (T)	3.12

ESIHRMS, NMR, and chiral HPLC spectra of 1–3:

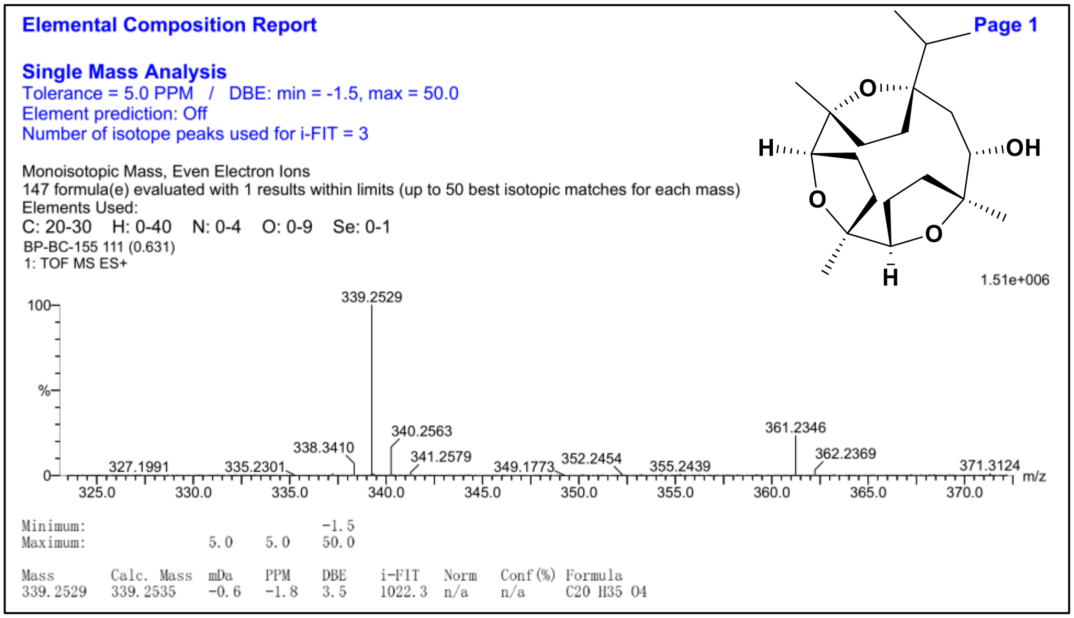

Figure S24. ESIHRMS of **1**.

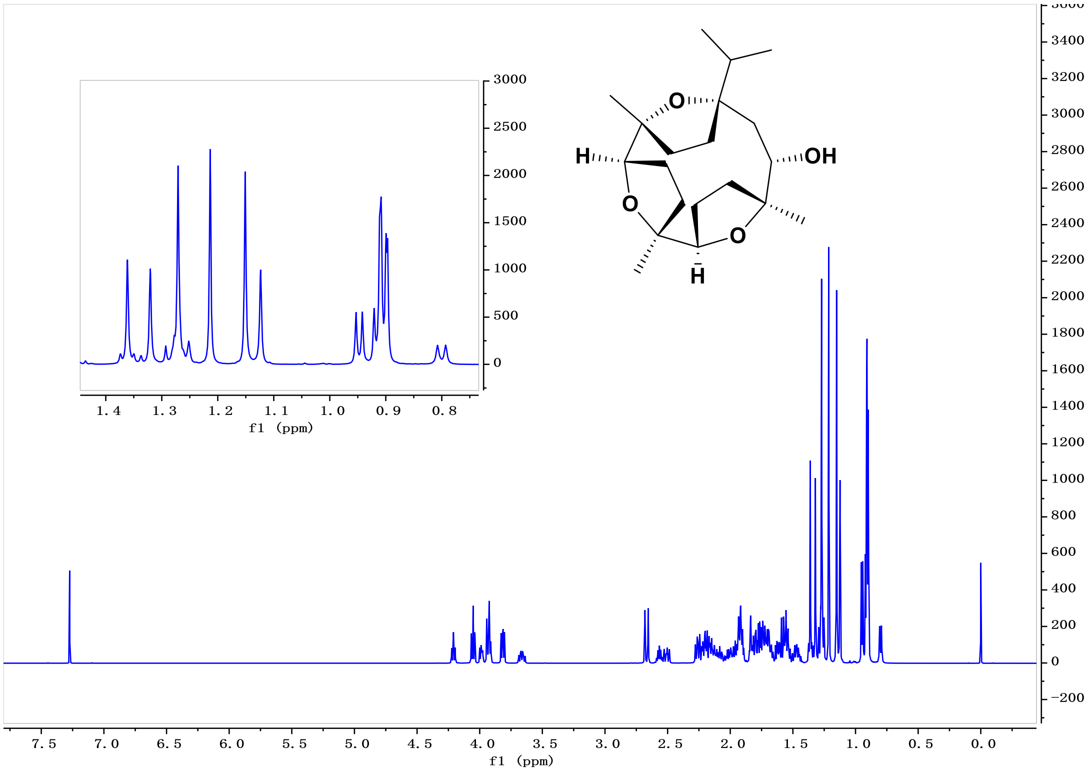

Figure S25. ^{1}H NMR spectrum of **1** ($CDCl_3$, 500 MHz).

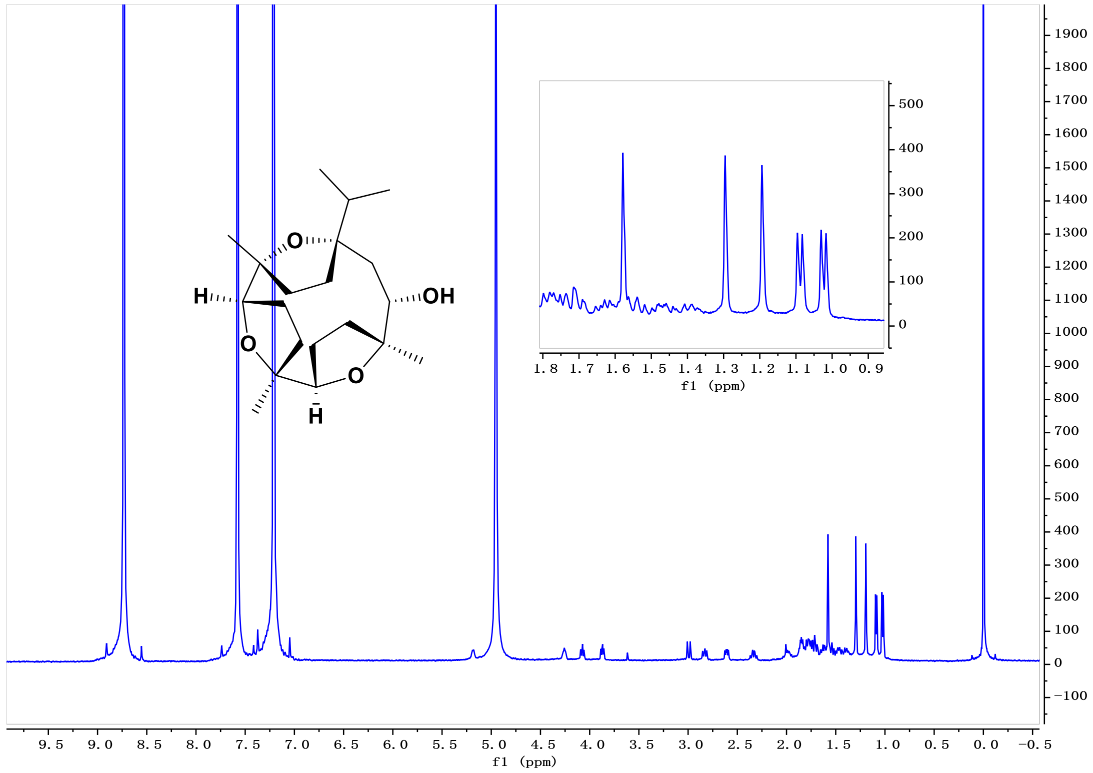

Figure S26. ^{1}H NMR spectrum of **1** (pyridine-d_5, 500 MHz).

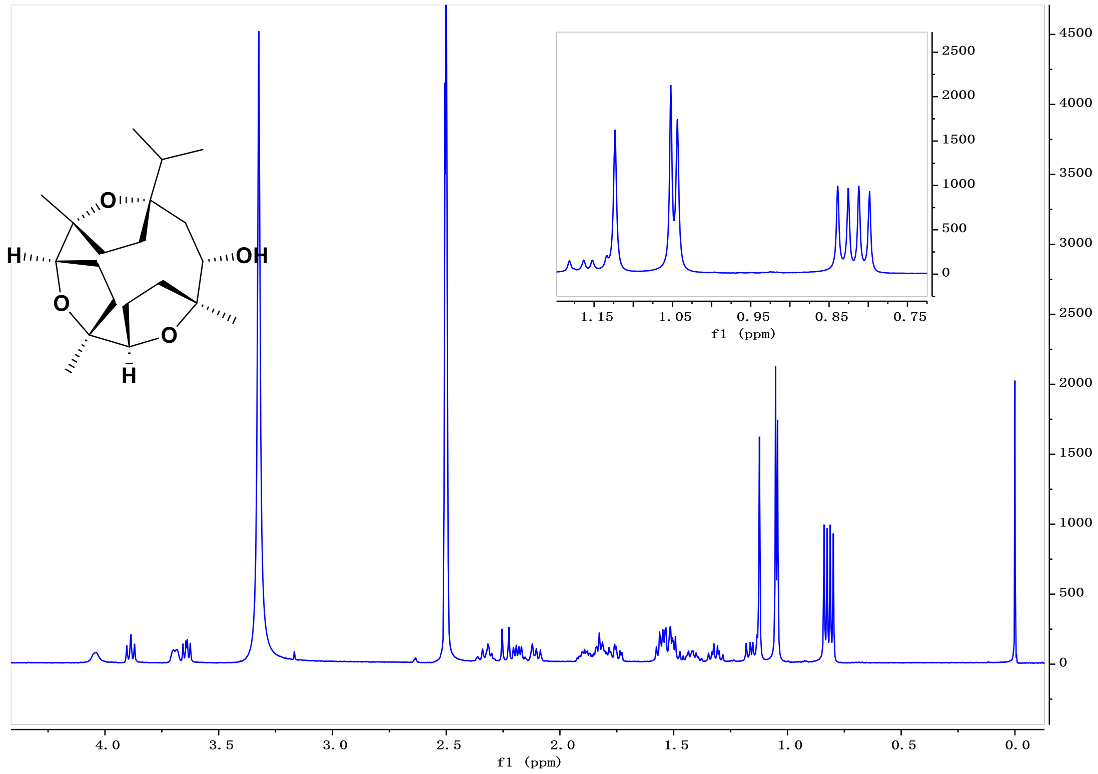

Figure S27. ^{1}H NMR spectrum of **1** (DMSO-d_6, 500 MHz).

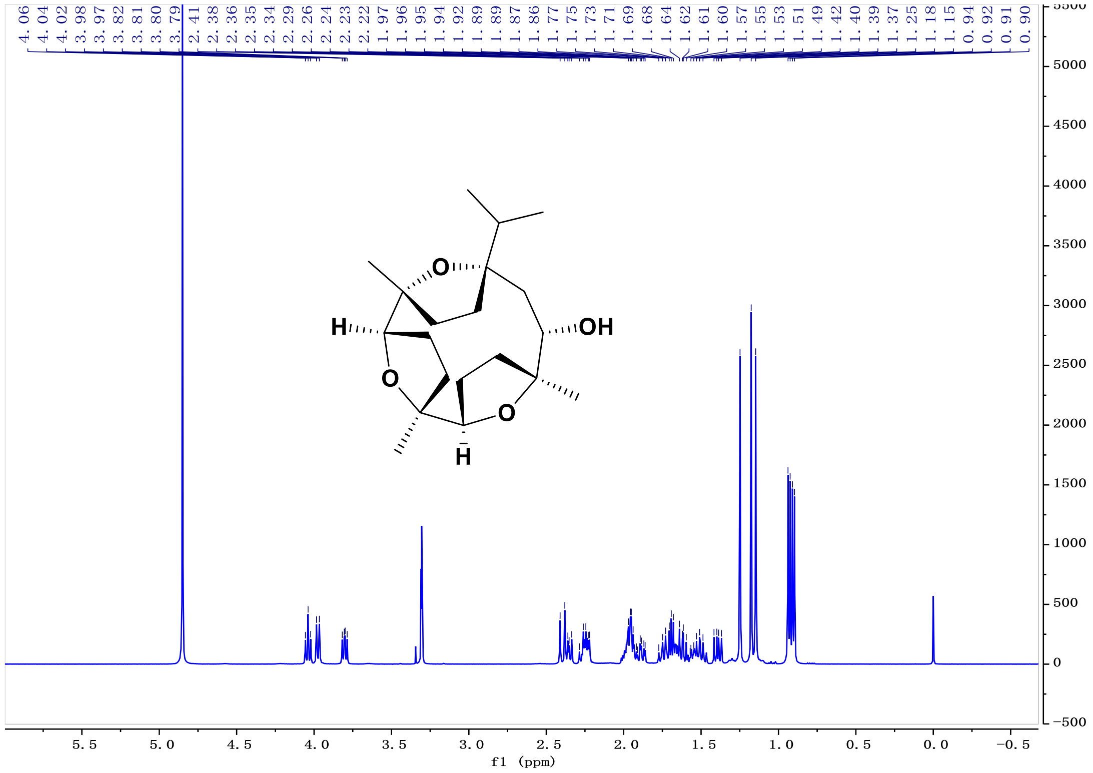

Figure S28. ^{1}H NMR spectrum of **1** (CD_3OD, 500 MHz).

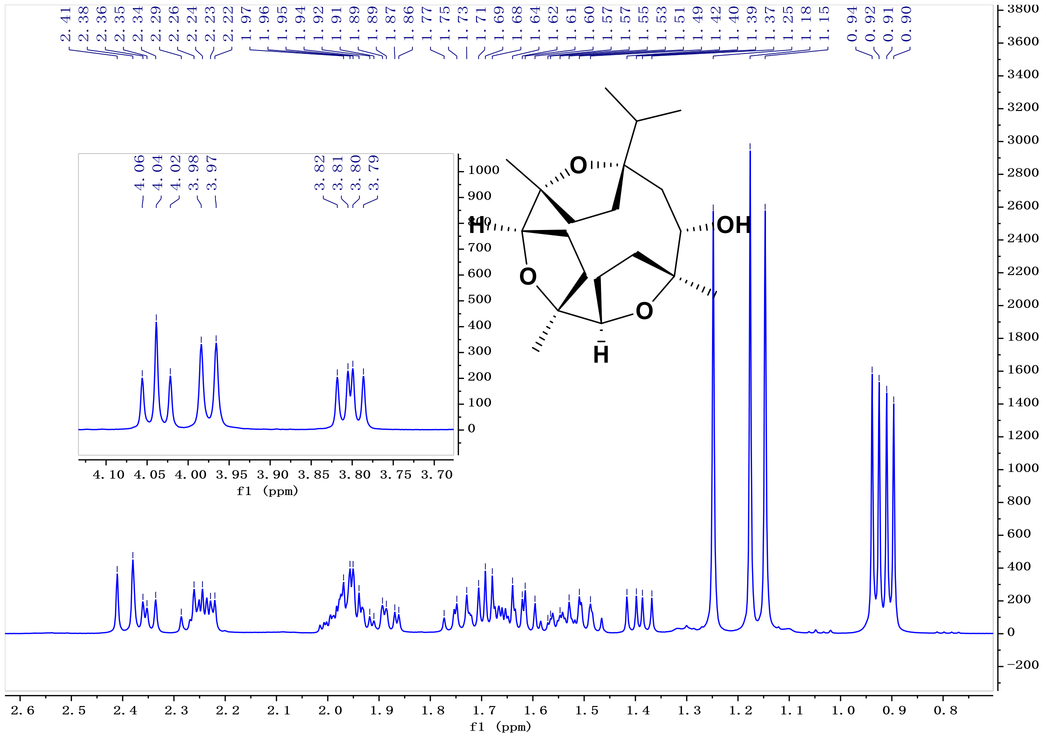

Figure S29. ^{1}H NMR expansion spectrum of **1** (CD_3OD, 500 MHz).

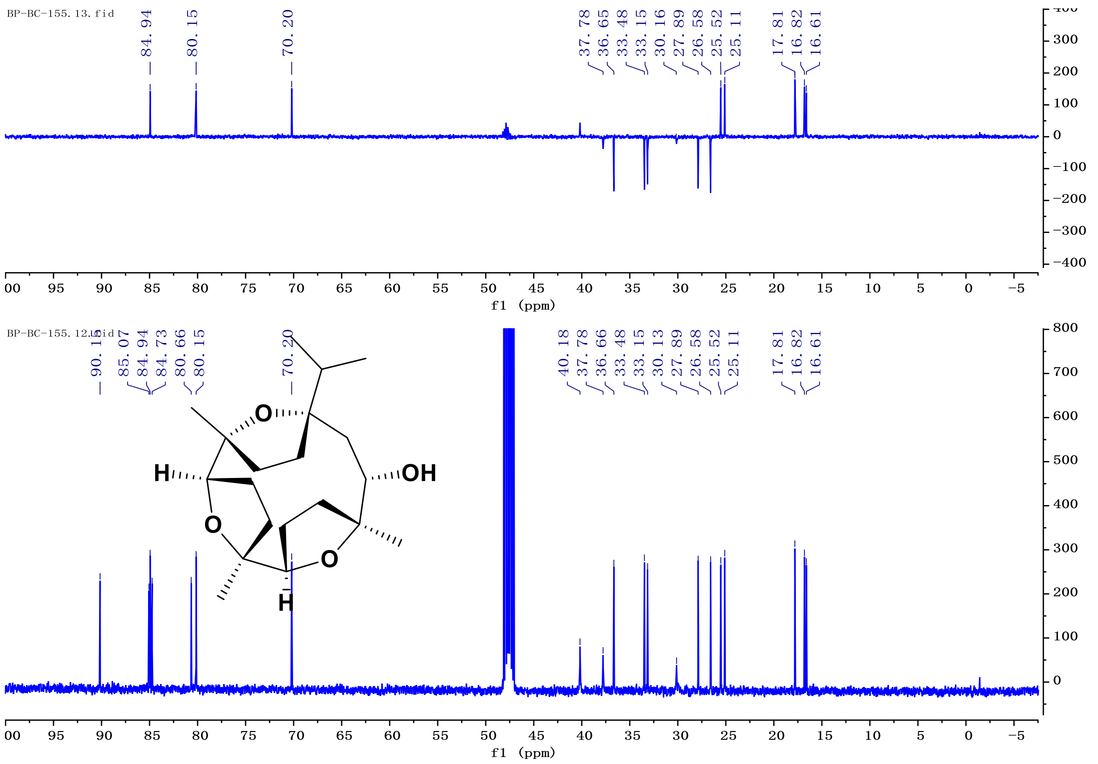

Figure S30. ^{13}C and DEPT-135 NMR spectra of **1** (CD_3OD, 125 MHz).

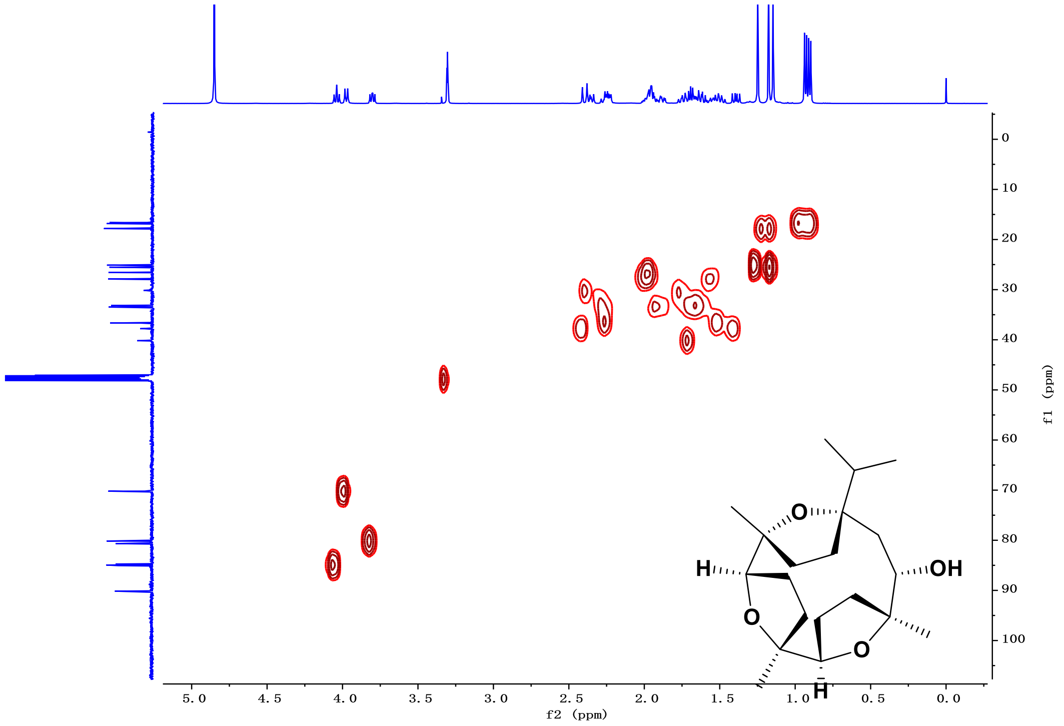

Figure S31. HSQC spectrum of **1** (CD_3OD, 500 MHz).

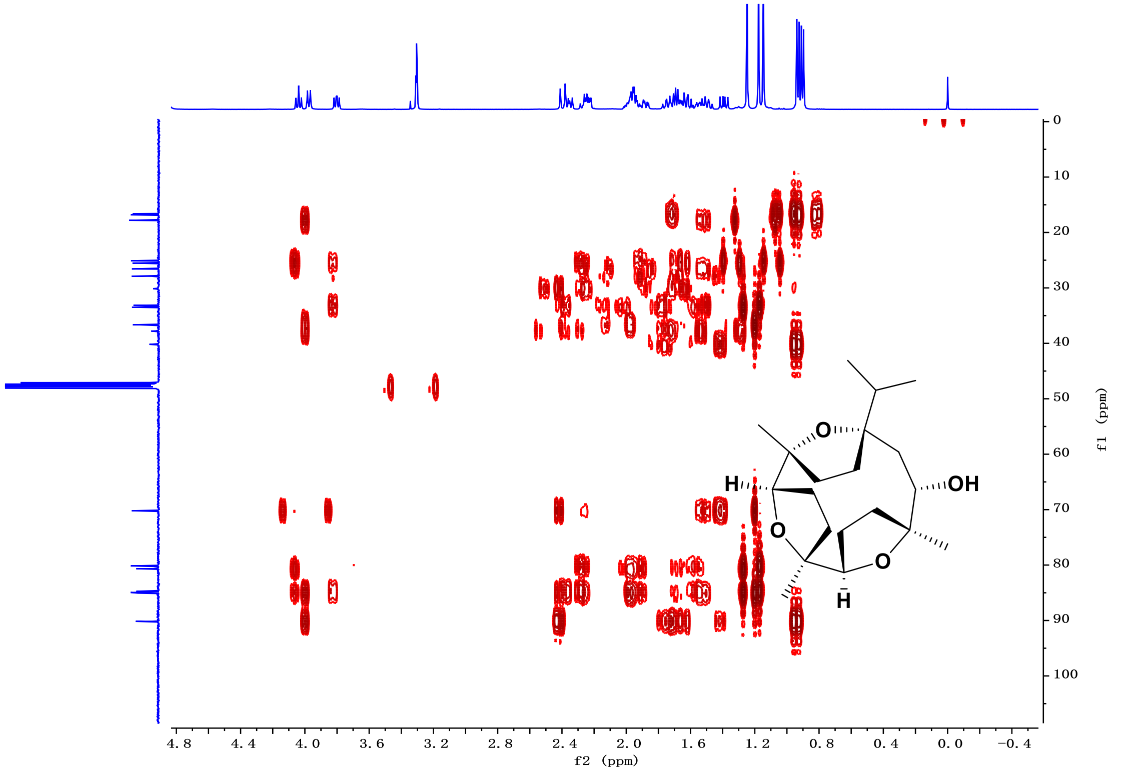

Figure S32. HMBC spectrum of **1** (CD_3OD, 500 MHz).

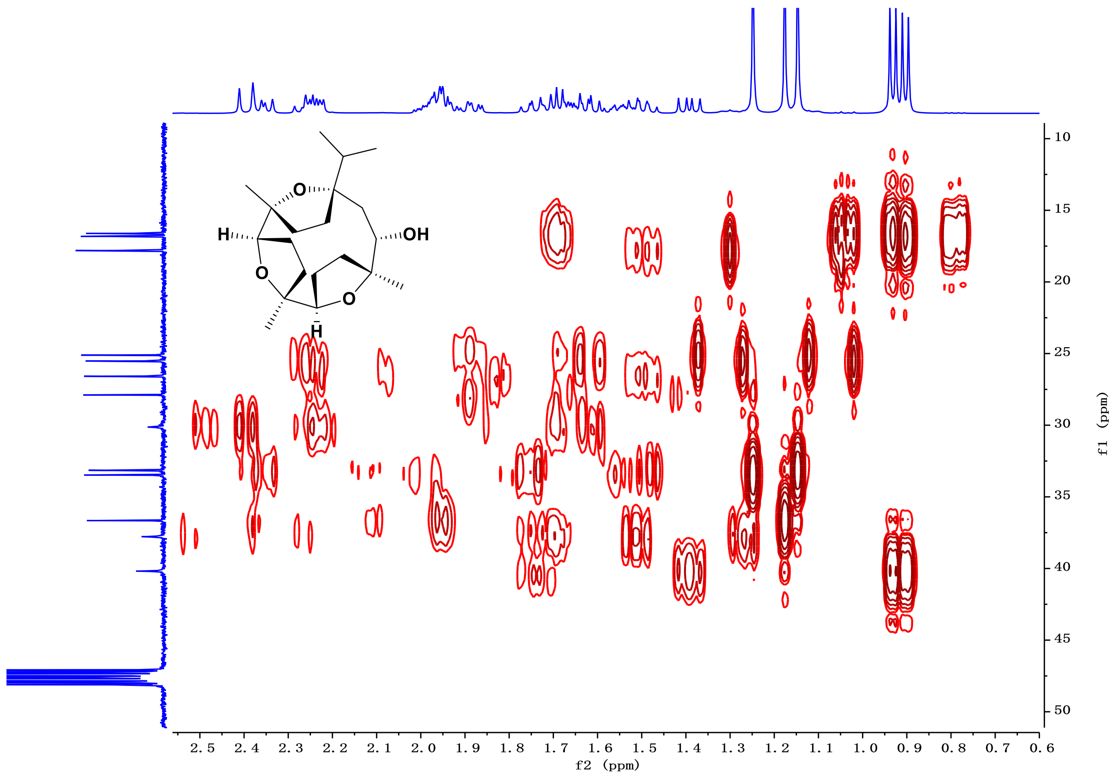

Figure S33. HMBC expansion spectrum of **1** (CD_3OD, 500 MHz).

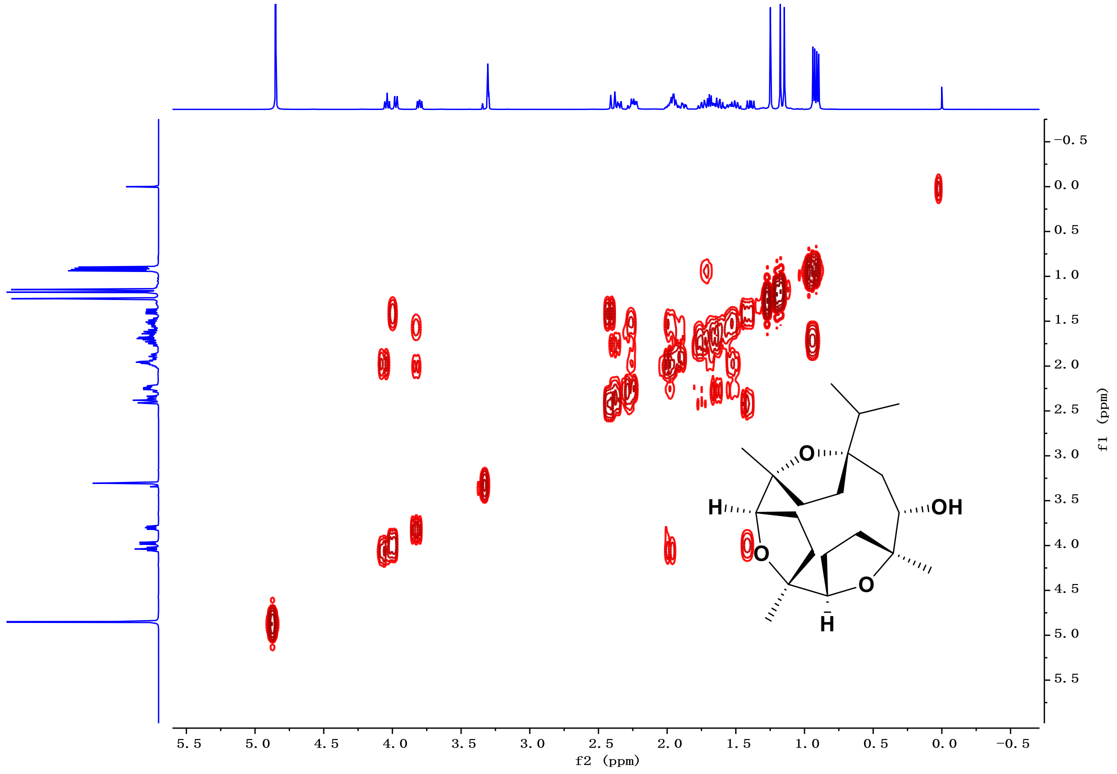

Figure S34. COSY spectrum of **1** (CD_3OD, 500 MHz).

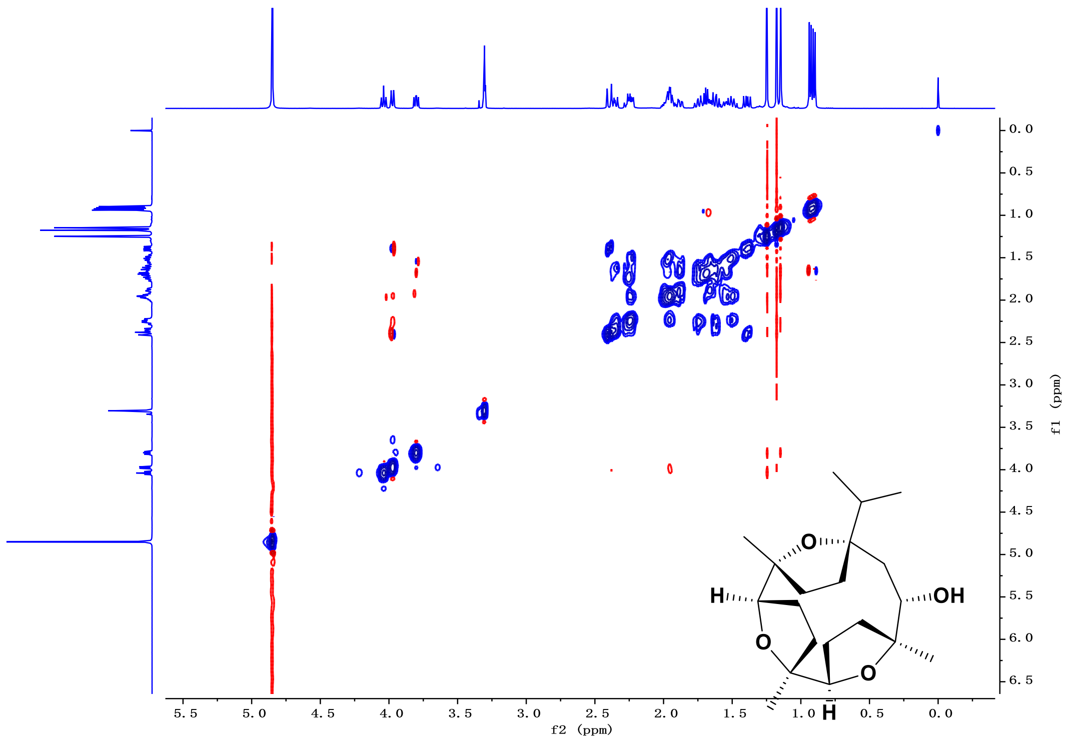

Figure S35. ROESY spectrum of **1** (CD_3OD, 500 MHz).

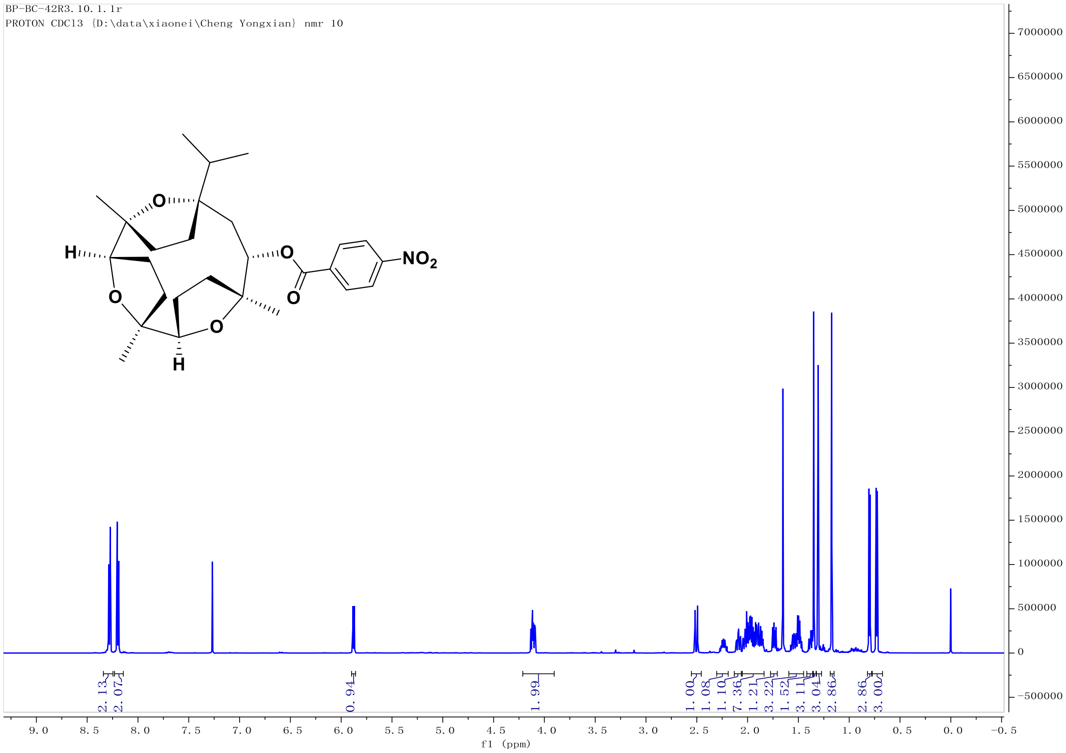

Figure S36. ^{1}H NMR spectrum of *p*-nitrobenzoic ester derivative of **1** ($CDCl_3$, 500 MHz).

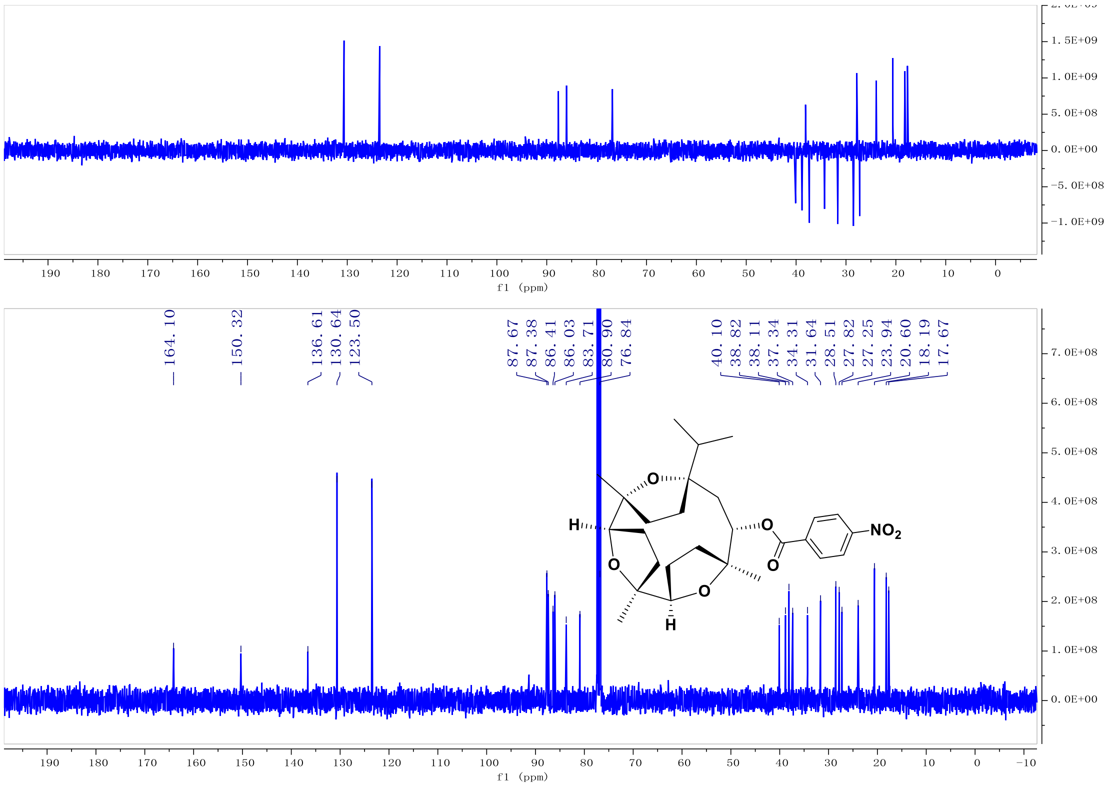

Figure S37. ^{13}C and DEPT-135 NMR spectra of *p*-nitrobenzoic ester derivative of **1** ($CDCl_3$, 125 MHz).

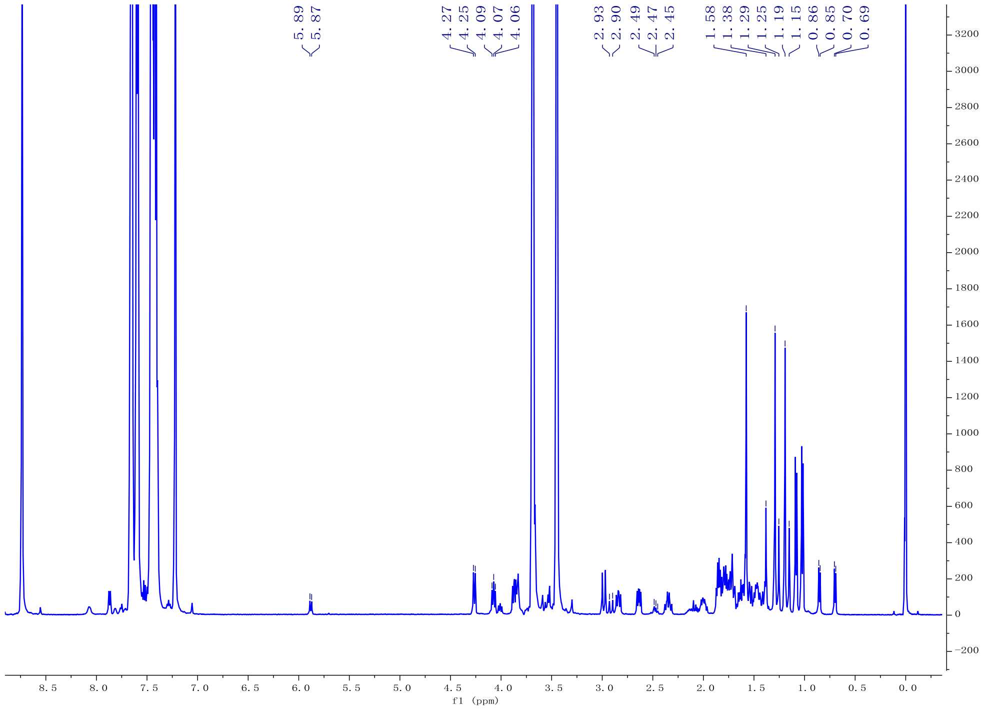

Figure S38. ^{1}H NMR spectrum of *S*-MTPA ester derivative of **1** (pyridine-d_5, 600 MHz).

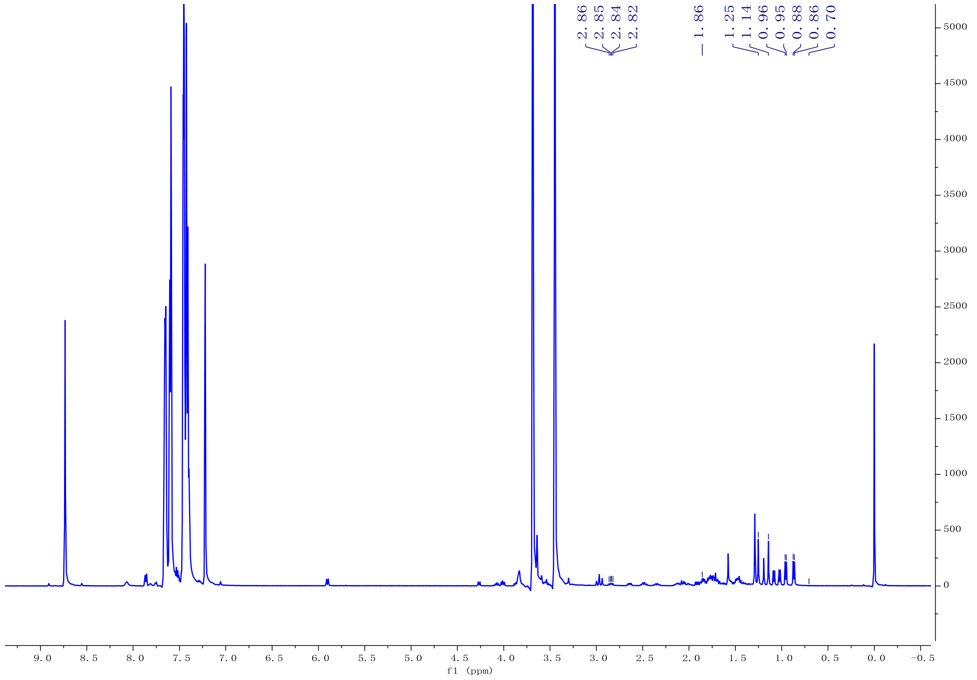

Figure S39. ^{1}H NMR spectrum of *R*-MTPA ester derivative of **1** (pyridine-d_5, 600 MHz).

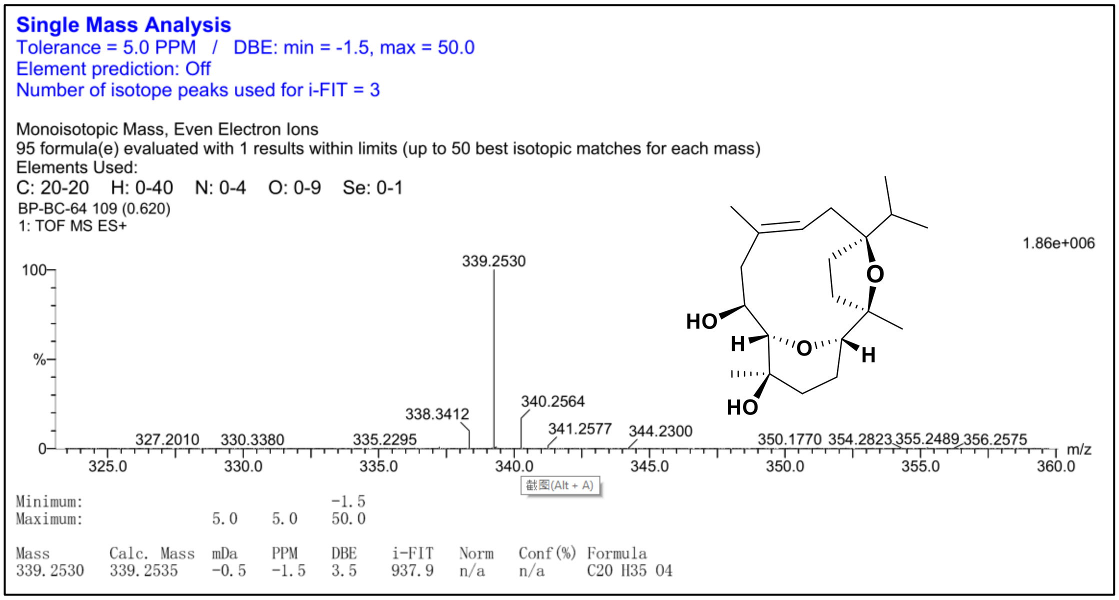

Figure S40. ESIHRMS of **2**.

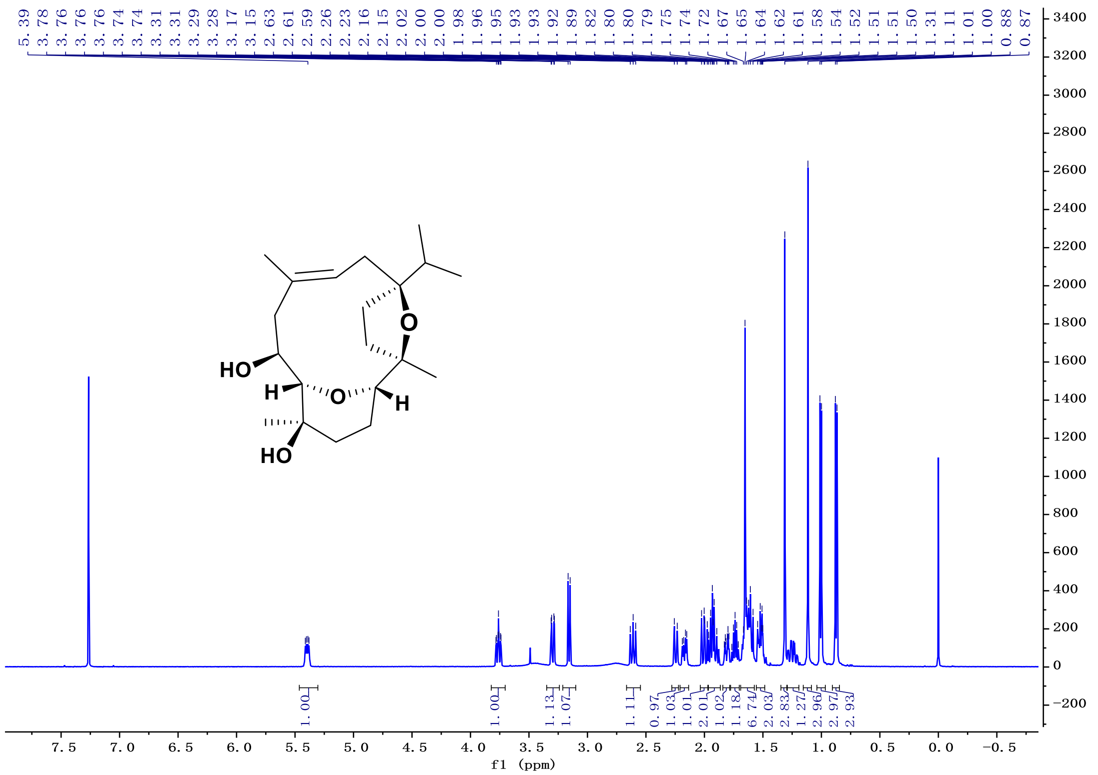

Figure S41. ^{1}H NMR spectrum of **2** ($CDCl_3$, 500 MHz).

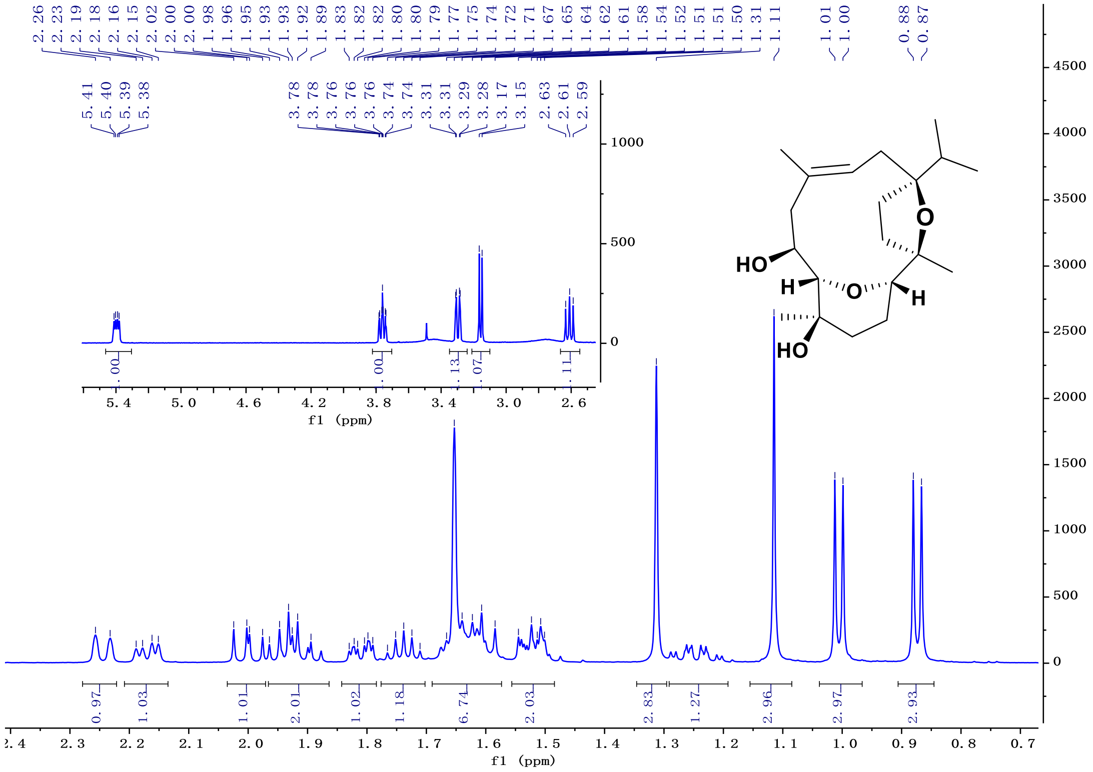

Figure S42. ^{1}H NMR expansion spectrum of **2** ($CDCl_3$, 500 MHz).

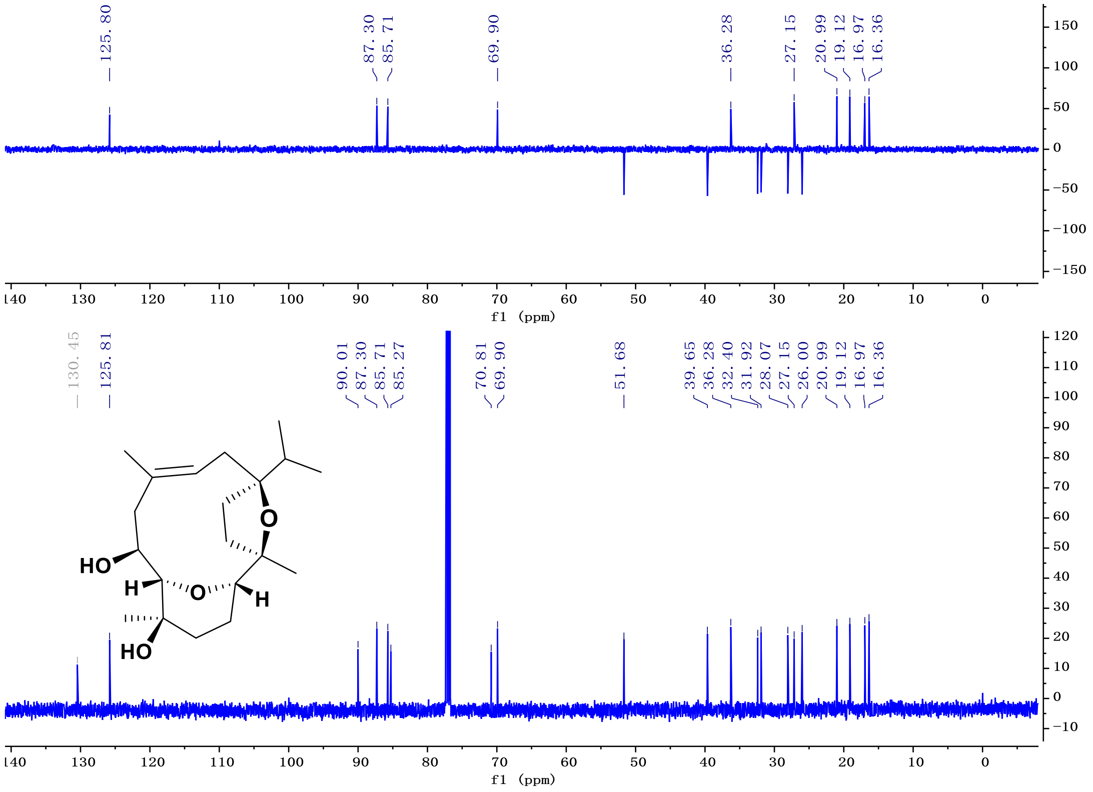

Figure S43. ^{13}C and DEPT-135 NMR spectra of **2** ($CDCl_3$, 125 MHz).

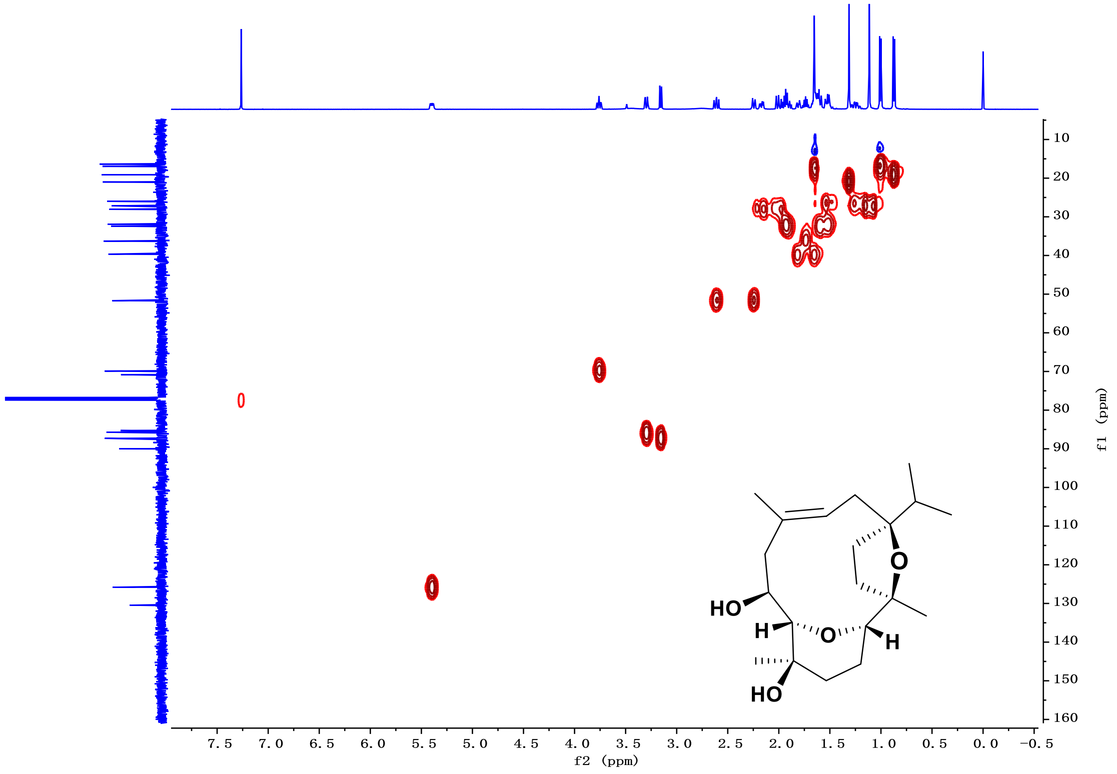

Figure S44. HSQC spectrum of **2** ($CDCl_3$, 500 MHz).

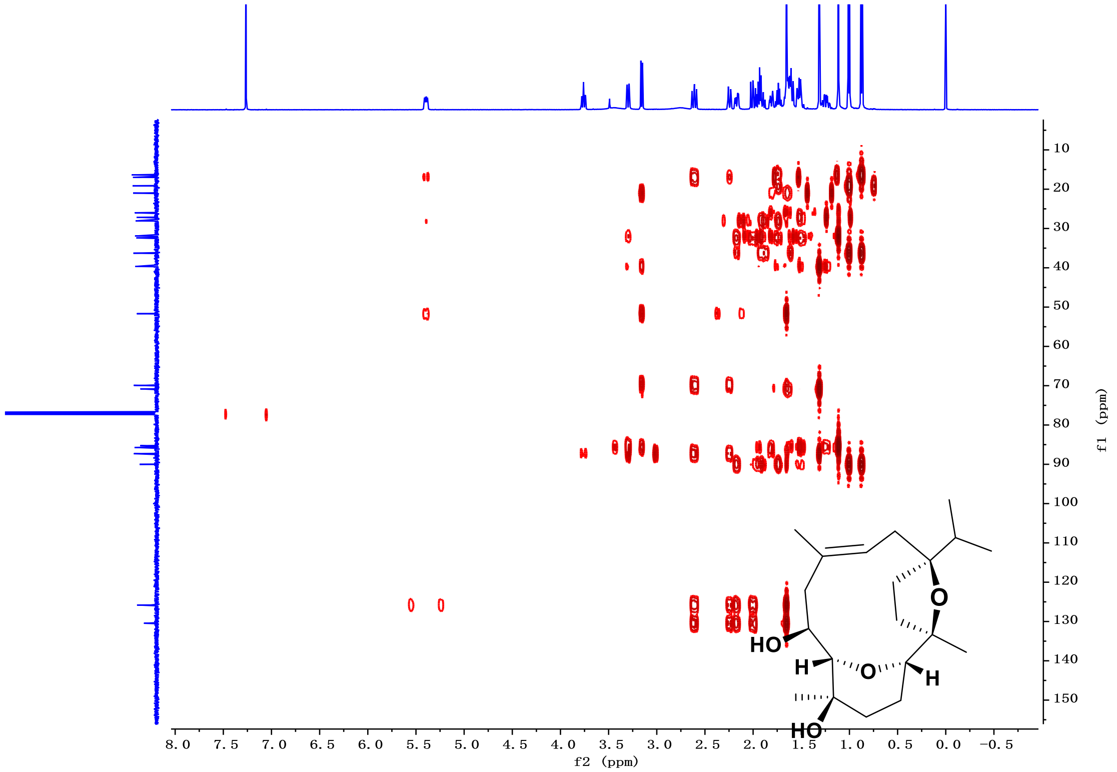

Figure S45. HMBC spectrum of **2** ($CDCl_3$, 500 MHz).

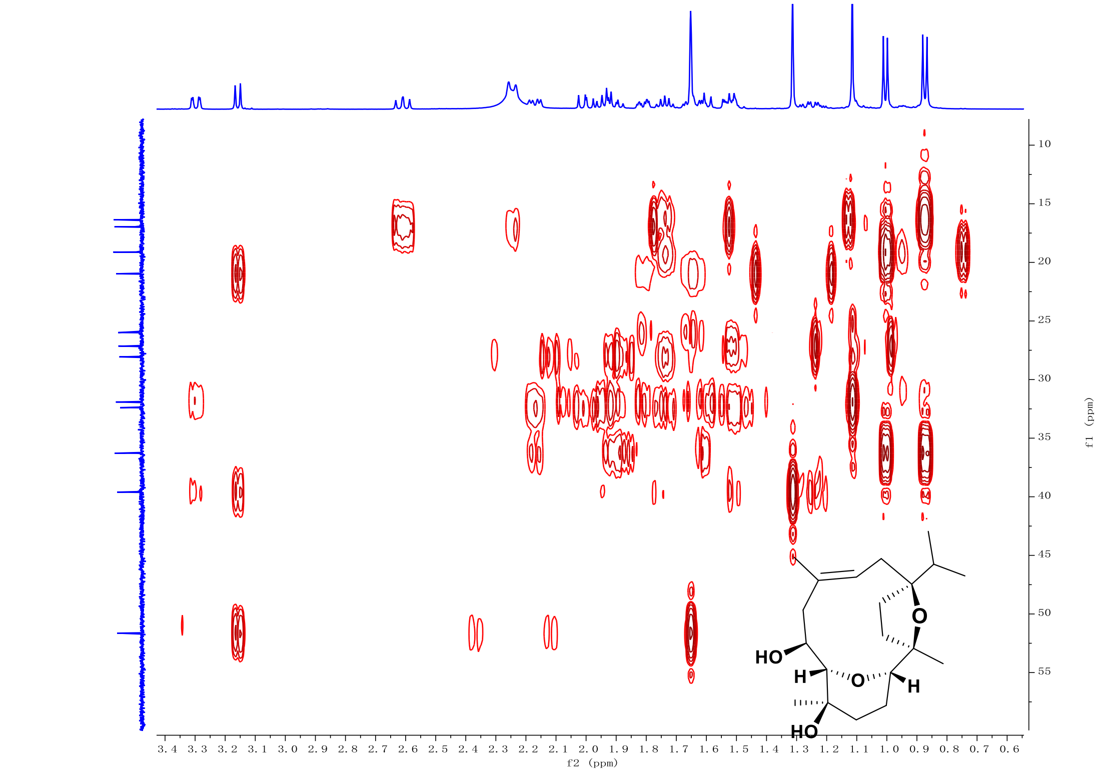

Figure S46. HMBC expansion spectrum of **2** ($CDCl_3$, 500 MHz).

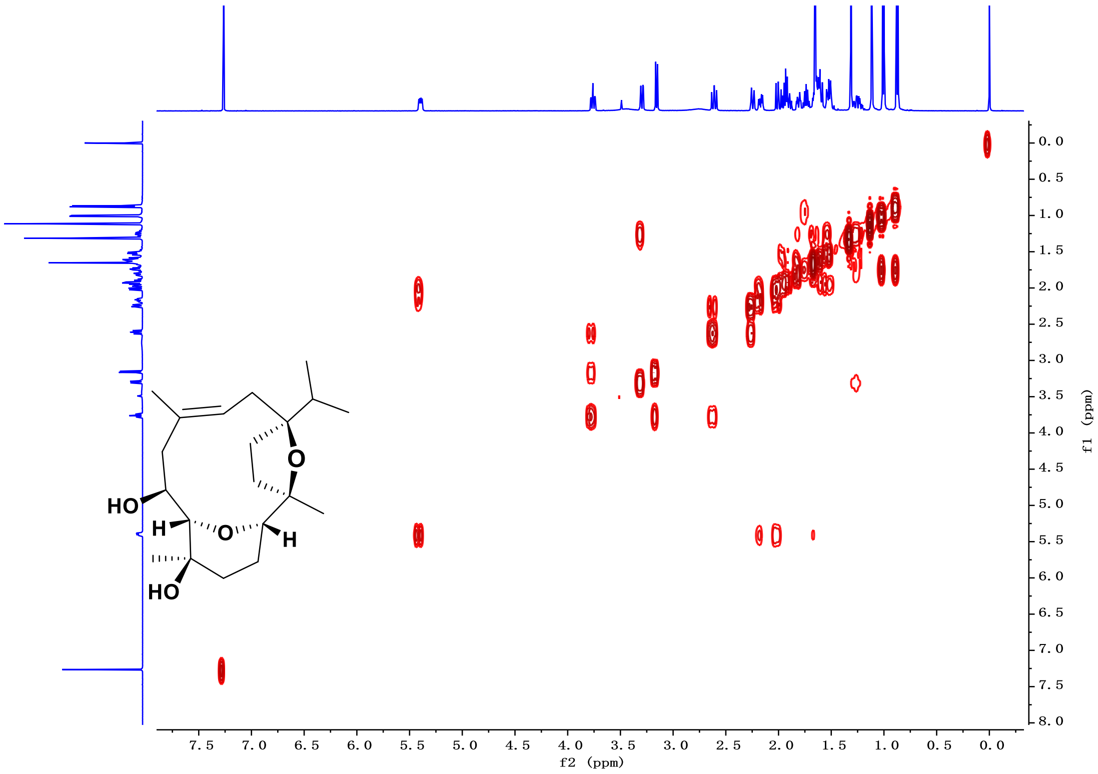

Figure S47. COSY spectrum of **2** ($CDCl_3$, 500 MHz).

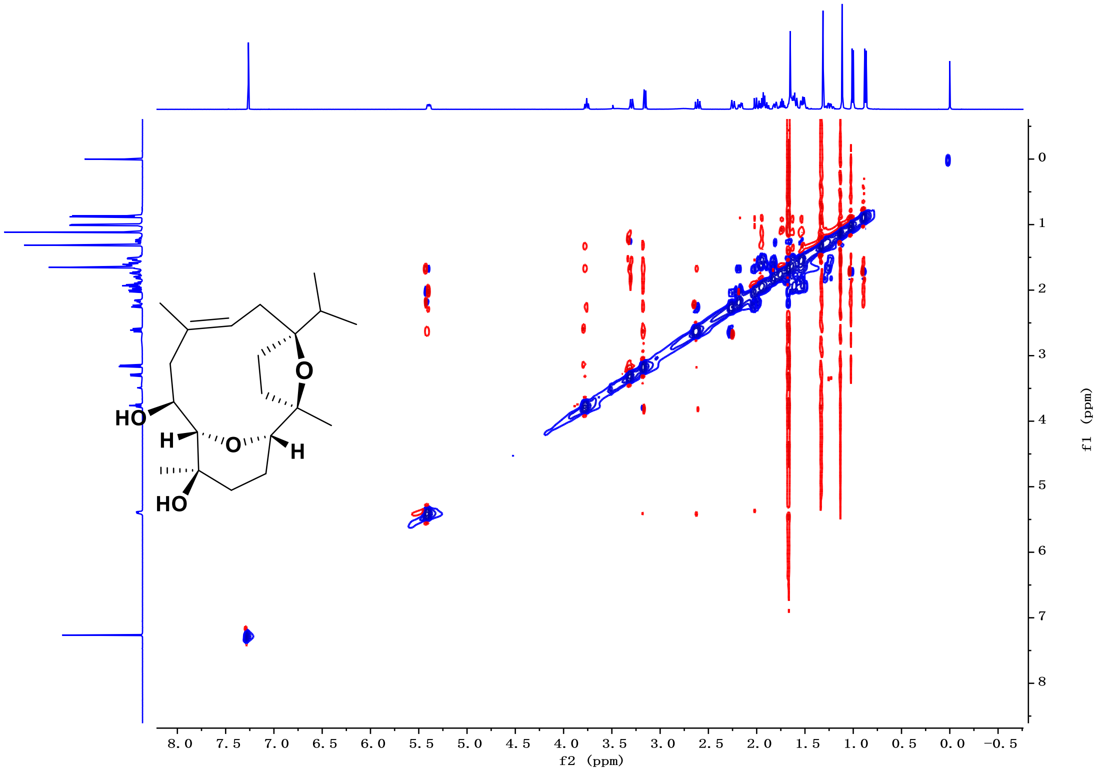

Figure S48. ROESY spectrum of **2** ($CDCl_3$, 500 MHz).

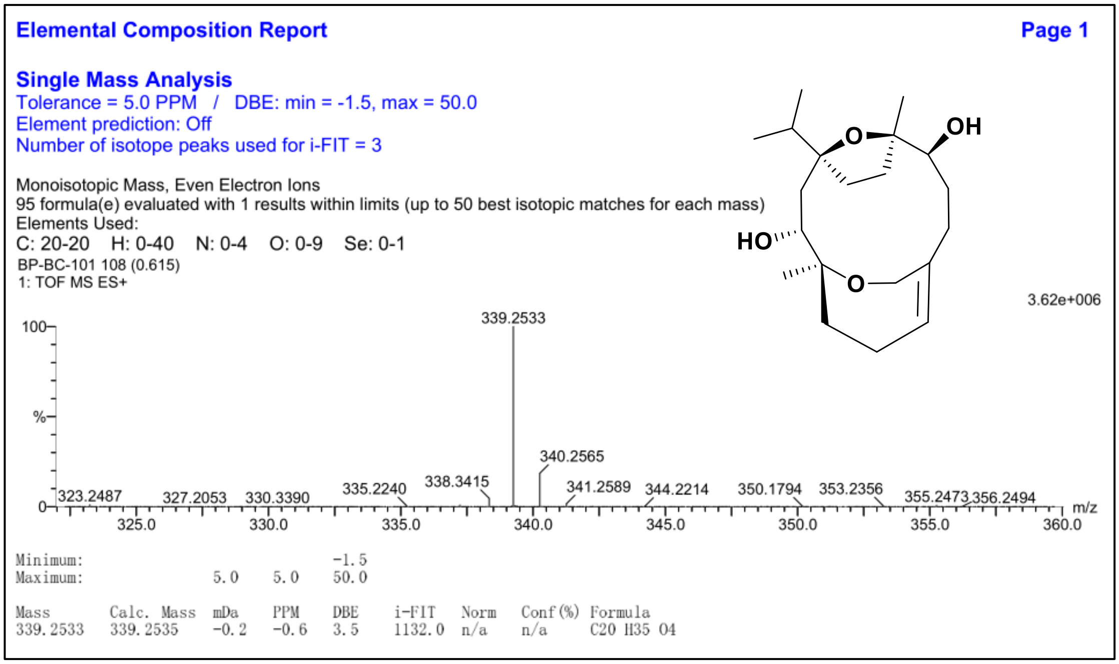

Figure S49. ESIHRMS of **3**.

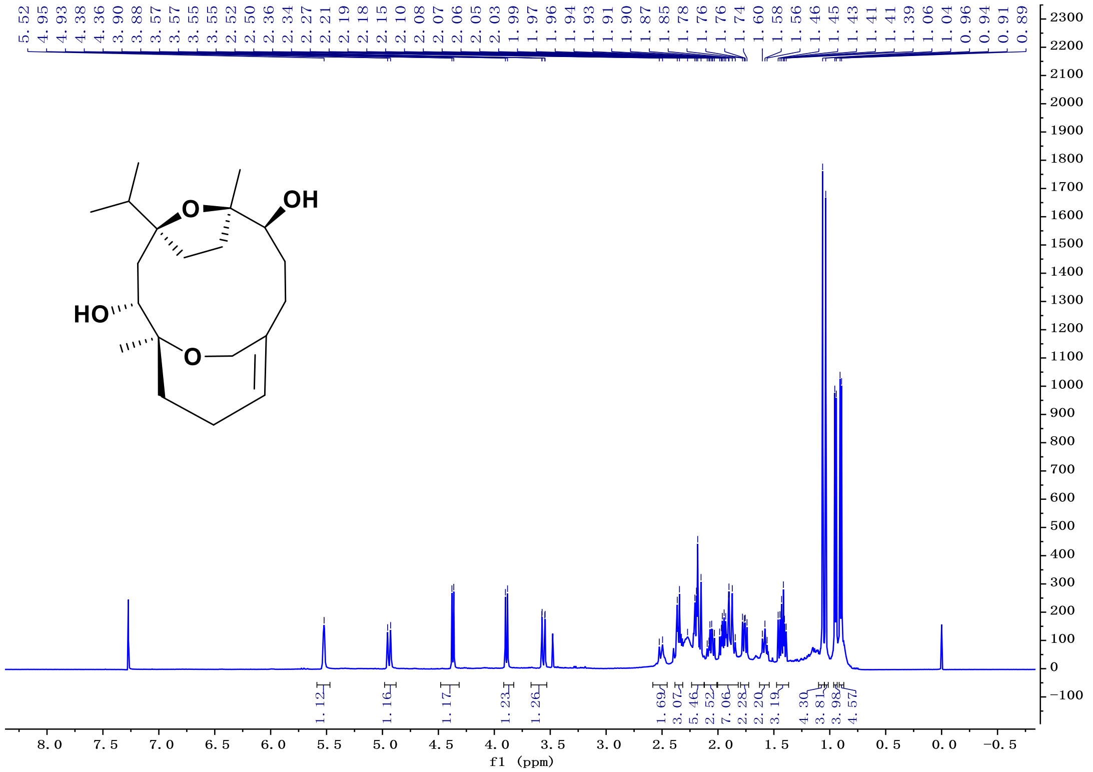

Figure S50. ^{1}H NMR spectrum of **3** ($CDCl_3$, 500 MHz).

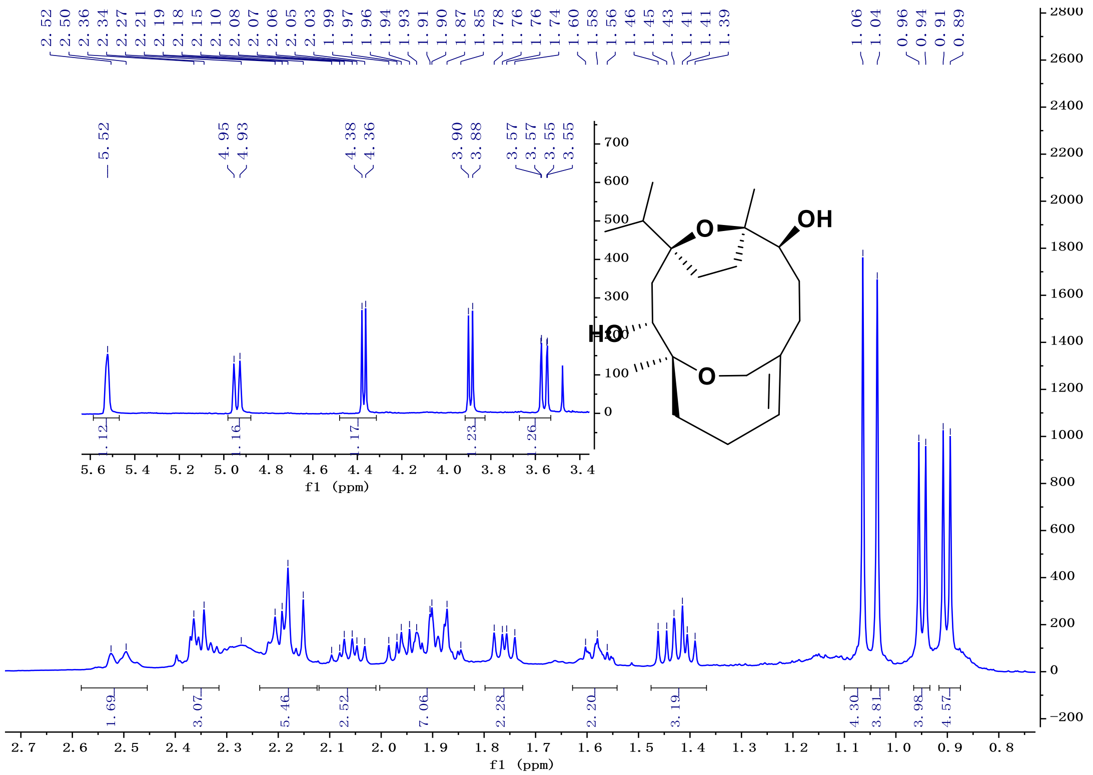

Figure S51. ^{1}H NMR expansion spectrum of **3** ($CDCl_3$, 500 MHz).

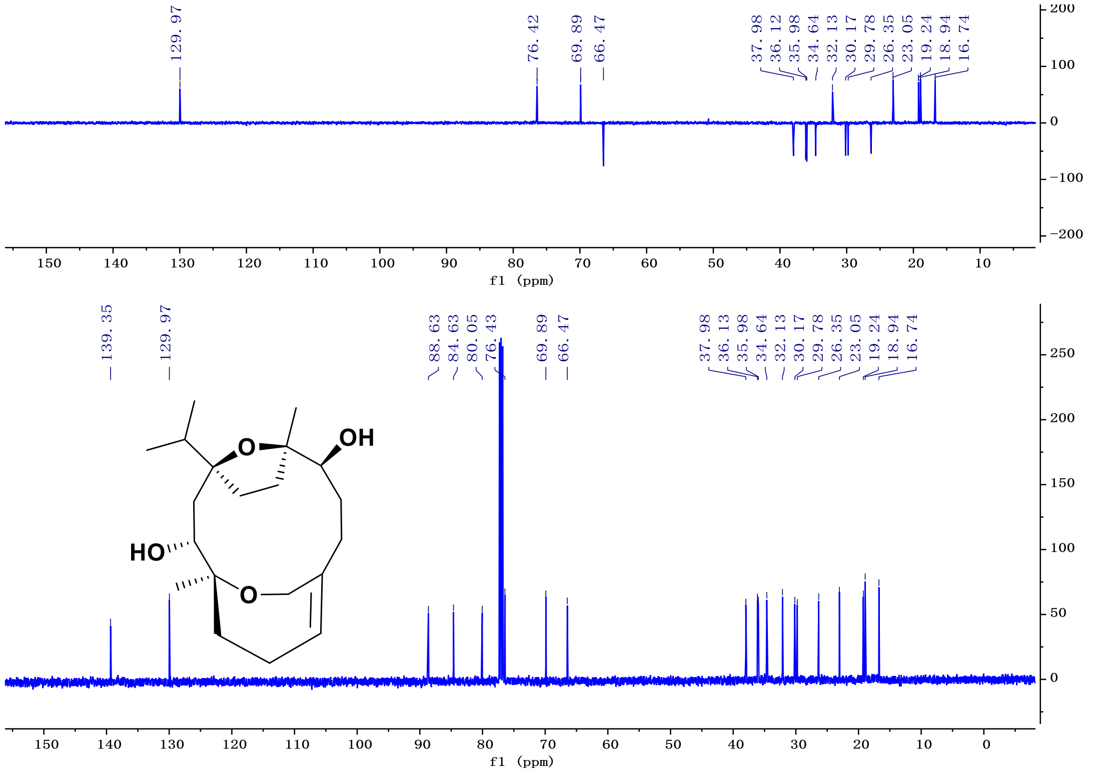

Figure S52. ^{13}C and DEPT-135 NMR spectra of **3** ($CDCl_3$, 125 MHz).

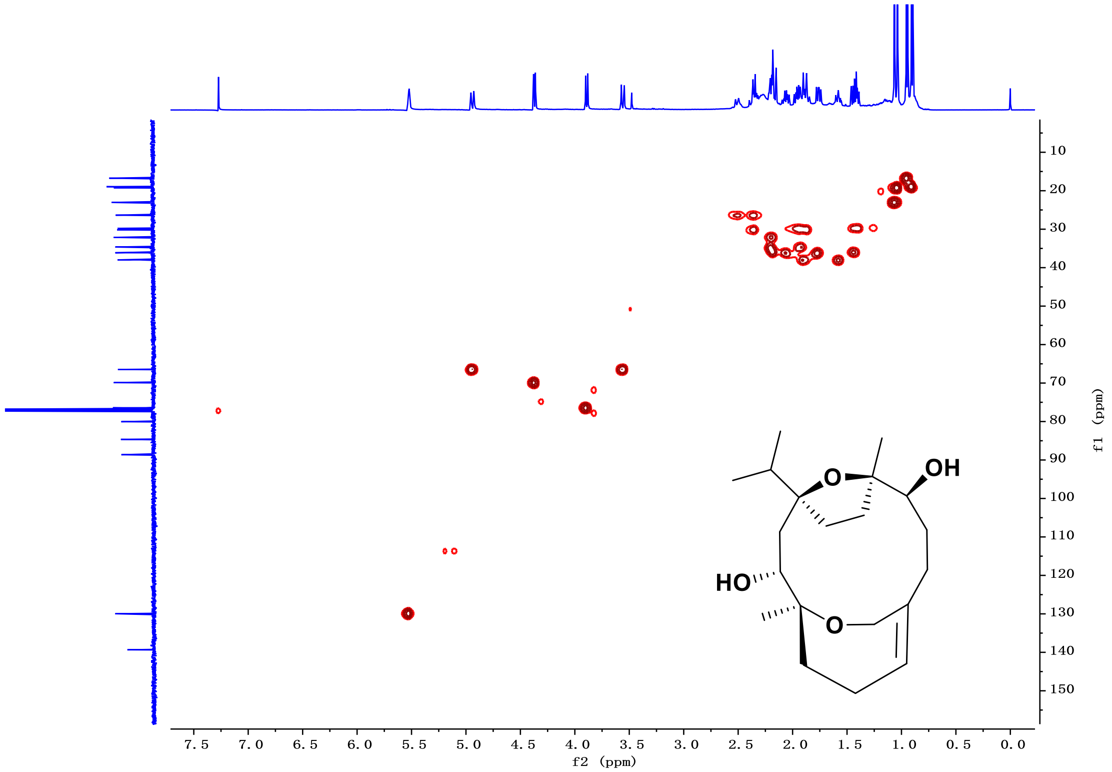

Figure S53. HSQC spectrum of **3** ($CDCl_3$, 500 MHz).

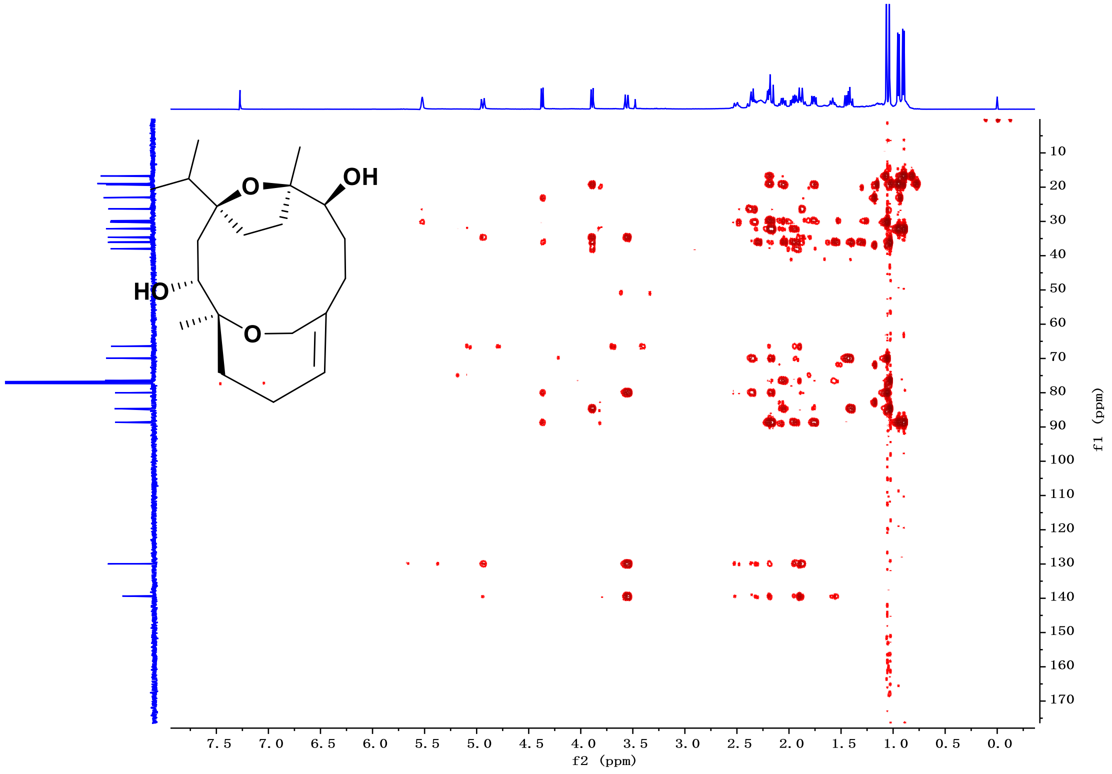

Figure S54. HMBC spectrum of **3** ($CDCl_3$, 500 MHz).

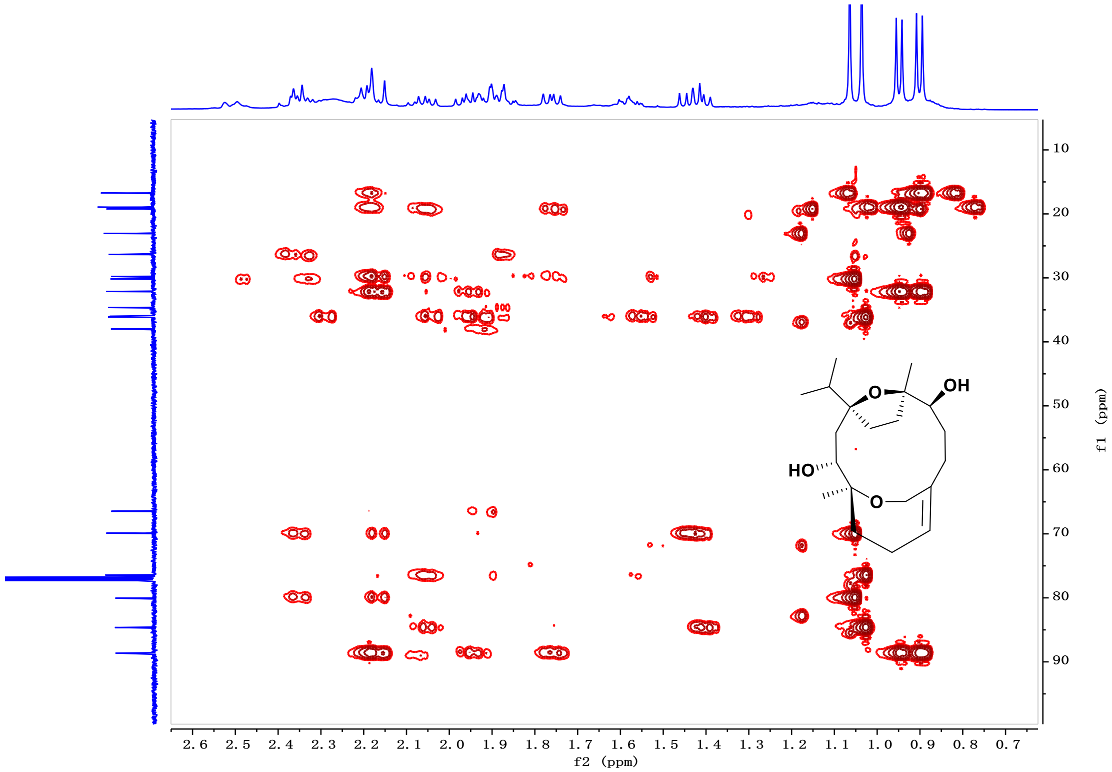

Figure S55. HMBC expansion spectrum of **3** ($CDCl_3$, 500 MHz).

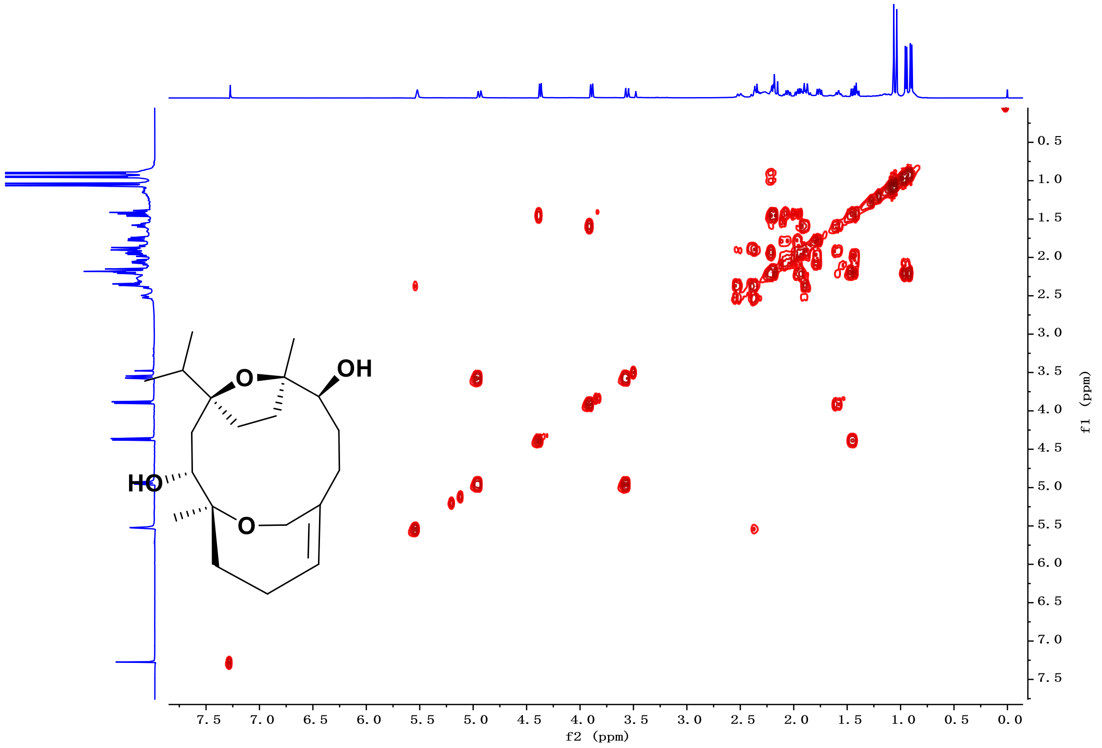

Figure S56. COSY spectrum of **3** ($CDCl_3$, 500 MHz).

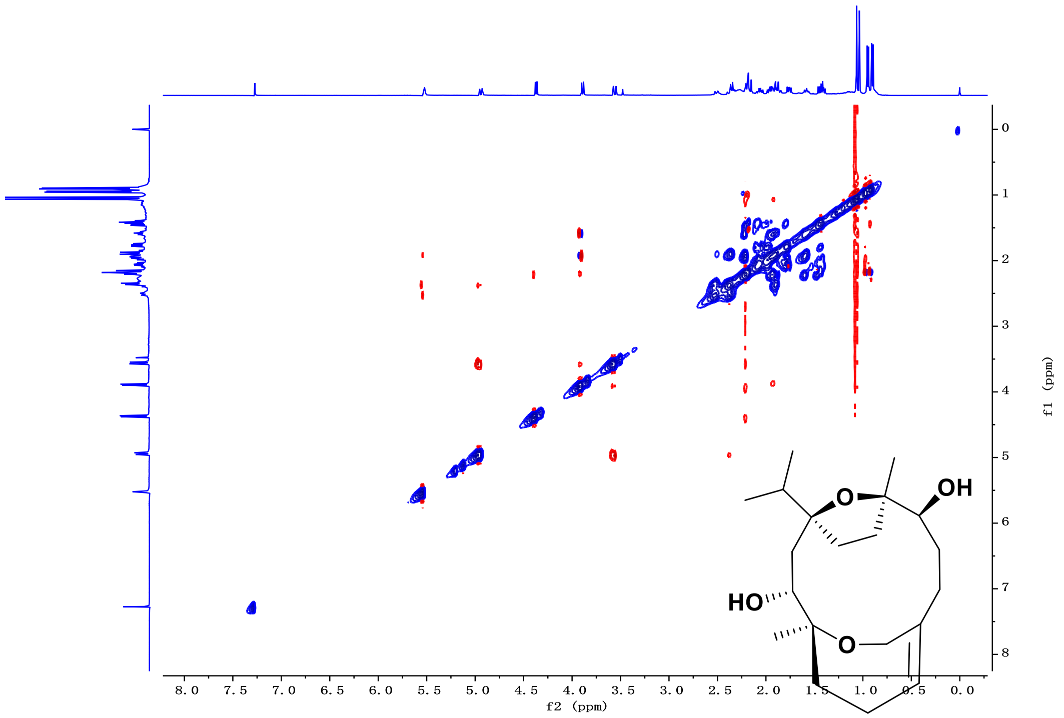

Figure S57. ROESY spectrum of **3** ($CDCl_3$, 500 MHz).

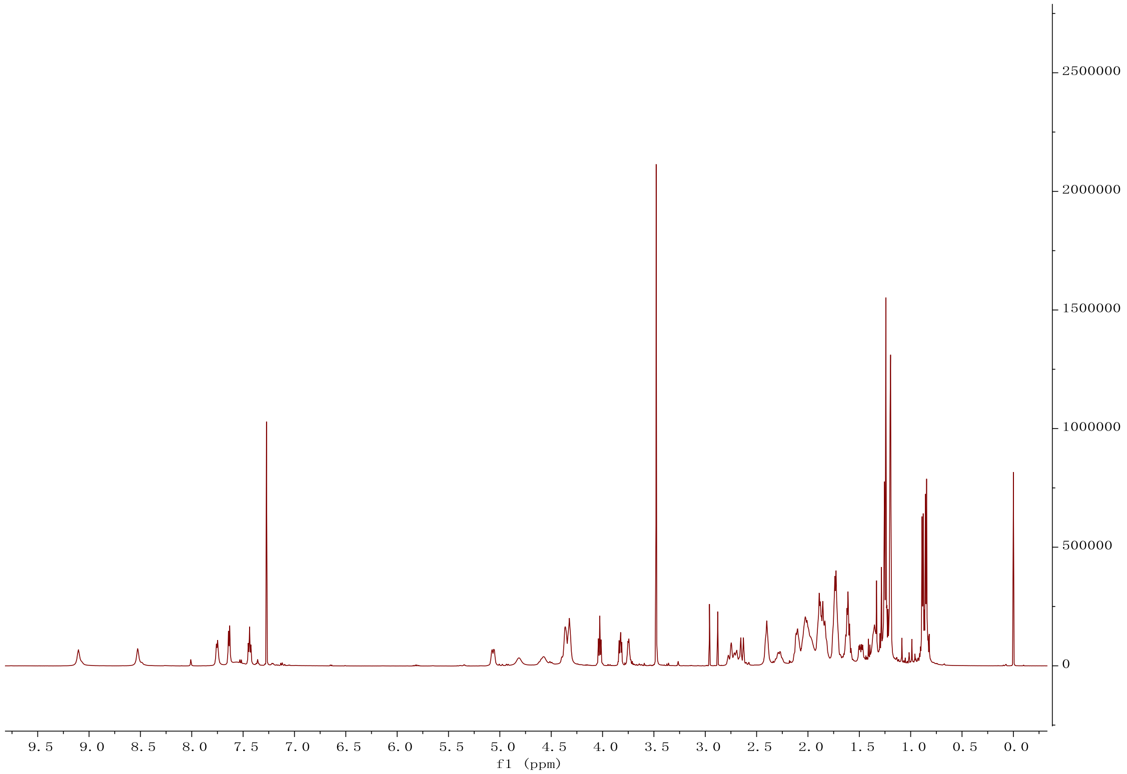

Figure S58. ^{1}H NMR spectrum of **1l** ($CDCl_3$, 600 MHz).

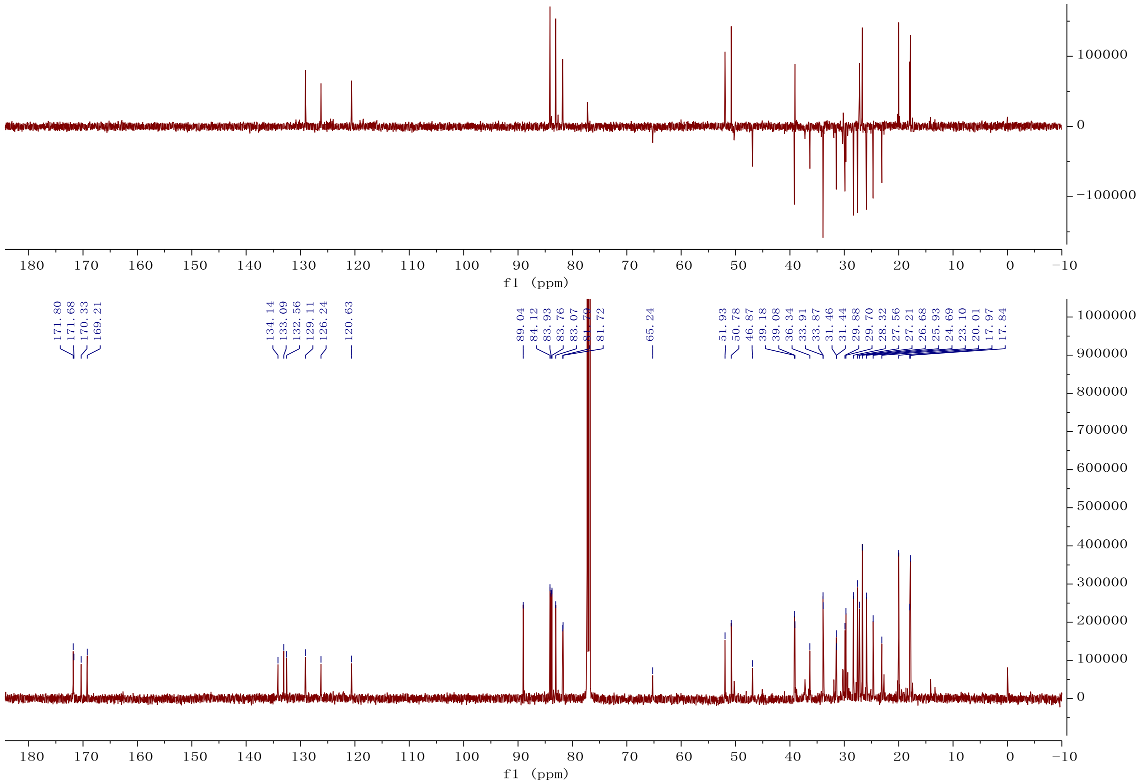

Figure S59. ^{13}C and DEPT-135 NMR spectra of **1l** ($CDCl_3$, 150 MHz).

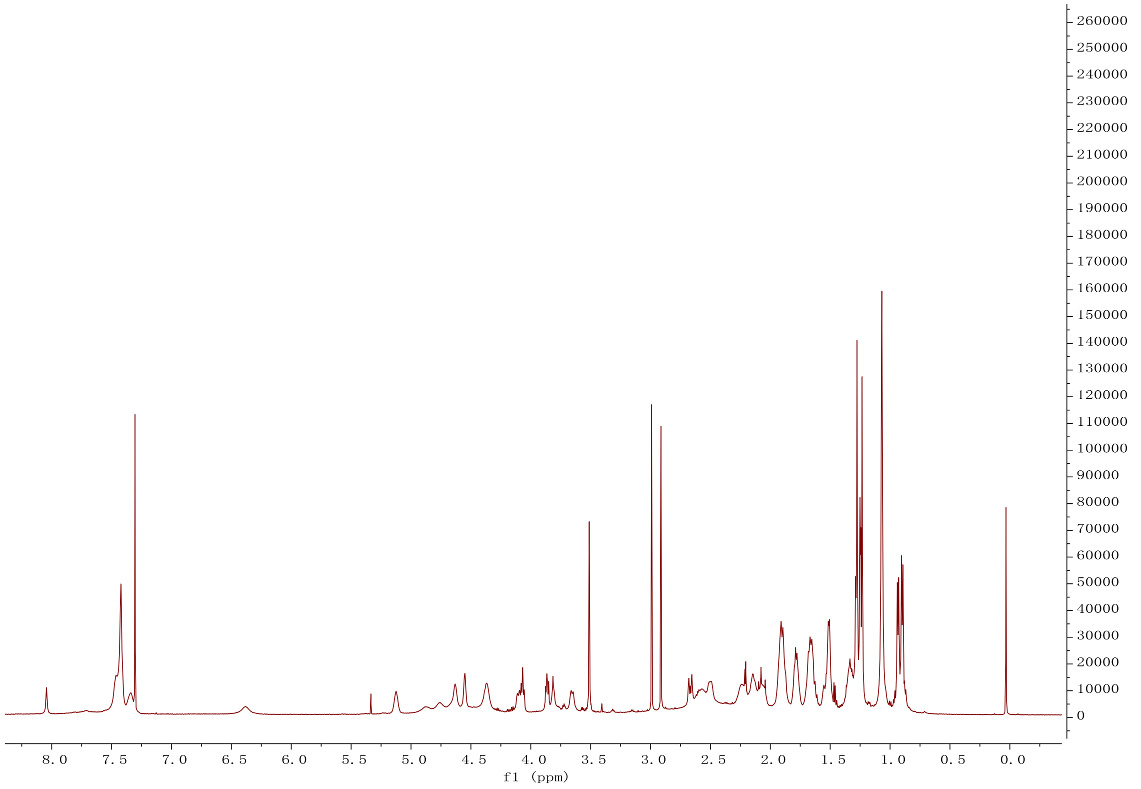

Figure S60. ^{1}H NMR spectrum of **1J** ($CDCl_3$, 600 MHz).

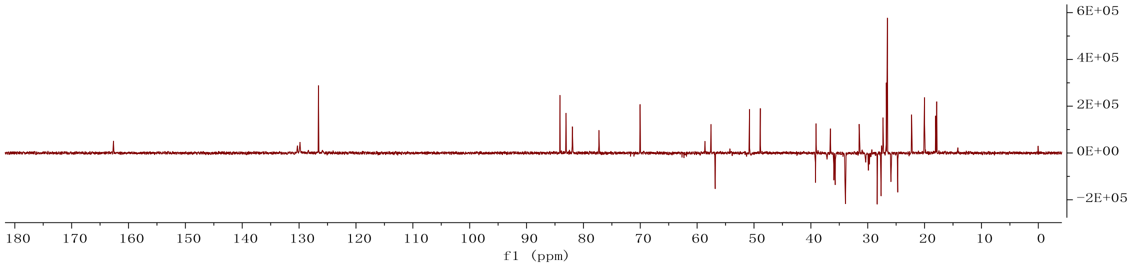

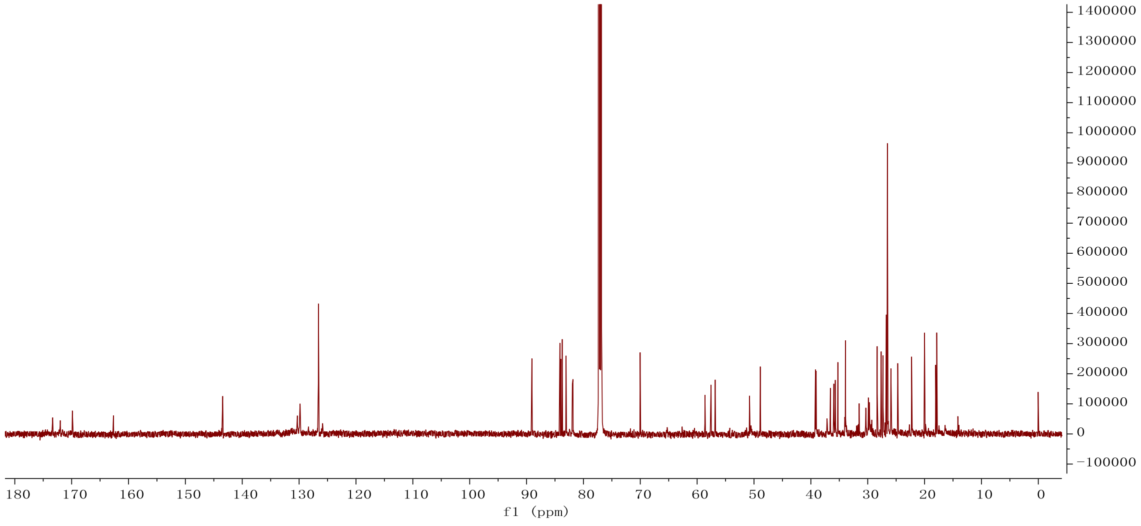

Figure S61. ^{13}C and DEPT-135 NMR spectra of **1J** ($CDCl_3$, 150 MHz).

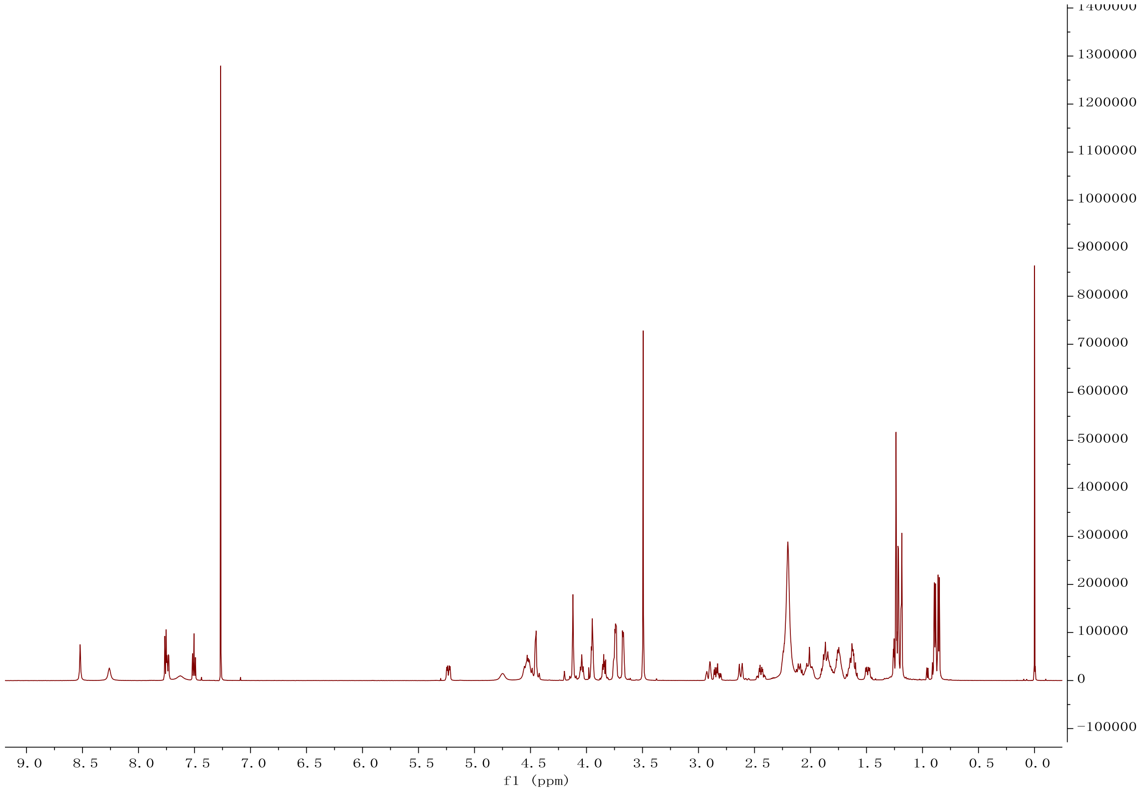

Figure S62. ^{1}H NMR spectrum of **1K** ($CDCl_3$, 600 MHz).

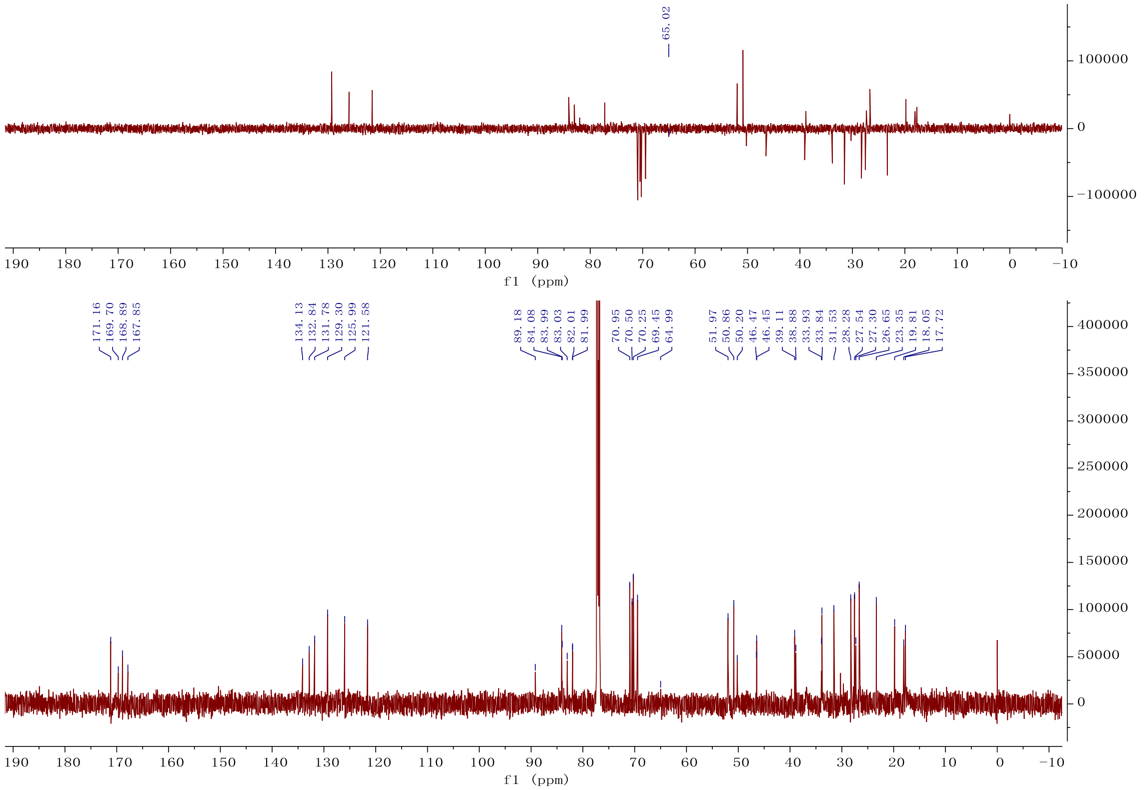

Figure S63. ^{13}C and DEPT-135 NMR spectra of **1K** ($CDCl_3$, 150 MHz).

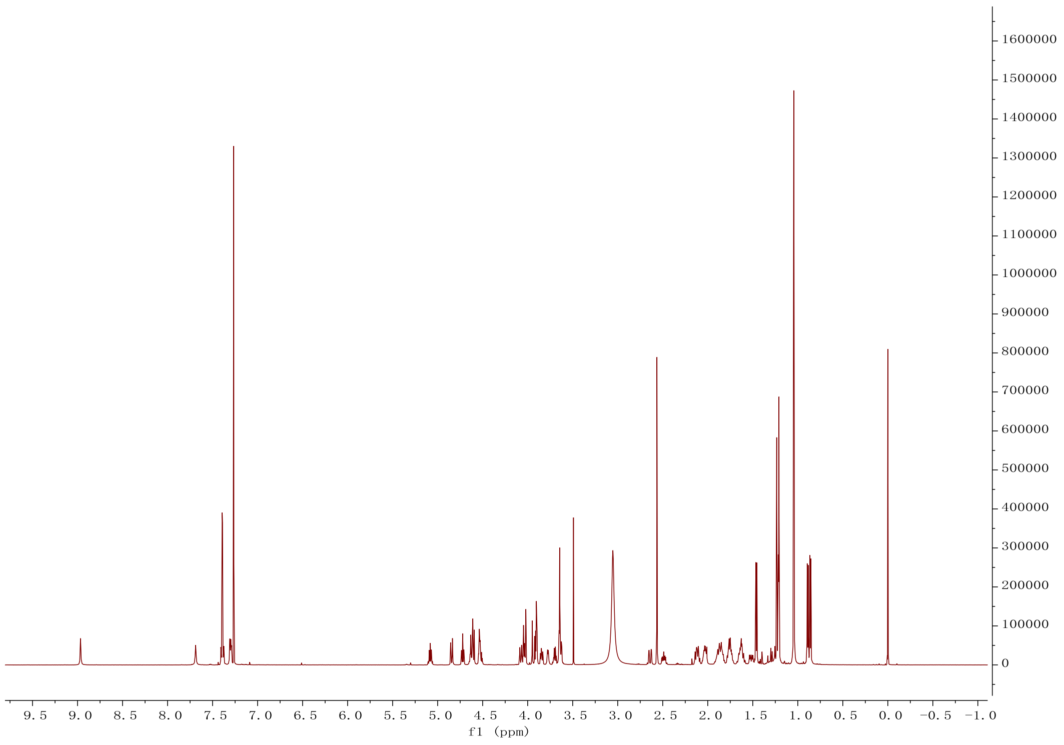

Figure S64. ^{1}H NMR spectrum of **1L** ($CDCl_3$, 600 MHz).

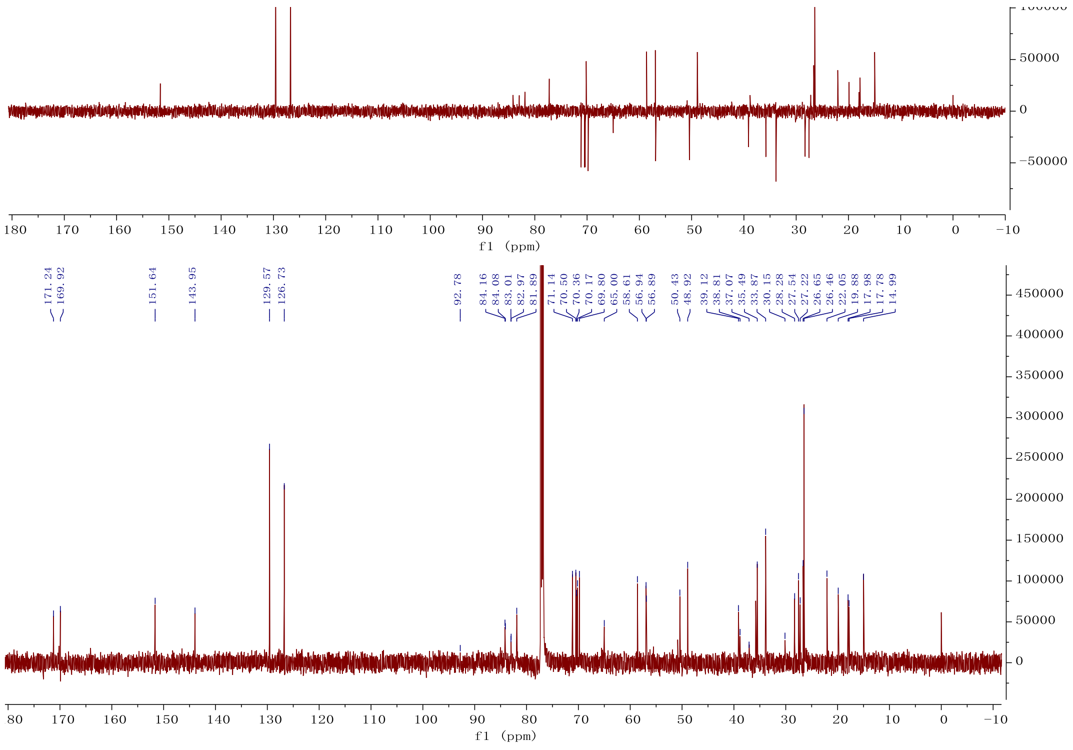

Figure S65. ^{13}C and DEPT-135 NMR spectra of **1L** ($CDCl_3$, 150 MHz).
